# Reversible Control of Native GluN2B-Containing NMDA Receptors with Visible Light

**DOI:** 10.1021/acschemneuro.4c00247

**Published:** 2024-09-06

**Authors:** Chloé Geoffroy, Romain Berraud-Pache, Nicolas Chéron, Isabelle McCort-Tranchepain, Julia Doria, Pierre Paoletti, Laetitia Mony

**Affiliations:** † Institut de Biologie de l’Ecole Normale Supérieure (IBENS), Ecole Normale Supérieure, CNRS, INSERM, Université PSL, Paris F-75005, France; ‡ Laboratoire d’Archéologie Moléculaire et Structurale (LAMS), CNRS UMR 8220, Sorbonne Université, Paris 75005, France; § PASTEUR, Département de chimie, École normale supérieure, CNRS, Université PSL, Sorbonne Université, Paris 75005, France; ∥ Laboratoire de Chimie et Biochimie Pharmacologiques et Toxicologiques, CNRS UMR8601, Université Paris Cité, Paris 75006, France

**Keywords:** glutamate, NMDA receptors, optopharmacology, allostery, GluN2B-selective antagonists, azobenzenes

## Abstract

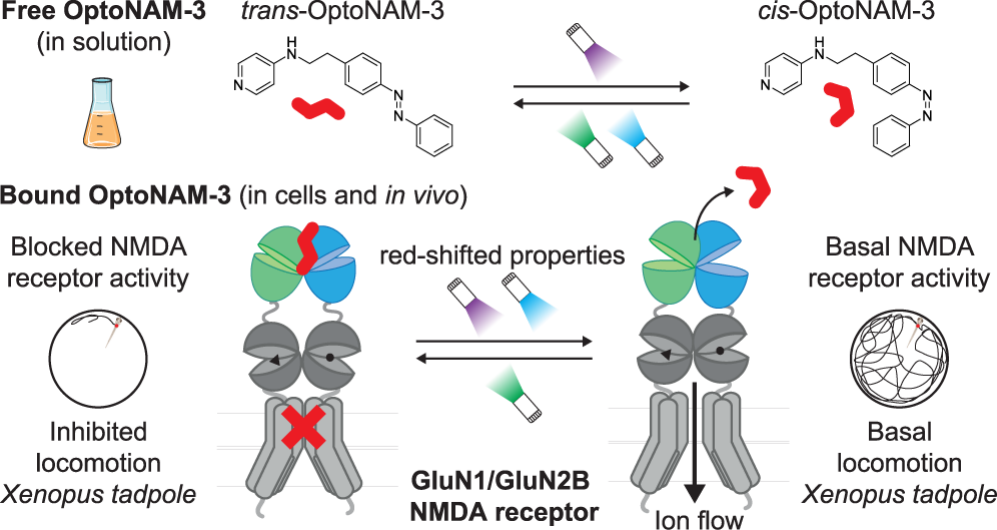

NMDA receptors (NMDARs) are glutamate-gated ion channels playing a central role in synaptic transmission and plasticity. NMDAR dysregulation is linked to various neuropsychiatric disorders. This is particularly true for GluN2B-containing NMDARs (GluN2B-NMDARs), which have major pro-cognitive, but also pro-excitotoxic roles, although their exact involvement in these processes remains debated. Traditional GluN2B-selective antagonists suffer from slow and irreversible effects, limiting their use in native tissues. We therefore developed OptoNAM-3, a photoswitchable negative allosteric modulator selective for GluN2B-NMDARs. OptoNAM-3 provided light-induced reversible inhibition of GluN2B-NMDAR activity with precise temporal control both in vitro and in vivo on the behavior of freely moving *Xenopus* tadpoles. When bound to GluN2B-NMDARs, OptoNAM-3 displayed remarkable red-shifting of its photoswitching properties allowing the use of blue light instead of UV light to turn-off its activity, which we attributed to geometric constraints imposed by the binding site onto the azobenzene moiety of the ligand. This study therefore highlights the importance of the binding site in shaping the photochemical properties of azobenzene-based photoswitches. In addition, by enabling selective, fast, and reversible photocontrol of native GluN2B-NMDARs with in vivo compatible photochemical properties (visible light), OptoNAM-3 should be a useful tool for the investigation of the GluN2B-NMDAR physiology in native tissues.

## Introduction

Neuronal plasticity, the brain’s ability to continually adapt to its environment or experiences, hinges on the dynamics of chemical synapses. At these specialized neuronal sites, neurotransmitters released from a presynaptic neuron cross the synaptic cleft and activate receptors on the postsynaptic neuron, hence mediating the transmission of information from one neuron to another. *N*-Methyl-d-aspartate receptors (NMDARs) are a class of ionotropic receptors activated by glutamate, the main excitatory neurotransmitter of the vertebrate central nervous system. They play a central role in synaptic transmission and plasticity, but their dysfunction is also involved in many pathologies.^1−3^ NMDARs are tetramers composed of two GluN1 and two GluN2 (or GluN3) subunits. Each tetramer can either incorporate two identical GluN2 (or GluN3) subunits (diheteromers) or different GluN2 (or GluN3) subunits (triheteromers), each GluN2 subunit conferring to the receptor distinct biophysical and pharmacological properties, as well as different expression and signaling profiles.^1,2^ Understanding the functional role of NMDAR individual subtypes in the brain is fundamental to developing new strategies to counteract the deleterious effects of NMDAR deregulation.

Overactivation of NMDARs, as occurring during traumatic brain injury or stroke, induces an excessive increase in intracellular calcium, a process leading to neuronal death (excitotoxicity).^4^ This excitotoxicity phenomenon is also observed in neurodegenerative diseases like Alzheimer’s and Parkinson’s diseases.^1,2,5,6^ NMDAR overactivation furthermore occurs in other pathologies such as epilepsy, neuropathic pain, and depression.^1,2,5,7^ Multiple studies point to a specific role of GluN2B-containing NMDARs (GluN2B-NMDARs) in triggering excitotoxicity, although this role is debated.^1,5,6^ To counteract the deleterious effects of GluN2B-NMDAR overactivation, a large number of negative allosteric modulators (NAMs) specific for GluN2B-NMDARs were developed in the late 90s to early 2000s.^5,8−11^ These antagonists, of which ifenprodil is the lead compound,^12,13^ displayed neuroprotective properties in vitro and in vivo with reduced adverse effects compared to broad spectrum antagonists.^1,5,10,14−16^ So far, however, all of these compounds failed in clinical trials because of a lack of effect or a narrow therapeutic window.^5,16,17^

Ifenprodil derivatives bind at the level of the NMDAR N-terminal domains (NTDs),^18,19^ which are bilobar domains preceding the agonist-binding domain (ABD) and constitute a hub for allosteric modulation in NMDARs^2,3,5,8^ (see Figure 3A for the NMDAR subunit architecture). At this level, these compounds induce their inhibition by interacting with the upper lobe of GluN1 NTD and with the upper and lower lobes of GluN2B NTD,^18−20^ which favors the entry of NMDARs into an inhibited state.^20−24^ Some of these GluN2B-specific NAMs, such as ifenprodil, Ro25–6981,^25^ or CP-101606,^26^ are currently used as standard pharmacological tools to specifically target GluN2B-NMDARs in native tissues and have proven useful in investigating the contributions of this receptor subtype to several physiological and pathological processes. However, the use of these compounds in native tissues faces serious limitations due to their slow dissociation kinetics. In recombinant systems, time constants of dissociation of GluN2B-specific NAMs are in the tens of seconds to minute time range.^27,28^ These slow kinetics are even more marked in native tissues. In brain slices, for instance, relief from inhibition by GluN2B-specific NAMs is so slow that it is considered irreversible. It is thus important to develop GluN2B-selective inhibitors with improved temporal resolution and reversibility of action for a dynamic control of GluN2B-NMDARs in native tissues.

Photopharmacology, an approach based on the use of photosensitive ligands, allows such high temporal resolution of action. The most widely adopted method for optical modulation of ion channel activity involves caged compounds, whose activity is inhibited by a photolabile moiety (cage), but this strategy is limited by its irreversibility.^29,30^ An alternative approach employs photoconvertible ligands that can alternate between an active and an inactive configuration after exposure to different light wavelengths.^29−31^ This photoswitchable property is conferred to the molecule by the presence of a photoisomerisable unit such as an azobenzene, which can reversibly alternate between an extended *trans* and a twisted *cis* configuration using two different wavelengths, usually UV and blue–green light.^29−32^ In a previous paper, we had developed a caged and a photoswitchable ifenprodil derivative.^33^ However, while caged ifenprodil allowed fast GluN2B-NMDAR inhibition upon UV irradiation, the kinetics of NMDAR recovery from inhibition were still limited by the slow dissociation rate of ifenprodil. Additionally, the strategy we used to obtain a photoswitchable ifenprodil – addition of an azobenzene to the ifenprodil molecule (azo-extension approach)^34^ – strongly decreased its inhibitory activity, probably because the ifenprodil binding site is too small to accommodate the supplementary azobenzene moiety.^33^

In this paper, we took advantage of the chemical diversity of GluN2B-selective antagonists^5,9−11,19,35^ and designed photoswitchable NAMs by incorporating the azobenzene moiety within the chemical scaffold of the molecule (azologization approach).^34,36−38^ Among the four photoswitchable NAM candidates, OptoNAM-3 appeared as a potent and selective inhibitor of GluN2B-NMDARs in its *trans* configuration, while its *cis* isomer was inactive. OptoNAM-3 allowed real-time and reversible control of GluN2B-NMDAR activity with fast (in the second range) temporal resolution. Surprisingly, binding of OptoNAM-3 to GluN2B-NMDARs induced a red-shift of its action spectrum, allowing us to use visible (blue) light instead of UV light to turn off OptoNAM-3 activity. We finally show that OptoNAM-3 also acts as a red-shifted photomodulator in vivo, allowing us to reversibly modulate the locomotion behavior of *Xenopus* tadpoles. This highlights the strong potential of OptoNAM-3 for fast and reversible control of GluN2B-NMDAR activity in vivo.

## Results

### Design and Synthesis of a Photoswitchable, GluN1/GluN2B-Selective NMDAR Antagonist

**Figure 1:**
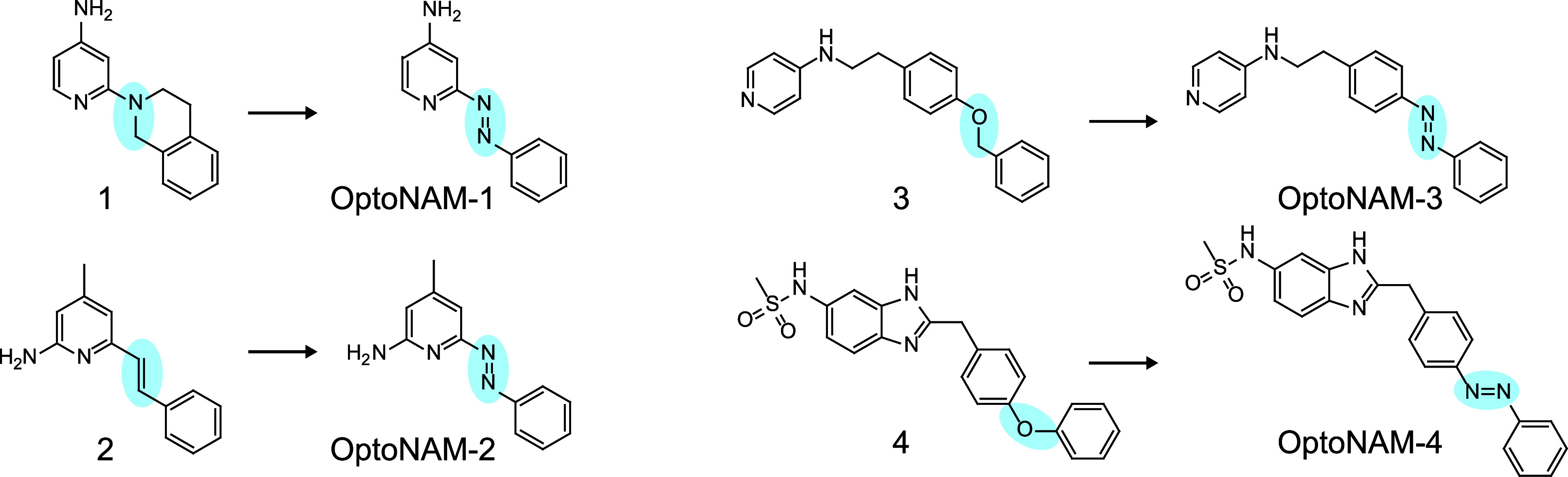
Design of photoswichable NAMs for GluN1/GluN2B receptors. Chemical structures of published GluN2B-selective NAMs (parent compounds **1**–**4**) and their photoswitchable equivalents (OptoNAM-1 to OptoNAM-4) designed by substituting an “azostere” moiety of the parent compound (blue circle) by an azo moiety. Compounds **1**, **2**, **3**, and **4** correspond to compounds **9n**,^39^**14**,^40^**11**,^41^ and **17a**,^42^ respectively.

**Scheme 1:**
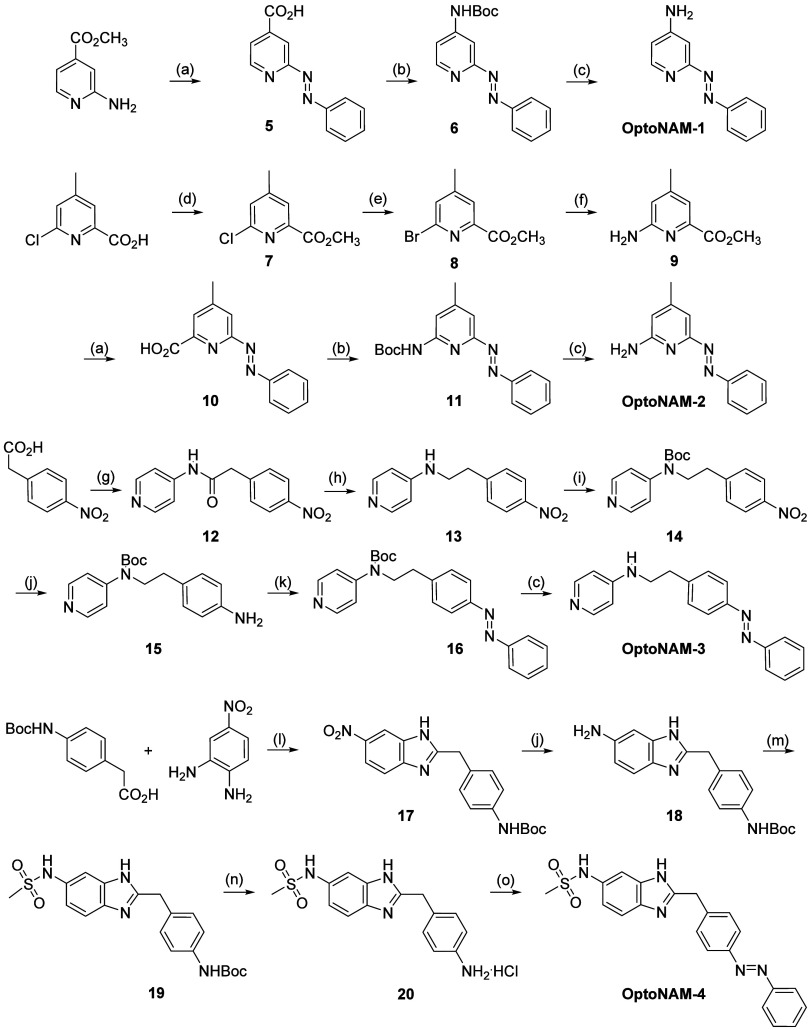
Synthesis of OptoNAM-1 to OptoNAM-4^a^ Reagents and conditions: (a) nitrobenzene, 5 M NaOH_aq_ in toluene, 50 °C for 3 h and then 80 °C for 12 h, 72–80%; (b) DPPA, Et_3_N, *t*BuOH in toluene, 75 °C for 3 h and then 100 °C for 12 h, 70–75%; (c) 6 M HCl_aq_, MeOH, RT for 16 h, 47–51%; (d) SOCl_2_, MeOH, reflux for 16 h, 91%; (e) TMSBr, propionitrile, reflux for 4 h, 99%; (f) benzophenone imine, Cs_2_CO_3_, toluene, Pd_2_(dba)_3_, and BINAP, 90 °C for 12 h, 47%; (g) 4-aminopyridine, DCC, THF, RT for 16 h, 68%; (h) borane dimethyl sulfide, RT for 2 h and then 65 °C for 3 h, 59%; (i) Boc_2_O, DMAP, THF, 65 °C for 3 h, 100%; (j) 10% Pd/C and H_2_, RT for 16 h, 100%; (k) nitrobenzene, AcOH, RT for 8 h, 70%; (l) methyl chloroformate, Et_3_N, DMF, 20 °C for 4 h, 44%; (m) pyridine, CH_3_SO_2_Cl, CH_2_Cl_2_, RT for 16 h, 95%; (n) MeOH, 4 M HCl in dioxane, RT for 12 h, 100%; (o) KOAc, glacial acetic acid, nitrobenzene, RT for 8 h, 19%.

To generate photoswitchable GluN2B-selective NAMs, we selected a series of four NAMs selective for GluN2B-NMDARs^9,39−42^ possessing chemical motifs that can be changed into an azobenzene with minimal perturbation of the molecule structure (isosteres of azobenzenes or “azosteres”).^34,36^ This led to the design of photoswitchable compounds OptoNAM-1 to OptoNAM-4 (Figure 1 and Table S1), which were obtained according to a custom synthesis by Enamine Ltd. (Kiev, Ukraine) carried out according to Scheme 1. The synthesis of OptoNAM-1 to OptoNAM-4 is described in Methods, and NMR and HPLC–MS spectra are shown in Spectra S1–S4.

**Figure 2:**
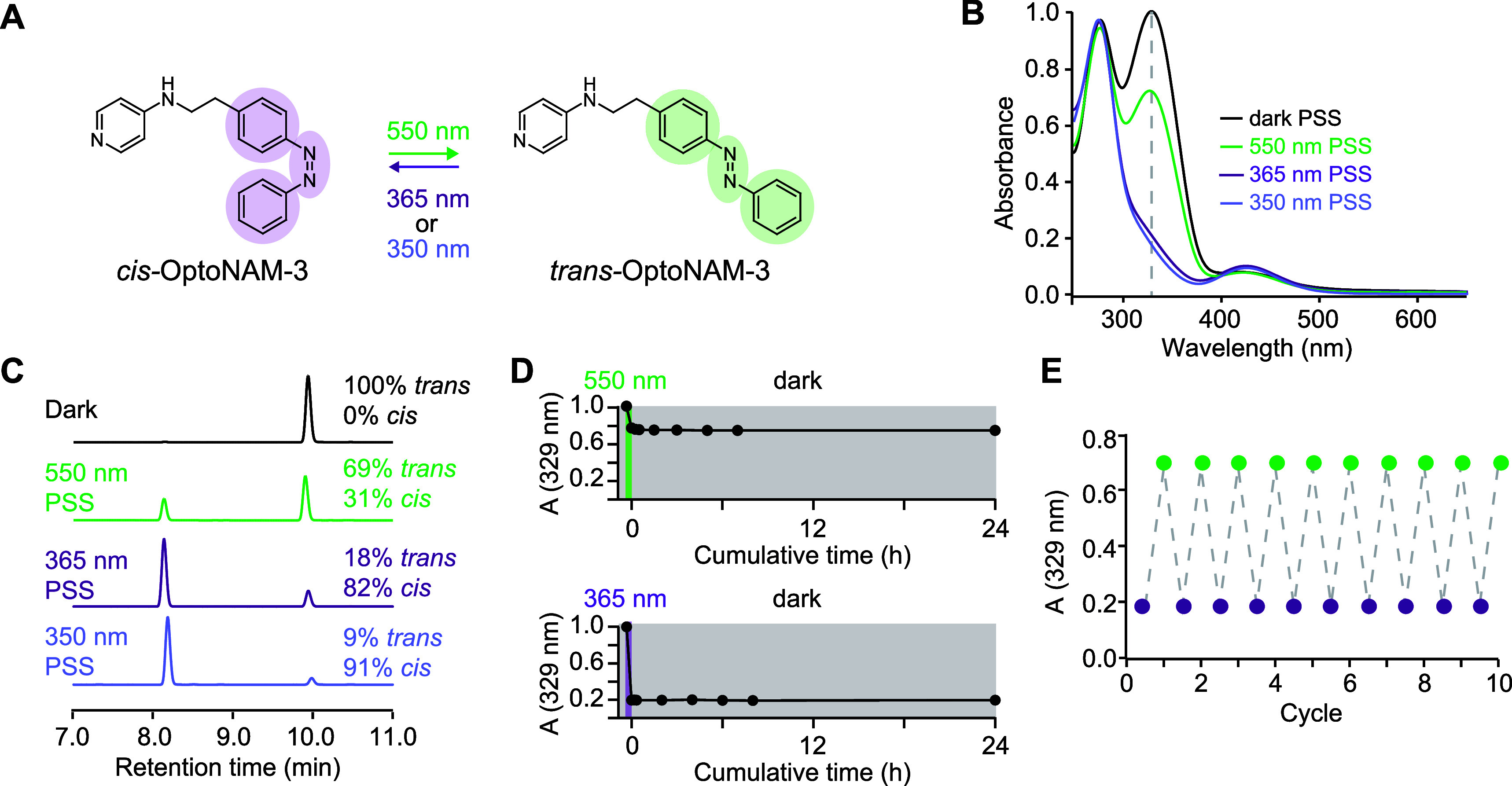
Photochemical properties of OptoNAM-3. (A) OptoNAM-3 can effectively be switched from *trans* to *cis* configuration by UV illumination (365 or 350 nm) and back to *trans* configuration by 550 nm light. (B) UV–visible absorption spectra of OptoNAM-3 in physiological aqueous buffer (Ringer pH 7.3, see Methods) in the dark, after UV illumination by 350 or 365 nm light, and after 550 nm illumination of the 365 nm PSS. The dashed line represents the peak absorption wavelength of *trans*-OptoNAM-3 (329 nm). (C) HPLC chromatograms of OptoNAM-3 in the dark, after 365 and 350 nm illumination, and after subsequent illumination by 550 nm light. The photostationary states (PSSs) at these different wavelengths were quantified and written next to the peaks corresponding to each isomer. (D) OptoNAM-3 550 and 365 nm PSS displays strong thermal stability in the dark in physiological aqueous buffer (Ringer pH 7.3, see Methods) and at room temperature: no change of their absorption spectrum was observed up to 24 h after illumination. (E) OptoNAM-3 can undergo 10 cycles of UV/green illumination without degradation. The graph represents the absorbance at 329 nm for each light condition (purple circle solids for OptoNAM-3 365 nm PSS and green circle solids for 550 nm).

The photochemical and biological characterizations of OptoNAM-1, OptoNAM-2, and OptoNAM-4 are described in Text S1, Figures S1 and S2, and Table S1. In brief, OptoNAM-1 and OptoNAM-2 displayed photodependent activity on GluN1/GluN2B NMDARs but with a > 1000-fold shift in IC_50_ compared to their parent compounds (Figure S1 and Table S1). This is likely due to the loss of protonation of the aminopyridine moiety at physiological pH induced by the introduction of the azo moiety (Figure S2). Protonation was indeed shown to be critical for the activity of this class of compounds onto GluN1/GluN2B NMDARs^39^ (Figure S2G). OptoNAM-4, on the other hand, retained strong potency for GluN1/GluN2B NMDARs, but its activity was not photodependent (Figure S1 and Table S1). In this paper, we focus on OptoNAM-3 (Figures 1 and 2A), which emerged as the best candidate for efficient photocontrol of GluN1/GluN2B NMDARs (see below).

### Photochemical Characterization of OptoNAM-3

To characterize the photochemical properties of OptoNAM-3, we focused on the three key properties allowing the use of azobenzenes in biological systems: (i) wavelengths for *trans*-to-*cis* and *cis*-to-*trans* conversions; (ii) compound thermalstability, i.e., half-life of the *cis* isomer in the dark; and (iii) fatigability, i.e., the number of illumination cycles that the molecule can undergo without degradation. To this aim, we acquired UV–visible absorption spectra of OptoNAM-3 diluted in *Xenopus* oocyte recording solution (Ringer pH 7.3; see Methods) either in the dark (absence of illumination) or after illumination by light wavelengths ranging from 350 to 580 nm (Figures 2B and S1M). In the dark (black curve in Figure 2B), the spectrum was characteristic of a *trans* isomer.^32^ Application of UV light at a wavelength close to the main absorption peak of the *trans* form (365 or 350 nm) gave a completely different spectrum (violet curves in Figure 2B), characteristic of azobenzenes in their *cis* configuration.^32^ HPLC analysis of OptoNAM-3 in solution identified photostationary states (PSS) containing 100% *trans* in the dark, 82% *cis* and 18% residual *trans* after illumination at 365 nm (365 nm PSS, violet curve in Figure 2C), and 91% *cis* and 9% residual *trans* after illumination at 350 nm (350 nm PSS, lavender curve in Figure 2C). This shows that illumination with UV light can convert a large majority of OptoNAM-3 into its *cis* configuration, with 350 nm being more efficient than 365 nm. Irradiation of OptoNAM-3 365 nm PSS with wavelengths from 435 to 550 nm gave equivalent *cis*-to-*trans* conversions with 435 and 550 nm yielding slightly stronger conversions (∼70% *trans* after illumination at 435 and 550 nm calculated from UV–visible spectra at λ_trans_ = 329 nm, Figures 2B and S1N; 550 nm PSS of 69% *trans* and 31% *cis* measured by HPLC, Figure 2C, green trace). 550 nm was chosen as the optimal wavelength since green light is less harmful for cells than lights of shorter wavelengths. We then tested the thermal stability of the *cis* form in the dark (in the absence of illumination). The absorbance spectrum of the 365 nm PSS kept in the dark in physiological buffer (Ringer) did not evolve after 24 h at room temperature (Figure 2D), indicating a very strong thermal stability of the *cis* isomer in aqueous solution in the dark. Similarly, the 550 nm PSS did not evolve over 24 h (Figure 2D). This very high stability is interesting because it avoids the prolonged use of light to maintain OptoNAM-3 in the desired configuration. OptoNAM-3 could finally endure many illumination cycles without degradation (Figure 2E).

### OptoNAM-3 Is a Potent NAM of GluN1/GluN2B NMDARs with a Photodependent Activity

**Figure 3:**
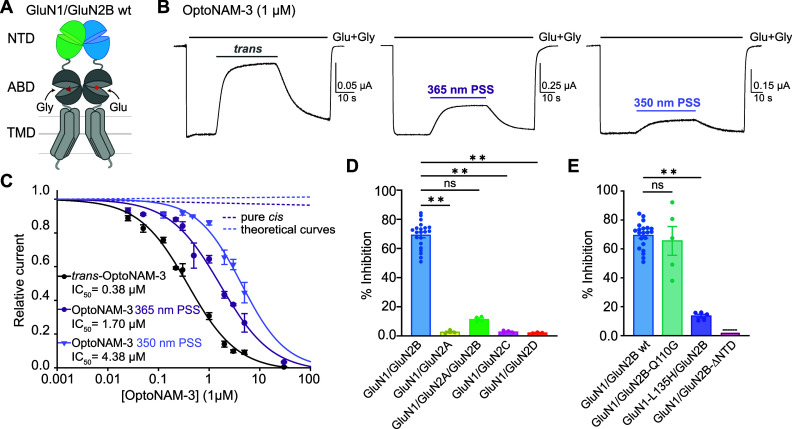
OptoNAM-3 inhibits GluN1/GluN2B selectively and in a photodependent manner. (A) Architecture of a dimer of GluN1 and GluN2B subunits with N-terminal domains (NTDs) in green and blue, respectively. ABD, agonist-binding domain; TMD, transmembrane domain. (B) Representative current traces from oocytes expressing GluN1/GluN2B receptors following application of agonists glutamate and glycine (Glu + Gly, 100 μM each) and 1 μM OptoNAM-3 either in the dark (left), or preilluminated at 365 nm (middle) or 350 nm (right). (C) Dose–response curves of OptoNAM-3 activity on GluN1/GluN2B receptors in the dark (black curve, IC_50_ = 0.38 ± 0.03 μM, *n* = 4–21) or preilluminated with 365 nm (violet curve, IC_50_ = 1.7 ± 0.2 μM, *n* = 4–17) or 350 nm (lavender curve, IC_50_ = 4.4 ± 0.6 μM, *n* = 3–13). Theoretical curves of pure *cis-*OptoNAM-3 were calculated either from the 365 nm PSS (18% *trans*/82% *cis*, violet dotted line) or from the 350 nm PSS (9% *trans*/91% *cis*, lavender dotted line). (D) Inhibitions by 1 μM *trans-*OptoNAM-3 of GluN1/GluN2B, GluN1/GluN2A, GluN1/GluN2C, and GluN1/GluN2D diheteromers, as well as GluN1/GluN2A/GluN2B triheteromers (inhibition: 69% ± 2%, *n* = 21 for GluN1/GluN2B; 2.7% ± 0.5%, *n* = 4 for GluN1/GluN2A; 11.4% ± 0.9%, *n* = 4 for GluN1/GluN2A/GluN2B; 3.2% ± 0.5%, *n* = 4 for GluN1/GluN2C; and 2.2% ± 0.2%, *n* = 3 for GluN1/GluN2D). (E) Percentage of inhibition by 1 μM *trans-*OptoNAM-3 in the dark of wt GluN1/GluN2B receptors (in blue) or of receptors mutated at key residues involved in EVT-101 binding only (GluN1/GluN2B-Q110G, in aquamarine), in ifenprodil binding only (GluN1-L135H/GluN2B, in violet), or on a mutant disrupting binding at both EVT-101 and ifenprodil binding sites (GluN1/GluN2B-ΔNTD, in pink; dotted lines above the bar mean that inhibition was calculated from the dose–response curve in Figure S3E). n.s., *p* > 0.05; **, *p* < 0.01; Kruskal–Wallis’ test followed by Dunn’s multiple comparison test.

The activity of OptoNAM-3 was functionally monitored by electrophysiology on *Xenopus* oocytes (see Methods). To assess the light-dependent effect, we tested the activities of the dark PSS (mostly *trans*) and the UV PSS (mostly *cis*, see above) separately. For each concentration, we prepared a solution divided into two samples: one in which the compound was kept in the dark for the duration of the experiment (dark PSS state); and the other that was preilluminated with either 350 or 365 nm light (350 or 365 nm PSS) and then kept away from the light for the duration of the experiment in order to avoid photoisomerization of the compounds by ambient light (see Methods). Due to its high photostability, *cis*-OptoNAM-3 did not show any relaxation to the *trans* state during the several hours of experimentation. We generated dose–response curves of OptoNAM-3 dark and UV PSS on wild-type (wt) GluN1/GluN2B NMDA receptors (Figure 3A) in the presence of saturating concentrations of agonists (100 μM glutamate and glycine). OptoNAM-3 had an IC_50_ of 380 nM (Figure 3B,C) in the dark, which is in the same range of activity as its parent compound **3** (*K*_i_ = 93 nM;^41^Table S1). In addition, OptoNAM-3 displayed a significant photodependent activity, since its IC_50_ for GluN1/GluN2B NMDARs increased by 4.5 and 11.5-fold compared to the dark condition when the solution was preilluminated with 365 and 350 nm light, respectively (IC_50_ = 1.7 μM and 4.4 μM at 365 and 350 nm, respectively; Figure 3B,C and Table S1). To gain further insights into the photodependence of OptoNAM-3 activity, we calculated the theoretical dose–response curve of a pure *cis*-OptoNAM-3 population, knowing that solutions preilluminated with 365 and 350 nm contain, respectively, 18% and 9% of residual *trans*-OptoNAM-3 (Figure 2C) and assuming that the dose–response curve in the dark represents the activity of a pure *trans* population (see Methods). Our calculations show that the *cis* isomer is inactive on GluN1/GluN2B NMDARs and that the residual activity observed after UV illumination entirely results from the activity of the remaining *trans* isomer (Figures 3C and S3A,B). The limiting factor of the photodependence of OptoNAM-3 activity is therefore the yield of *trans*-to-*cis* photoconversion by UV light.

NMDARs exist as multiple subtypes in the brain that are formed by the combination of two GluN1 and either two identical (diheteromers) or different (triheteromers) GluN2 (GluN2A-D) subunits.^1^ We assessed the selectivity of *trans-*OptoNAM-3 (dark PSS) for the other NMDAR subtypes. 1 μM of OptoNAM-3, which induces 69% inhibition of GluN1/GluN2B diheteromeric NMDARs, induced very little (max 3%) inhibition of GluN1/GluN2A, GluN1/GluN2C, and GluN1/GluN2D diheteromeric receptors (Figure 3D). The compound had a slightly stronger effect on GluN1/GluN2A/GluN2B triheteromeric NMDARs (11.4% inhibition, Figure 3D). Like other ifenprodil derivatives, OptoNAM-3 is therefore selective for GluN2B-containing NMDARs with a marked preference for GluN1/GluN2B diheteromers over GluN1/GluN2A/GluN2B triheteromers.^43,44^ We also verified that, up to 10 μM, OptoNAM-3 had no effect on the other classes of ionotropic glutamate receptors (iGluRs) (Figure S3C,D).

We finally investigated the location of the OptoNAM-3 binding-site on the receptor. GluN2B-selective antagonists like ifenprodil are known to bind at the interface between GluN1 and GluN2B N-terminal domain (NTD) upper lobes.^18−20^*Trans-*OptoNAM-3 activity was, like for ifenprodil, drastically reduced in receptors in which the NTD of GluN2B has been deleted^45,46^ (GluN1/GluN2B-ΔNTD receptors, 100-fold shift in IC_50_ between wt and GluN1/GluN2B-ΔNTD receptors, Figures 3E and S3E). In addition, *trans*-OptoNAM-3 IC_50_ was increased in the presence of ifenprodil, which is consistent with a competition between the two compounds (Figure S3F). The binding site for GluN2B-selective antagonists at the GluN1/GluN2B NTD dimer interface contains two partially overlapping pockets that accommodate GluN2B-selective NAMs of distinct chemical scaffolds:^19^ either scaffolds related to ifenprodil or scaffolds related to another GluN2B-selective NAM called EVT-101.^47^ By mutating residues selectively disrupting the binding of the compounds in one or the other pocket,^19^ we show that OptoNAM-3 binds the ifenprodil binding pocket and not the EVT-101 pocket (Figure 3E). We have therefore designed a potent NMDAR NAM, OptoNAM-3, which shares the same binding site and selectivity for GluN2B-containing NMDARs as previous GluN2B-selective antagonists but, in addition, displays a photodependent effect, with the *trans* isomer being the only active form on GluN2B-NMDARs.

### Fast and Reversible Photomodulation of GluN2B-NMDARs in Mammalian Cells

**Figure 4:**
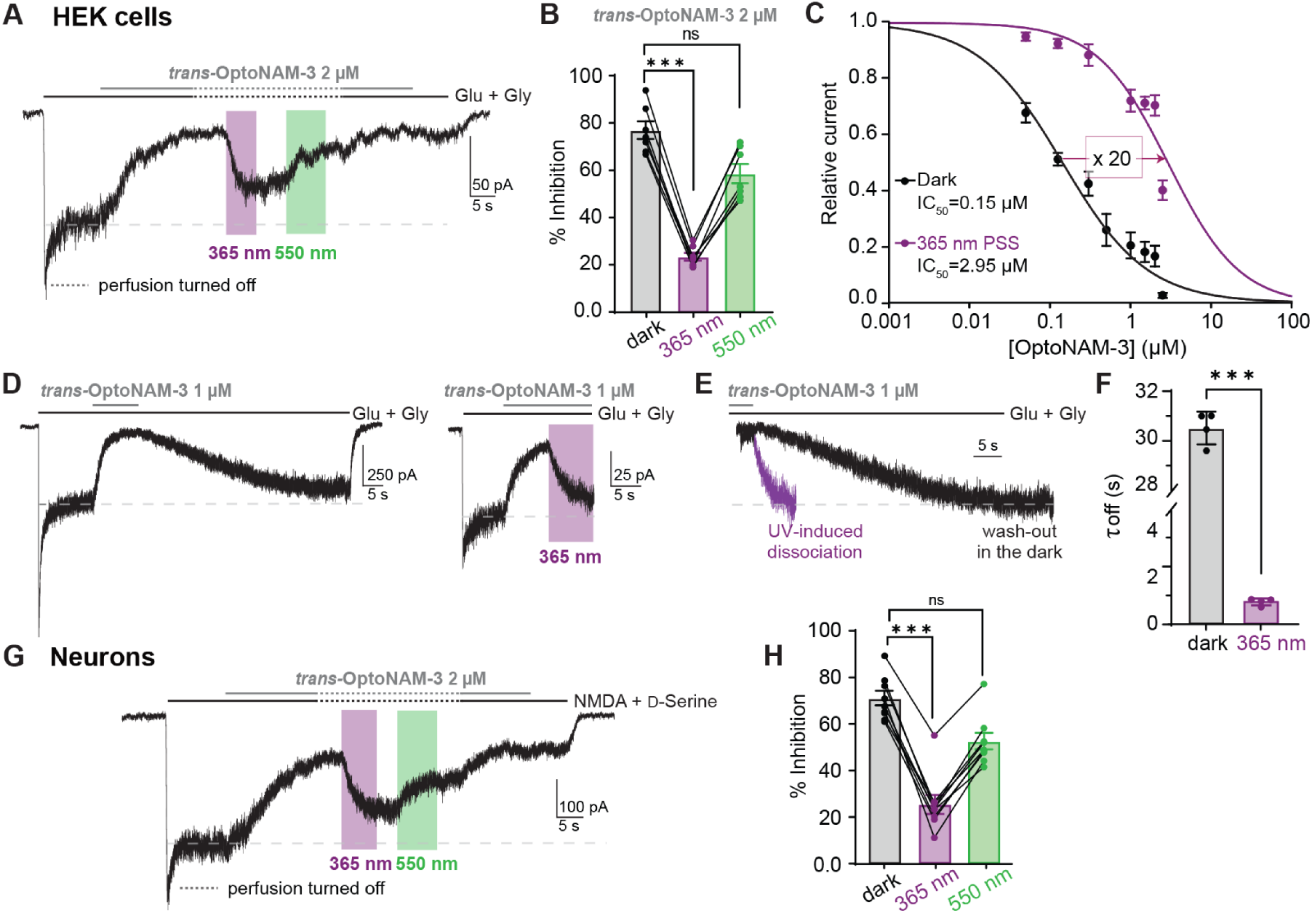
Fast and reversible photomodulation of GluN1/GluN2B receptors in mammalian cells. (A) Current trace from a HEK cell expressing GluN1/GluN2B receptors following application of glutamate and glycine (Glu + Gly, 100 μM each) and 2 μM OptoNAM-3 in the dark (mostly in *trans*). Once steady-state inhibition by *trans*-OptoNAM-3 was reached, perfusion was stopped (dashed lines), and the patched cell and the surrounding extracellular medium were illuminated by 365 nm (violet bar) and then 550 nm light (green bar). (B) OptoNAM-3 inhibition depending on the light conditions. 2 μM OptoNAM-3 inhibits GluN1/GluN2B currents by 77% ± 4% in the dark and by 23% ± 2% under UV light and can be restored to 59% ± 4% by 550 nm light. *n* = 7; n.s., *p* > 0.05; ***, *p* < 0.001; Friedman’s (repeated measures) test followed by Dunn’s multiple comparison test. (C) Dose–response curves of OptoNAM-3 on GluN1/GluN2B expressed in HEK cells in the dark (black curve, IC_50_ = 0.15 ± 0.03 μM, *n* = 4–8) and when the cell is illuminated at 365 nm (violet curve, IC_50_ = 3.0 ± 0.9 μM, *n* = 4–7). (D) Current traces showing the relief of *trans*-OptoNAM-3 inhibition by OptoNAM-3 washout in the dark (left) or by UV light illumination (right). (E) Superposition of the inhibition relief traces in the dark (black) and under UV light (violet) showing much faster relief of inhibition by UV illumination than washout of *trans*-OptoNAM-3. (F) Summary of the kinetics of inhibition relief in the dark and UV conditions (τ_off_ = 30.5 ± 0.5 s, *n* = 4 in the dark and τ_off_ = 0.82 ± 0.06 s, *n* = 4 under UV illumination). ***, *p* < 0.001; Mann–Whitney’s test. (G) Current trace from a DIV 8 cultured cortical neuron following application of NMDA (300 μM) and d-serine (50 μM), and 2 μM OptoNAM-3 in the dark (in *trans*). Same protocol as in Figure 4A. (H) OptoNAM-3 inhibition depending on the light conditions. 2 μM OptoNAM-3 inhibits NMDA currents by 70% ± 3% in the dark and by 25% ± 4% under UV light and can be restored to 52% ± 3% by applying 550 nm light. *n* = 9, n.s., *p* > 0.05, ***, *p* < 0.001; Friedman’s test followed by Dunn’s multiple comparison test.

Now that we have established the photodependence of OptoNAM-3 action, we tested whether this compound could be used to perform real-time modulation of NMDAR activity with light. To answer this question, we turned to mammalian cells, whose transparency allows homogeneous illumination of all membrane-expressed NMDARs. OptoNAM-3 was perfused together with agonists glutamate and glycine on HEK cells expressing GluN1/GluN2B receptors. When applied in the dark, 2 μM OptoNAM-3 induced on average 77% inhibition of GluN1/GluN2B currents (Figure 4A,B). This inhibition was partially abolished by UV (365 nm) illumination (23% remaining inhibition) and partially restored by 550 nm illumination (59% inhibition) (Figure 4A,B). OptoNAM-3 thus allows real-time and reversible inhibition of GluN1/GluN2B activity with light. By plotting the effect of different concentrations of OptoNAM-3 in the dark and during UV illumination, we observed a 20-fold UV-induced shift of OptoNAM-3 IC_50_ compared to the dark condition (Figure 4C). This shift was greater when the compound was preilluminated in solution and then applied onto oocytes (see Figure 3C above; 4.5-fold shift in IC_50_ between the dark and the 365 nm conditions). The stronger photodependence of OptoNAM-3 action on HEK cells might stem either from differences between cellular expression systems (*Xenopus* oocytes vs HEK cells) or from the different irradiation contexts (in solution for experiments in *Xenopus* oocytes and in a cellular context for HEK cells) (see below).

The dissociation rates of compounds acting at the ifenprodil site are usually very slow (in the minute range).^27,28^ This slow dissociation rate is actually an issue in native tissues like brain slices, in which the effects of such compounds become irreversible. Accordingly, the time constant of current recovery from inhibition after washout of *trans*-OptoNAM-3 in the dark was slow (τ_off_ = 30.5 ± 0.5 s, Figure 4D–F). Relief of OptoNAM-3 inhibition by UV light, on the other hand, was much faster with a time constant in the subsecond time-range (τ_off_ = 0.82 ± 0.06 s, Figure 4D–F).

We then turned to native NMDA currents. We repeated these experiments on cultured cortical neurons at days in vitro [DIV] 6–8, a stage at which GluN2B-containing NMDARs form the major population of neuronal NMDARs.^48^ On cortical neurons, 2 μM OptoNAM-3 induced 70% inhibition of NMDA-induced currents in the dark. The lower inhibitory effect of *trans-*OptoNAM-3 on neurons compared to HEK cells is most likely due to the mixture of NMDAR subtypes expressed in neurons.^48^ Similar to HEK cells, inhibition by OptoNAM-3 was decreased to 25% under UV light illumination and partially restored to 52% by 550 nm light (Figure 4G,H). Hence, with its strong photodependence of action and its fast kinetics of photomodulation, OptoNAM-3 allows fast relief of inhibition of native GluN1/GluN2B receptors, something that is not possible with regular GluN2B-selective NAMs. This effect is furthermore partially reversible thanks to the use of 550 nm light.

### OptoNAM-3 Acts as an In Situ Red-Shifted Photodependent Antagonist

Given the slow dissociation rate of OptoNAM-3 in its *trans* configuration (dark condition), we hypothesized that the fast relief of inhibition observed upon UV-light illumination resulted from *trans*-to-*cis* interconversion of OptoNAM-3 inside its binding site. The UV-induced relief of inhibition might then reflect either dissociation of *cis*-OptoNAM-3 from the binding site or the isomerization rate from the active *trans* to the inactive *cis* with the *cis* remaining in the binding site (silent modulator). To further investigate the mechanisms by which this compound exerts its photodependent biological activity, we studied the spectral dependence of OptoNAM-3 photoisomerization in solution (referred to “free OptoNAM-3” below) and in a cellular context (referred to “bound OptoNAM-3” below). Azobenzenes indeed exhibit strong electronic absorption of their conjugated pi system, and their absorption spectra can be altered when they aggregate, are complexed, or simply dwell in a different solvent.^49,50^ We therefore investigated if the photochemical properties of bound OptoNAM-3, which is confined in its binding site and exerts multiple nonbonding interactions with it, differ from the ones of free OptoNAM-3.

**Figure 5:**
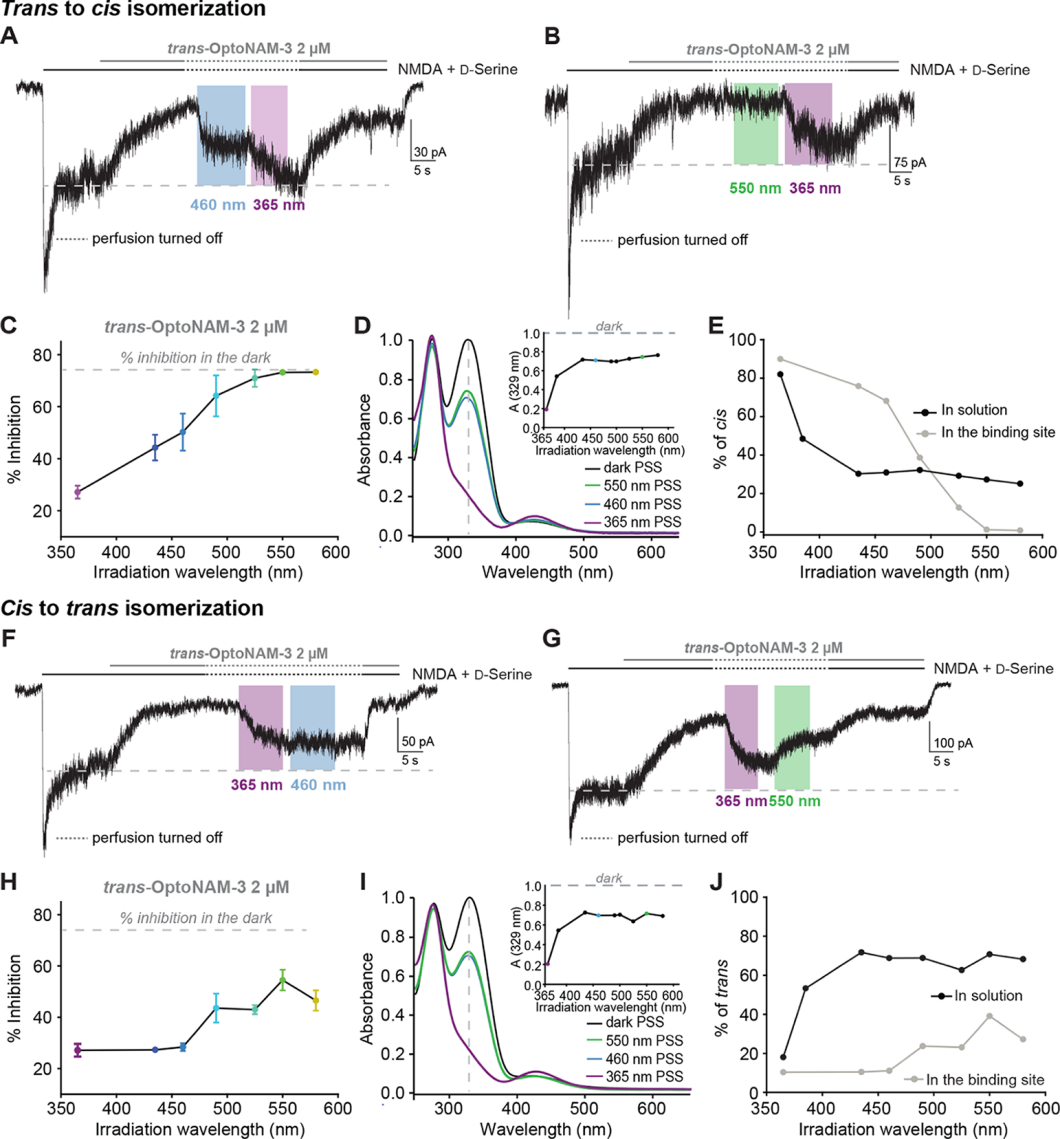
Binding of OptoNAM-3 onto GluN1/GluN2B NMDARs changes its photochemical properties. (A–E) Spectral dependence of *trans*-to-*cis* isomerization (A, B) Current traces from DIV 6–8 cultured cortical neurons following application of NMDA (300 μM) and d-serine (50 μM), as well as 2 μM OptoNAM-3. As described in Figure 4, once steady-state inhibition by *trans*-OptoNAM-3 was reached, the patched cell and the surrounding extracellular medium were illuminated by various wavelengths: in our example, 460 nm (blue bar) in Figure 5A and 550 nm (green bar) in Figure 5B followed by 365 nm (violet bar) as a control for the lowest amount of inhibition. (C) Percentage of inhibition by 2 μM OptoNAM-3 upon irradiation with different wavelengths on the cell. 365 nm was the most efficient wavelength allowing reduction of inhibition to 27% ± 2%. *n* = 23 for 365 nm; *n* = 4 for 435 nm; *n* = 5 for 490 and 550 nm; and *n* = 6 for 460 and 525 nm. Data points from 435 to 580 nm represented in this figure were scaled between 365 nm and dark condition for better accuracy (see Methods for the calculation protocol). (D) UV–visible spectra of a solution of OptoNAM-3 in the dark (black curve) and after illumination with 365, 460, or 550 nm (violet, blue, and green curves, respectively, extracted from Figure S1M). Inset: absorbance at the peak absorbance wavelength of the dark state (329 nm) of OptoNAM-3 following illumination at various wavelengths of the dark PSS. Note that in solution, the 460 and 550 nm PSS are similar. (E) Proportion of the *cis* isomer in OptoNAM-3 PSS for different illumination wavelengths in solution (calculated from the UV–visible spectra as in Figure 5D, black points) or in a cellular context (cAM-3 inhibition under various wavelengths as shown in Figure 5A–C, gray points, see Methods). This graph reveals a red shifting of OptoNAM-3 spectral properties in a cellular context. (F–J) Spectral dependence of *cis*-to-*trans* isomerization. (F,G) Current traces from cortical neurons following application of NMDA (300 μM) and d-serine (50 μM), as well as 2 μM OptoNAM-3. Once steady-state inhibition by *trans*-OptoNAM-3 was reached (dark), the patched cell and the surrounding extracellular medium were illuminated by 365 nm light to convert OptoNAM-3 to a mostly *cis* configuration. Then, various wavelengths were applied to convert OptoNAM-3 back to *trans*: in our examples, 460 nm (blue bar) in Figure 5F and 550 nm (green bar) in Figure 5G. (H) Percentage of inhibition by 2 μM OptoNAM-3 upon irradiation with different wavelengths post 365 nm illumination of the cell. 550 nm is the most efficient wavelength allowing recovery of inhibition to 55% ± 4%. *n* = 23 for 365 nm; *n* = 4 for 435, 460, 490, and 525 nm; *n* = 9 for 550 nm; and *n* = 5 for 580 nm. Data points from 435 and 580 nm represented in this figure were scaled between 365 nm and dark conditions (see Methods for the calculation protocol). (I) UV–visible spectra of a solution of OptoNAM-3 in the dark (black curve), after 365 nm illumination (violet curve), and subsequent illumination with 460 (blue curve) or 550 nm (green curve) (extracted from Figure S1N). Inset: absorbance at the peak absorbance wavelength of the dark state (329 nm) of OptoNAM-3 following illumination at various wavelengths of the 365 nm PSS. (J) Proportion of the *trans* isomer in OptoNAM-3 PSS obtained upon irradiation with various wavelengths of the OptoNAM-3 365 nm PSS either in solution or in a cellular context (same calculation methods as in Figure 5E). This graph reveals a red-shifted and less efficient *cis*-to-*trans* photoconversion in a cellular context (OptoNAM-3 likely in its binding site) than in solution.

We measured the degree of OptoNAM-3 photoisomerization when illumination was performed either on cultured neurons pre-equilibrated with OptoNAM-3 (bound OptoNAM-3, Figure 5A–C,F–H) or in solution (free OptoNAM-3, Figures 2B, S1M,N, and 5D,I). We first analyzed OptoNAM-3 *trans*-to-*cis* isomerization (Figure 5A–E). To this aim, OptoNAM-3 in the dark was irradiated with light of various wavelengths. The degree of photoisomerization of bound OptoNAM-3 was calculated from the percentage of NMDA current inhibition induced by OptoNAM-3 under the different wavelength conditions (Figure 5A–C,E and see Methods). The degree of free OptoNAM-3 photoisomerization was calculated by UV–visible spectroscopy, by measuring the absorbance of the irradiated solution at the *trans*-OptoNAM-3 peak absorption wavelength (Figure 5D,E and see Methods). We observed that, on cortical neurons, wavelengths up to 460 nm allowed considerable inhibition relief compared to the dark condition, showing an efficient *trans*-to-*cis* transition with both UV and blue light (52% inhibition under 460 nm light, corresponding to ∼68% of *cis*-OptoNAM-3; Figure 5A,C,E). On the contrary, blue light (435 and 460 nm) illuminations allowed only little *trans*-to-*cis* isomerization in solution (∼31% of *cis* after 460 nm illumination, Figure 5D,E). Overall, we observed a red-shift in the action spectrum of OptoNAM-3 in the binding site compared to that in solution (Figure 5E): wavelengths up to 460 nm allowed a good *trans*-to-*cis* photoconversion of bound OptoNAM-3, while for free OptoNAM-3, *trans*-to-*cis* conversion was unfavored for wavelengths superior to 380 nm. Interestingly, we also calculated a better *trans*-to-*cis* isomerization of bound OptoNAM-3 by 365 nm light (∼90% *cis* for bound OptoNAM-3 365 nm PSS vs 82% *cis* for free OptoNAM-3 365 nm PSS, Figure 5E), which is consistent with the better separation of OptoNAM-3 activity between the dark and 365 nm conditions when the compound was directly irradiated on the cell (Figure 4C) than when it was preirradiated in solution (Figure 3C). On the other hand, wavelengths of 550 and 580 nm did not induce visible *trans*-to-*cis* isomerization in the binding site, while they induced a significant photoconversion in solution (∼30% of *cis* after illumination) (Figure 5B,E and see Figure 2).

We then analyzed the spectral properties of the reverse reaction: OptoNAM-3 *cis*-to-*trans* isomerization. To this aim, OptoNAM-3 365 nm PSS was irradiated with lights of various wavelengths to determine the degree of return to the *trans* state. Similar to the *trans*-to-*cis* conversion, we observed altered spectral properties of the compound when bound to the receptor. Indeed, wavelengths like 435 and 460 nm did not allow *cis*-to-*trans* isomerization of “bound” *cis*-OptoNAM-3, as evidenced by the similar degrees of OptoNAM-3 inhibition at 365, 435, and 460 nm (27% inhibition, corresponding to ∼10% *trans* isomer; Figure 5F,H,J). On the contrary, these wavelengths allowed significant return to the *trans* state in solution (72 and 69% of the *trans* isomer, respectively; Figure 5I,J). In addition, 550 and 580 nm did not allow as good as a return to the *trans* state for bound OptoNAM-3 than for free OptoNAM-3 (Figure 5G,H,J).

When photoswitched in a cellular context, OptoNAM-3 therefore displays red-shifted properties that likely stem from the compound interaction with the NMDAR protein environment. This feature is interesting, since it means that wavelengths in the visible range like 435 or 460 nm may be used in vivo as biocompatible wavelengths to convert the *trans* isomer to *cis* and reduce the harmfulness of high energy wavelengths such as 365 nm.

### OptoNAM-3 Controls GluN2B-Dependent Pathological Processes and Animal Behavior in a Red-Shifted, Photodependent Manner

We first investigated whether OptoNAM-3 could be used in the context of a patho-physiological process related to GluN1/GluN2B receptors. Overactivation of NMDARs induced by tonic exposure to glutamate or NMDA is well-known to trigger neuronal death.^6^ GluN1/GluN2B receptors are thought to be the major player of this neurotoxicity, and GluN1/GluN2B antagonists have proven to be potent neuroprotectants in vitro and in vivo.^5,15^ To test the neuroprotective activity of OptoNAM-3 and its photodependence of action, we performed a protocol of excitotoxicity based on exposure to NMDA (100 μM) of cultured cortical neurons at DIV 14,^15^ in the presence of OptoNAM-3. The cell culture was either kept in the dark (OptoNAM-3 in *trans,* active isomer) or illuminated by 365 nm light (converting OptoNAM-3 in mostly *cis*, inactive isomer). OptoNAM-3 in the dark, at a concentration of 5 μM, induced, like ifenprodil, an ∼50% increase in cell survival (Figure S4). This increase in survival was significantly smaller when UV was applied after the addition of OptoNAM-3 (∼35% survival in the OptoNAM-3 + UV condition, Figure S4). No photodependent effect was observed on neurons treated with ifenprodil. OptoNAM-3 is therefore able to promote cell survival in neuron cultures in a photodependent manner.

We finally demonstrated that OptoNAM-3 allows reversible control of animal behavior with light. *Xenopus laevis* tadpoles can be used as a simple vertebrate model to validate the activity of photoswitchable drugs applied to neurobiology. Indeed, those tadpoles are transparent allowing good light penetration. In addition, the neuronal pathways underlining the aspects of their swimming behavior have been well characterized.^51^ In particular, NMDA receptors were shown to play a role in their locomotion pattern.^52,53^ Moreover, the ifenprodil binding site is conserved in this species^54^ and ifenprodil inhibits *Xenopus* and mammalian GluN1/GluN2B receptors with similar affinity.^55^*Xenopus laevis* tadpole locomotion is therefore a good behavioral model to test the in vivo effects of GluN2B-selective antagonists. We thus tested whether OptoNAM-3 could photomodulate the activity of free-swimming tadpoles.

**Figure 6:**
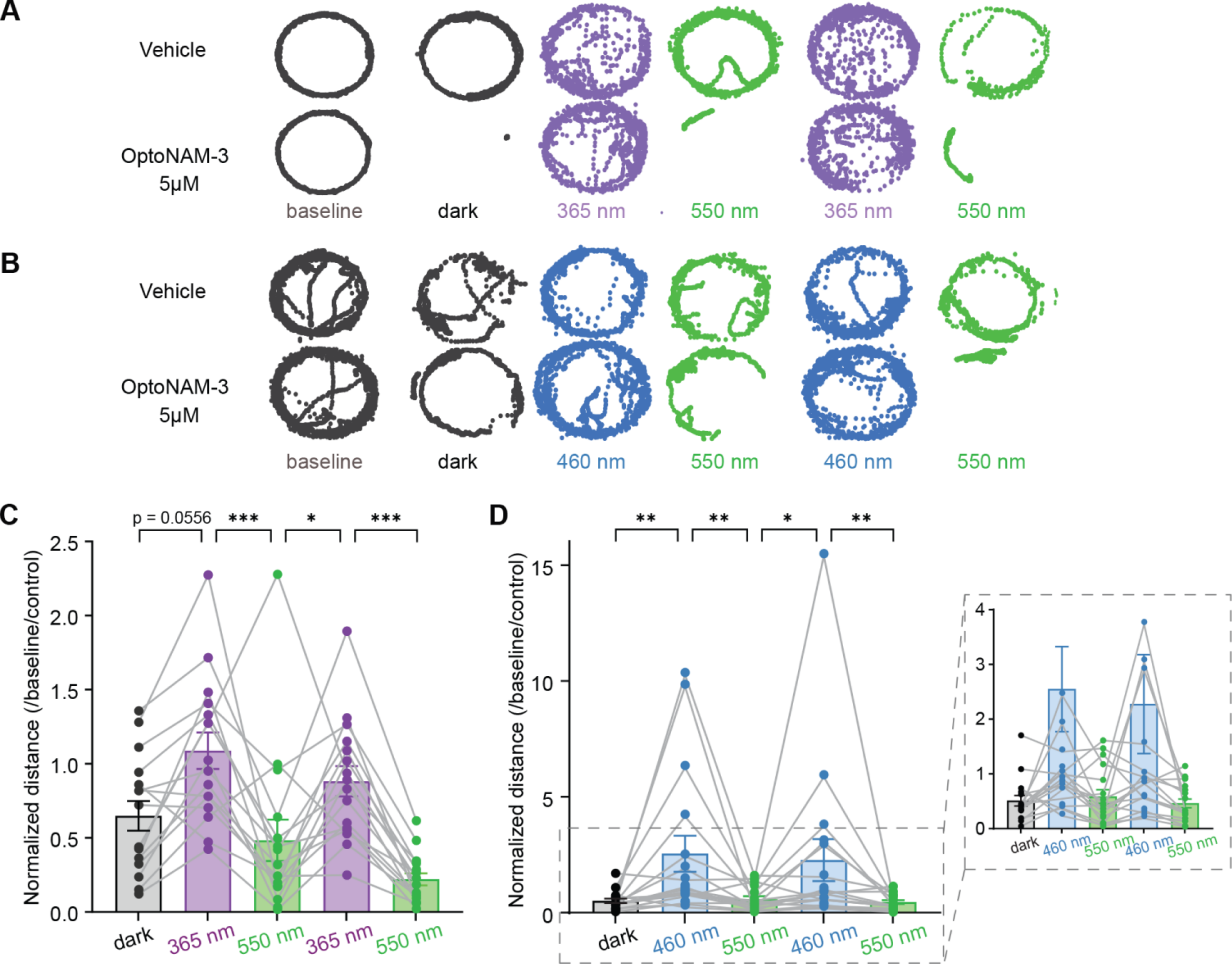
OptoNAM-3 photomodulates *Xenopus* tadpole locomotion in vivo. (A, B) Positions (represented by dots) of a tadpole’s stomach in a well, taken every 40 ms during 3 min of recording for baseline and dark conditions, and 1 min of recording for either 365 and 550 nm (A), or 460 and 550 nm illumination cycles (B). Top: tadpole incubated in vehicle (0.1% DMSO, control condition). Bottom: tadpole incubated in 5 μM OptoNAM-3. (C, D) Normalized distance (by their baseline locomotion in the dark and the locomotion of the control group in the same light condition, see Methods and Figure S5) traveled by tadpoles (1 point represents the mean traveled distance per well containing 3 tadpoles) exposed to OptoNAM-3 in the dark and during UV/green (C) or blue/green light cycles (D). *n* = 16 for Figure 6C and *n* = 17 for Figure 6D; 48 and 51 tadpoles in total, respectively. The means of 3 tadpoles per well were used to perform a paired statistical test. n.s., *p* > 0.05; ***, *p* < 0.001; **, *p* < 0.01; *, *p* < 0.05; Friedman’s test followed by Dunn’s multiple comparison test.

In our behavioral assay, tadpoles were placed in groups of three animals per well of a 12-well plate allowing them to swim freely. Their baseline locomotion was recorded for 3 min in the dark, after which they were incubated for 45 min in a solution containing either 0.1% DMSO (control) or 5 μM OptoNAM-3 (refer to Methods and Figure S5A for details). For each condition (control and OptoNAM-3), tadpole locomotion was recorded for 3 min in the dark, followed by cycles of 1 min illumination at 365 nm and then 550 nm, and interspersed with 3 min rest periods to avoid bias due to a poststimulation refractory period^51,56^ (Figure S5A). We also conducted the same protocol using 460/550 nm light cycles, as OptoNAM-3 can switch from the *trans* to *cis* state upon exposure to blue light at 460 nm when pre-equilibrated on cells (see above). The tadpole traveled distance was calculated from precise position coordinates of tadpole body parts across frames, assessed by automatic position multianimal tracking using the open-source program DeepLabCut.^57,58^*Xenopus laevis* tadpoles present nonvisual photoreceptors that generate a photomotor response characterized by an increase of activity upon UV light illumination.^59^ We indeed observed an increase in tadpole locomotion with UV and blue light compared to 550 nm light in the control condition (∼1.5-fold, Figures 6A,B and S5B,C, gray points). The photodependent effect of OptoNAM-3 on locomotion was therefore quantified by calculating the traveled distance normalized twice: by the tadpole baseline locomotion (to decrease interindividual variability linked to the locomotion speed) and, for each condition, by the locomotion of the control group (to remove the nonspecific effect of UV or blue light). After incubation with OptoNAM-3, tadpoles from the UV/visible and blue/green cycle protocols (Figure 6A,B,D, respectively) exhibited in the dark a 35% and 50% reduction in locomotor activity, respectively, in comparison to the baseline condition (Figure 6C,D and Movies S1 and S2). Their locomotion could be restored to baseline activity with UV or blue light and inhibited again by 550 nm light for two cycles (Figure 6C,D and Movies S1 and S2). Consequently, OptoNAM-3 enabled fast and reversible control of tadpole locomotion using light. In addition, the compound red-shifted properties observed in vitro were conserved in vivo. OptoNAM-3 therefore holds great promise to photocontrol native GluN2B-NMDARs with the high biocompatibility of red-shifted photoswitches.^29,31^

### Origin of the Red-Shifted Properties of Bound OptoNAM-3

We wondered about the origin of this drastic shift in OptoNAM-3 spectral properties when the compound is bound to its binding site. We first measured OptoNAM-3 UV–visible spectra and its photoconversion properties in solvents with lower relative permittivity (DMSO: ε = 46.7 and toluene: ε = 2.38) in a hope to mimic a more hydrophobic environment within the protein (compared to water, ε = 80.1). For the dark PSS (*trans*-OptoNAM-3), we observed no shift in the peak corresponding to the π → π* transition (maximum around 330 nm for all solvents, Figure S6) but a 20 nm shift to the right of the *n* → π* transition (from 421 nm in aqueous solvent to above 440 nm in DMSO and toluene, Figure S6). In addition, we observed a slightly more efficient *trans*-to-*cis* isomerization by 365 nm light in DMSO compared to aqueous solution (365 nm PSS: 90% *cis* in DMSO, PSS measured by ^1^H NMR, Spectrum S3K, vs 82% *cis* in physiological aqueous buffer, Figure 2C). The larger proportion of the *cis* isomer in the 365 nm PSS in DMSO might account for the larger separation between the dark and 365 nm dose–response curves for bound-OptoNAM-3 (Figure 4C) than for free OptoNAM-3 (Figure 3D). However, contrary to bound OptoNAM-3, in both DMSO and toluene, the 460 and 550 nm PSS were similar (as assessed by superimposable UV–visible spectra, Figure S6). Therefore, these more hydrophobic solvents cannot fully recapitulate the spectral properties of bound OptoNAM-3.

**Figure 7:**
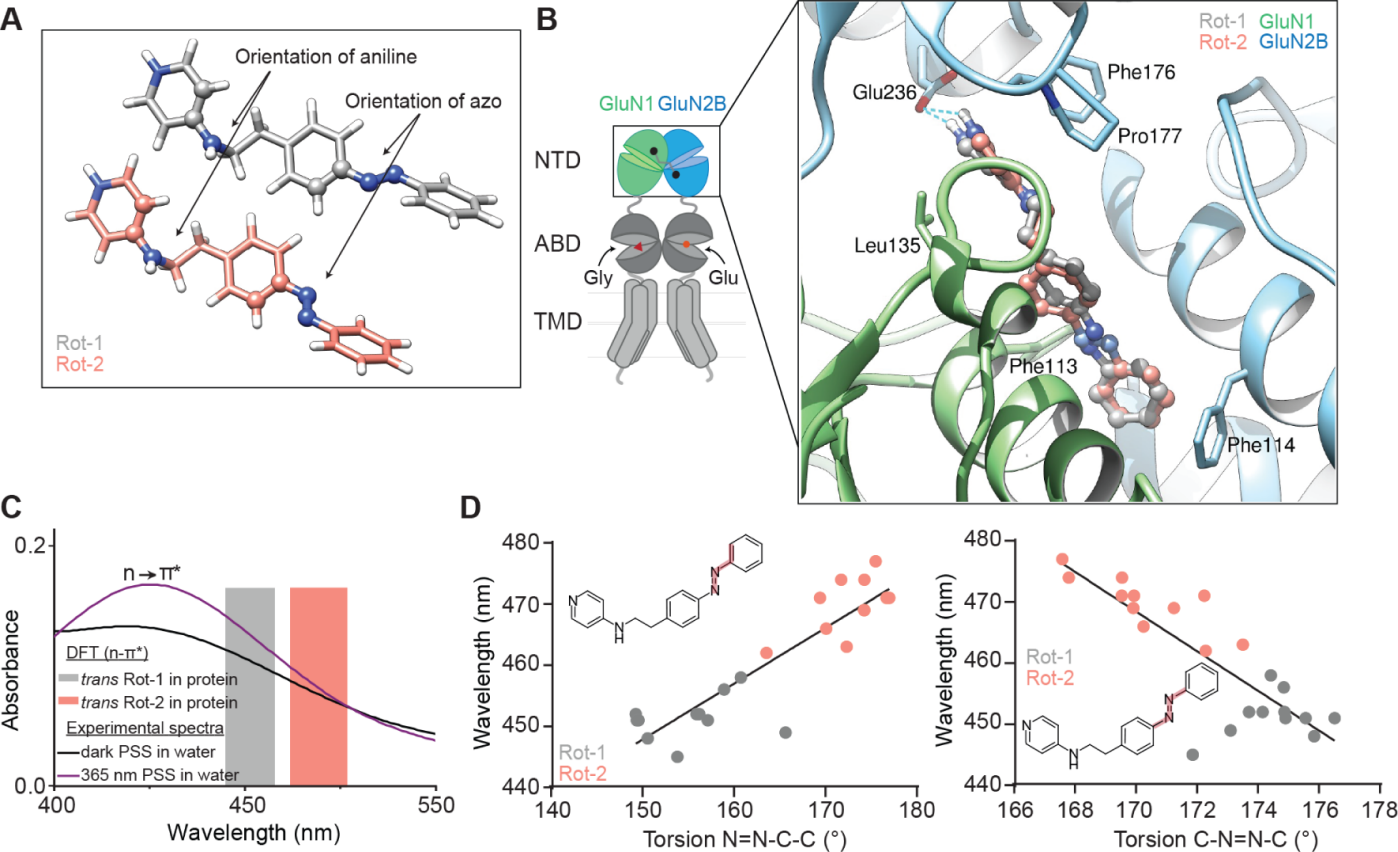
Molecular simulations of OptoNAM-3 in solution and in its binding site. (A) 3D representation of *trans*-OptoNAM-3 Rot-1 (gray) and Rot-2 (salmon) and definition of the two dihedral angles (displayed as balls and sticks) that were monitored during molecular dynamics simulations. (B) Docking poses of *trans*-OptoNAM-3 Rot-1 (gray) and Rot-2 (salmon) in their binding site: like ifenprodil, at the interface between GluN1 (green) and GluN2B (blue) NTD upper lobes. Residues important for ifenprodil binding^18−20^ are highlighted as sticks. Note that these residues also contact OptoNAM-3 showing a similar binding mode between the two compounds (see also Figure S9A). (C) Superposition of the experimental *n* → π* band of the UV–vis spectra of OptoNAM-3 dark PSS (dark line) and 365 nm PSS (violet line) in aqueous buffer (Ringer pH 7.3) to the theoretical *n* → π* transitions of bound *trans*-OptoNAM-3 Rot-1 and Rot-2 predicted by DFT calculations (gray and salmon bars representing respectively the range of computed wavelengths for *trans-*OptoNAM-3 Rot-1 and Rot-2 across the different snapshots of the dynamic, see Table S3). (D) Relationships between the N=N—C—C and C—N=N—C torsion angles (as highlighted in pale red in the inset chemical structures) of bound *trans*-OptoNAM-3 in the 11 snapshots selected for DFT calculations and their computed *n* → π* absorption wavelengths, for Rot-1 (in gray) and Rot-2 (in salmon). Linear regression: *R*^2^ = 0.803 (left) and 0.705 (right).

We therefore turned to molecular dynamics (MD) simulations and density functional theory (DFT) calculations to get insights into the spectral properties of OptoNAM-3 bound to its binding site. After docking *trans*-OptoNAM-3 in the active site based on the ifenprodil position in structure 5EWJ,^19^ we observed during MD simulations that both the ligand and the protein were quite flexible (see details in Text S2). Indeed, both the aniline and C—C—N=N angles from the ligand could interconvert between 0° and 180° (Figure S7A), and the distance between GluN1 and GluN2B NTD lower lobes from the protein significantly increased during the simulation. We thus performed two additional simulations where the protein heavy atoms were restrained close to their crystallographic positions; one simulation started from the previous initial conformation of OptoNAM-3 (a C—C—N=N angle of 141°, called Rot-1; Figure 7A), whereas the other one started from a conformation where the C—C—N=N dihedral angle was rotated by 180° (an angle of −39°, Rot-2 rotamer; Figure 7A). The restraints rigidified both the protein and the ligand, since no change of aniline orientation or of the C—C—N=N angle was observed, and Rot-1 and Rot-2 stayed in their basins (Figures 7A,B and S7B). In addition, a simulation of *trans*-OptoNAM-3 alone in water solution was performed, and we observed numerous transitions between the different conformations (Figure S7C).

Eleven snapshots of free *trans*-OptoNAM-3 in solution were extracted, on which we computed vertical energies and oscillator strengths (f) using quantum mechanics (QM) and an implicit description of the solvent. Our results are in a good agreement with experimental data (mean deviation of 0.095 eV for the π → π* transition, see Figure S8A and Table S2), which validates our computational procedure. Then, 11 snapshots of the protein–ligand complexes were extracted, and the vertical excitations were investigated (Table S3) by quantum mechanics/molecular mechanics (QM/MM): we found that the π → π* transitions of both rotamers of bound OptoNAM-3 were blue-shifted by about 0.26 eV (23 nm) compared to free OptoNAM-3 with no significant change of the oscillator strength. However, a difference arose for the *n* → π* excitation energy since the one of Rot-2 was 0.11 eV (20 nm) red-shifted with respect to the excitation energy of either Rot-1 or free OptoNAM-3 (∼470 nm vs ∼450–455 nm, respectively, Tables S2 and S3 and Figure 7C). Moreover, we also observed a strong difference in the computed oscillator strengths (f) of the *n* → π* transition, since the one of Rot-2 was in average 10-fold higher than the one of Rot-1 or free OptoNAM-3 (Tables S2 and S3). This dimensionless number can be defined as the probability of absorption in a defined transition, meaning that the probability of *trans*-to-*cis* photoconversion is 10-fold higher for bound OptoNAM-3 with the Rot-2 conformation than for free OptoNAM-3. The origin of the difference was investigated by recomputing the *n* → π* vertical transition energies in the absence of the protein but keeping the ligand conformation intact: this did not affect the transition energies nor the oscillator strengths (Table S4, lines 1 and 2). However, the removal of the protein followed by a reoptimization of the compound geometries in vacuum induced (i) a 11 nm red-shift of Rot-1 transition and a slight, 5 nm blue shift of Rot-2 transition, so that the two isomers had similar *n* → π* transition energies (Table S4, line 3); and (ii) a strong decrease of the oscillator strength of the *n* → π* transition of Rot-2, which became similar to the values observed in Rot-1 and free OptoNAM-3. Thus, the spectral properties of bound OptoNAM-3 result from geometrical constraints imposed by the protein rather than from the protein electrostatic environment. Indeed, a strong correlation between the orientation of the azo moiety (N=N—C—C angle) in Rot-1 and Rot-2 snapshots, and their computed *n* → π* absorption wavelengths was found (Figure 7D, left). A similar correlation was observed for the azo bond torsion angle (C—N=N—C angle) and the calculated absorption wavelengths (Figure 7D, right).

Therefore, the red-shift in the *trans*-to-*cis* transition of bound-OptoNAM-3 observed experimentally is likely linked to geometrical constraints imposed by the protein environment on a specific rotamer of OptoNAM-3, Rot-2. Given the much stronger oscillator strength of Rot-2 *n* → π* transition compared to Rot-1, we indeed expect Rot-2 to dominate the spectral properties of bound OptoNAM-3. The special spectral properties of Rot-2 may arise in part from a “flatter” conformation of the azobenzene moiety, as well as a more twisted azo bond compared to Rot-1.

## Discussion

Antagonists selective for GluN2B-NMDARs are routinely used to investigate the GluN2B-NMDAR function, but their slow rate of action is a strong limiting factor for their use in native tissues. To improve their temporal resolution of action, we designed a photoswitchable, GluN2B-selective NAM, OptoNAM-3, whose activity could be reversibly turned on and off by light. OptoNAM-3 combined good photochemical properties in solution with efficient *trans*-to-*cis* and *cis*-to-*trans* conversions by UV and blue–green light, respectively; a very strong photostability of its *cis* inactive isomer (>24 h); and no signs of bleaching or degradation after 10 cycles of illumination. In the dark (*trans* isomer), OptoNAM-3 combined all the pharmacological properties of GluN2B-selective NAMs: a submicromolar potency (IC_50_ of 150–380 nM) in the same range as the potencies of ifenprodil and the OptoNAM-3 parent compound;^27,41,46^ a strong selectivity for GluN2B-NMDARs over other NMDAR subtypes and other iGluR classes, and the same binding site as ifenprodil derivatives. Inhibition by OptoNAM-3 could be relieved by UV light with a large separation of activity (up to 20-fold) between its dark and UV PSS, which lie in the same range as previously published optopharmacological agents.^37,60,61^ We showed that the OptoNAM-3 *trans* isomer is the only active isomer (the *cis* isomer has no inhibitory activity). The amplitude of OptoNAM-3 photodependence of effect is thus solely limited by the photochemical properties of the compound (i.e., the proportion of *trans* remaining after UV illumination). Incomplete yields of light-induced *trans*-to-*cis* and *cis*-to-*trans* transitions are a fundamental limitation of the use of azobenzene photoswitches.^29,62,63^ In our system, we calculated that, to achieve complete disinhibition of GluN2B-NMDARs from a saturating concentration of dark-adapted OptoNAM-3, conversion from *trans* to *cis* should be at least 99.5% (0.5% remaining *trans*, corresponding to a ∼200-fold shift in IC_50_ compared to the dark condition). This amount of photoconversion is difficult to achieve given that azobenzene photostationary states are usually at most 95:5.^29^

In experiments on mammalian cells (HEK cells and mouse cortical neurons), in which *trans*-OptoNAM-3 was first equilibrated in the cell membrane and inside the GluN1/GluN2B receptor before being irradiated with 365 nm light, we observed a stronger separation of activities between the dark and UV conditions (∼20-fold shift in IC_50_) than when the compound was first UV irradiated and then applied onto the cell (4.5-fold shift in IC_50_). In addition, we observed a large red shift in the spectral properties of the *trans*-to-*cis* and *cis*-to-*trans* transitions of bound OptoNAM-3 compared to free OptoNAM-3. Accordingly, 460 nm light was almost as effective as 365 nm light in relieving *trans*-OptoNAM-3 inhibition, whereas this wavelength was ineffective in promoting *trans*-to-*cis* conversion in solution. This red-shifting of OptoNAM-3 properties and the faster kinetics of receptor inhibition relief under UV light than in the dark (τ ∼ 1 s under UV light vs τ ∼ 30 s in the dark) suggest that, when OptoNAM-3 is first equilibrated onto the cell before illumination, *trans*-to-*cis* transition occurs within the binding site. This was previously proposed for photoswitchable ligands targeting mGlu receptors,^62,64^ AMPA receptors,^65^ and the orthosteric glutamate site of NMDARs.^66^ It remains unclear, however, whether the formed *cis*-OptoNAM-3 dissociates from its binding site or remains there as a silent modulator.

These changes of OptoNAM-3 spectral properties could not be fully mimicked by changing the polarity of the medium, although we observed a 20 nm red-shift of the *n* → π* transition with less polar solvents and a more efficient *trans*-to-*cis* isomerization by 365 nm light in DMSO. Molecular dynamics simulations showed that *trans*-OptoNAM-3 could bind the GluN1/GluN2B NTD dimer interface as two distinct rotamers differing in the orientation of the distal phenylazo moiety (Rot-1 and Rot-2, Figure 7A). DFT calculations of the electronic transitions of these two OptoNAM-3 rotamers within their binding site revealed that the *n* → π* transition of the Rot-2 isomer was shifted by 20 nm toward higher wavelengths and its probability of absorption increased by 10-fold compared to OptoNAM-3 in water. We showed that these two properties were controlled by specific geometrical constraints imposed by the binding site, in particular, a more planar conformation of the terminal phenylazo, rather than the protein environment (Table S4). Our findings are consistent with previous experiments and simulations showing an increase in *n* → π* transition energies (and hence a blue-shift in the *n* → π* absorption bands) as the phenylazo moieties rotate away from planarity.^67,68^ In *ortho*-substituted azobenzenes in particular, because of electronic repulsions between the nitrogen lone pairs and the *ortho*-substituents, compounds retaining a planar structure display a red-shift in their *n* → π* transition compared with compounds with twisted phenylazo groups.^67−71^ We can thus assume a similar explanation for the origin of the red-shift in bound Rot-2 spectral properties compared to Rot-1. Assuming the absorption spectrum of *cis*-OptoNAM-3 remains unchanged (if the *cis* isomer quickly dissociates from the receptor), absorption of the Rot-2 isomer of *trans*-OptoNAM-3 in the blue range (linked to the *n* → π* transition) is expected to become stronger than the absorption of *cis*-OptoNAM-3, thus favoring conversion to the *cis* isomer upon irradiation with blue light. This red-shifted and stronger absorbance of the *trans* isomer (compared to the *cis* isomer) in the range of the *n* → π* transition band would also hinder the reverse *cis*-to-*trans* conversion at higher wavelengths, which might explain the lower proportion of the *trans* isomer in the 550 nm PSS of bound-OptoNAM-3.

Differences in photochemical properties between free and bound ligands are not well documented in the literature. Such drastic red-shifting of spectral properties has however been previously observed for a photoswitchable AMPA receptor competitive antagonist, ShuBQX-3.^65^ The authors attributed this shift to the interaction of the ShuBQX-3 azobenzene moiety with an arginine in the inhibitor binding site.^65^ In their case, interaction of ShuBQX with free arginine in solution induced a 75 nm red-shift of ShuBX-3 π → π* transition with no major impact on the *n* → π* transition, suggesting a different mechanism from ours. Given the strong dependence of the photochemical properties of azobenzenes on the molecule geometry and chemical environment, it is highly probable that alterations of the photoswitching properties of photosensitive compounds when bound to their target are more common than currently thought.

OptoNAM-3 allowed fast and partially reversible control of the function of native GluN2B-NMDARs with a high temporal resolution in the second time range in vitro, surpassing the limitations of classical GluN2B antagonists that display slow dissociation kinetics. In addition, thanks to its red-shifted properties, OptoNAM-3 activity could be suppressed by both UV and blue light, which holds great promise for in vivo studies. Indeed, we showed that OptoNAM-3 allowed reversible control of the behavior of freely moving *Xenopus* tadpoles. In the dark, OptoNAM-3 inhibited the locomotion of *Xenopus* tadpoles, consistently with the involvement of NMDARs in this process.^52,53^ Locomotion could be restored to control levels by either application of UV (at 365 nm) or blue light (at 460 nm), and inhibited again by application of green light (550 nm) for 2 cycles. Our in vitro and in vivo experiments therefore establish OptoNAM-3 as a potent pharmacological tool to reversibly control the NMDAR function and animal behavior with strong molecular specificity and high temporal resolution, thanks to the use of visible light. Like most freely diffusible compounds,^36^ OptoNAM-3 is a “light-off” compound (i.e., active in the dark and inactivated by light), which limits its use for systemic injection in rodents. However, like its nonphotosensitive analogs, OptoNAM-3 could potentially be stereotaxically injected in a specific brain area at a given time point. Then, inhibition by OptoNAM-3 could be relieved by UV or blue light to restore endogenous GluN2B-NMDAR function and “normal” animal behavior. Finally, inhibition could be (at least partially) restored using green light without having to resupply the compound, as shown for the control of the locomotion of the *Xenopus* tadpoles. OptoNAM-3 therefore constitutes a great improvement compared to its nonphotosensitive analogs, since it allows inhibition of GluN2B-NMDARs within defined time windows. In the future, antagonists based on diazocines,^72,73^ which are stable in their inactive *cis*-configuration in the dark and converted to their *trans*-active configuration by UV light, could be developed to combine compound systemic injection, as well as fast blocking and unblocking of GluN2B-NMDARs in a specific brain area.

A large number of soluble, photoswitchable ligands have been currently developed for many different targets including receptors, ion channels, and enzymes (see, for instance, refs (29) and (31)). In iGluRs, the large majority of previously developed photoswitchable compounds are competitive agonists and antagonists,^61,65,66,74,75^ with a few compounds targeting the ion channel pore.^74,76,77^ However, none of these compounds display subtype selectivity. In this work, we have developed the first photoswitchable allosteric modulator targeting a specific subtype of NMDARs. In addition to being more specific than competitive ligands and pore blockers, allosteric modulators preserve the endogenous patterns of receptor activity, since they do not prevent binding of the endogenous agonists.^78,79^ This limits the toxicity and desensitization linked to untimed receptor activation/inhibition.

In conclusion, OptoNAM-3 combines the high potency and selectivity of classical GluN2B-selective NMDAR antagonists but with faster kinetics of action, improved reversibility, and red-shifted properties that make it highly biocompatible. This compound should therefore allow selective, local, and temporally controlled inhibition of GluN2B-NMDARs in the mammalian brain. It should thus contribute to a better understanding of the physio-pathological roles of this class of receptors in the brain, as well as to better tolerated drugs to counteract neurological diseases linked to NMDAR overactivation.

## Methods

### Chemicals

Salts, d-serine, DTPA (diethylenetriamine-pentaacetic acid), glucose, l-glutamate, glycine, MTT (3-(4,5-dimethylthiazol-2-yl)-2,5-diphenyl tetrazolium bromide), uridine, and 5-fluoro-2′-deoxyuridine were purchased from Sigma-Aldrich (St. Louis, MO, USA); d-APV (d-(−)-2-amino-5-phosphonopentanoic acid) and *N*-methyl-d-aspartate from HelloBio (County Meath, ROI); and gentamycin from GIBCO (Invitrogen, Rockville, MD, USA). Ifenprodil is a gift from Sanofi-Synthelabo (Bagneux, France). Stock solutions of l-glutamate and glycine (100 mM or 1 M each), DTPA (10 mM to 1 M at pH 7.3), and d-APV (100 mM) were prepared in bidistilled water. OptoNAM (see below) and ifenprodil stock solutions were prepared by diluting the powder in DMSO to concentrations of 50 mM and 20 mM, respectively. They were divided in 0.5 mL aliquots and stored at −20 °C until the day of the experiment.

OptoNAM-1 ((*E*)-2-(phenyldiazenyl)pyridin-4-amine, Mw = 198.23 g mol^–1^), OptoNAM-2 ((*E*)-4-methyl-6-(phenyldiazenyl)pyridin-2-amine, Mw = 212.26 g mol^–1^), OptoNAM-3 ((*E*)-*N*-(4-(phenyldiazenyl)phenethyl), Mw = 302.38 g mol^–1^), and OptoNAM-4 ((*E*)-*N*-(2-(4-(phenyldiazenyl)benzyl)-1*H*-benzo[*d*]imidazol-6-yl)methanesulfonamide, Mw = 405.48 g mol^–1^) were obtained from custom synthesis by Enamine Ltd. (Kiev, Ukraine). Compound characterizations (^1^H and ^13^C NMR) are described Pages S3–S18 and Spectra S1–S4. The purity of the tested compounds, OptoNAM1–4, was established by analytical HPLC-MS (Spectra S1K, S2B, S3O,P, and S4B) and was greater at 95%.

^1^H NMR spectra from Enamine were recorded on a Varian Unity Inova 400 MHz (for OptoNAM-1 to OptoNAM-3) and a Varian VNMRS 500 MHz (for OptoNAM-4). Additional NMR spectra ^1^H (500.16 MHz) and ^13^C (125.78 MHz) for OptoNAM-1 and OptoNAM-3 were recorded on an Avance II 500 Bruker spectrometer. Chemical shifts (δ, ppm) are given with reference to deuterated solvents for ^1^H and ^13^C NMR, respectively: DMSO-*d*_6_: 2.50, 39.51. Signal multiplicity is described as follows: s (singlet), d (doublet), t (triplet), and m (multiplet). Coupling constants (*J*) are given in hertz. Molecule numbering is only related to atom assignment (see Supporting Information), which was established on the basis of ^13^C using ^1^H decoupled spectra as well as correlation spectroscopy, heteronuclear single quantum coherence, and heteronuclear multiple bond coherence.

Mass spectra were recorded on an Orbitrap Exploris 120 (Thermoscientifc) mass spectrometer with positive (ESI+) electrospray ionization (ionization tension, 3.5 kV; ion transfer tube temperature, 325 °C). HPLC-MS analyses were performed on an Orbitrap Exploris 120 Instrument as described above, equipped for HPLC with a Phenomenex Kinetex C18 column (50 mm × 2.1 mm, 2.6 μm). Products were eluted with the following gradient using solvent A (H_2_O/HCO_2_H: 100/0.1) and solvent B (MeCN/HCO_2_H: 100/0.1): 10% B linear increase to 100% B for 15 min, 100% B from 15 to 20 min, and linear decrease to 10% B and 10% B from 20 to 30 min.

#### General Procedure A: Mills Reaction in Basic Medium

To a solution of aminopyridine derivative (1 equiv) and nitrosobenzene (1.2 equiv) in toluene (0.25 M), 5 M aqueous sodium hydroxide (1 M) was added and the resulting mixture was stirred at 50 °C for 3 h and then at 80 °C for 12 h. After cooling to the room temperature, water was added and then the aqueous layer was separated, washed with ethyl acetate, and acidified with NaHSO_4_ to pH 2–3. The resulting precipitate was filtered, washed with water, and dried to give the phenyldiazenyl derivative.

#### General Procedure B: Curtius Rearrangement

To a solution of the phenyldiazenyl derivative (1 equiv), triethylamine (1.1 equiv), and *t*BuOH (10 equiv) in toluene (0.2 M), DPPA (1.1 equiv) was added and the resulting mixture was stirred at 75 °C for 3 h and at 100 °C for 12 h. The solvent was evaporated and the residue was diluted with ethyl acetate. The mixture was washed with aqueous K_2_CO_3_ to pH > 7 (3×) and brine, dried over Na_2_SO_4_, filtered, and concentrated. The crude product was purified by column chromatography to give the *tert*-butyl carbamate derivative.

#### General Procedure C: Boc Deprotection

To a solution of a *tert*-butyl carbamate derivative in methanol, an aqueous solution of 6 M hydrochloric acid was added. The mixture was stirred overnight at room temperature and evaporated under reduced pressure. The residue was dissolved in water and basified with aqueous K_2_CO_3_. The product was extracted with ethyl acetate (2×). The combined organic phases were washed with water, brine, dried over Na_2_SO_4_, filtered, and concentrated *in vacuo*. The crude product was purified by column chromatography to give the OptoNAM-1 to OptoNAM-3.

#### General Procedure D: Hydrogenolysis of the Nitro Group

A mixture of nitrobenzene derivative in methanol was hydrogenated in the presence of Pd/C 10% under balloon pressure of dihydrogen overnight. After filtering off the catalyst, the filtrate was evaporated to dryness to give the aniline derivative in quantitative yield.

#### (*E*)-2-(Phenyldiazenyl)isonicotinic Acid (**5**)

The General Procedure A was followed using methyl 2-aminoisonicotinate (1.52 g, 9.99 mmol) and nitrosobenzene (1.28 g, 12.0 mmol) to get compound **5** (1.82 g, 8.01 mmol) in 80% yield.

#### *tert*-Butyl (*E*)-(2-(Phenyldiazenyl)pyridin-4-yl)carbamate (**6**)

The General Procedure B was followed using compound **5** (1.82 g, 8.01 mmol), triethylamine (0.890 g, 8.80 mmol), *t*BuOH (7.60 mL, 80 mmol), and DPPA (2.42 g, 8.79 mmol) to give compound **6** (1.80 g, 6.00 mmol) in 75% yield.

#### (*E*)-2-(Phenyldiazenyl)pyridin-4-amine (OptoNAM-1)

The General Procedure C was followed using compound **6** (0.900 g, 3.02 mmol), methanol (10 mL), and 6 M HCl (2 mL) to provide the **OptoNAM-1** (0.300 g, 1.52 mmol) in 51% yield. ^1^H NMR (500 MHz, DMSO-*d*_*6*_) δ: 8.12 (d, *J*_H-6,H-5_ = 5.5 Hz, 1H, H-6), 7.88 (m, 2H, H-8, H-12), 7.64–7.58 (m, 3H, H-9, H-10, H-11), 6.83 (d, *J*_H-3,H-5_ = 2.0 Hz, 1H, H-3), 6.62 (dd, *J*_H-5,H-6_ = 5.5 Hz, *J*_H-5,H-3_ = 2.5 Hz, 1H, H-5), 6.36 (s, 2H, NH_2_); ^13^C NMR (126 MHz, DMSO-*d*_6_) δ: 164.1 (C-2), 156.2 (C-4), 151.8 (C-7), 149.0 (C-6), 131.9 (C-10), 129.5 (C-9, C-11), 122.6 (C-8, C-12), 110.4 (C-5), 97.2 (C-3); HRMS (ESI^+^) *m*/*z*: calcd for [C_11_H_10_N_4_ + H]^+^: 199.0984, found 199.0978 (mass error −3.0 ppm); HPLC–MS (ESI) *m*/*z*; (λ = 235 nm): Rt = 3.58 min; 199.0978 [M + H]^+^.

#### Methyl 6-chloro-4-methylpicolinate (**7**)

To a solution of 6-chloro-4-methylpicolinic acid (1.72 g, 10.0 mmol, 1 equiv) in methanol (30 mL), SOCl_2_ (3.60 mL, 49.6 mmol, 4.6 equiv) was added at 0 °C and the resulting mixture was stirred at reflux for 16 h then evaporated under reduced pressure. To the residue, water and aqueous NaHCO_3_ were added until pH > 7, and the mixture was extracted with ethyl acetate (2×). The combined organic phases were washed with water and brine, dried over Na_2_SO_4_ and filtered, and concentrated to give compound **7** (1.70 g, 9.10 mmol) in 91% yield.

#### Methyl 6-bromo-4-methylpicolinate (**8**)

To a solution of compound **7** (1.70 g, 9.16 mmol, 1 equiv) in propionitrile (20 mL), TMSBr (4.20 g, 27.3 mmol, 3 equiv) was added dropwise. The reaction mixture was refluxed for 4 h, cooled to room temperature, and evaporated under reduced pressure. The solid was washed with *n*-hexane to give compound **8** (2.07 g, 9.00 mmol) in 99% yield.

#### Methyl 6-amino-4-methylpicolinate (**9**)

A mixture of compound **8** (2.07 g, 9.0 mmol, 1 equiv), benzophenone imine (1.81 g, 10.0 mmol, 1.1 equiv), and Cs_2_CO_3_ (4.40 g, 13.5 mmol, 1.5 equiv) in toluene (50 mL) was degassed with argon for 10 min. Then, Pd_2_(dba)_3_ (0.410 g, 0.448 mmol, 0.05 equiv) and BINAP (0.560 g, 0.899 mmol, 0.1 equiv) were added and the reaction mixture was stirred under argon at 90 °C for 12 h. After cooling to room temperature, the insoluble material was filtered off, and to the filtrate was added an aqueous solution of 2 M HCl (50 mL, 100 mmol). The resulting mixture was stirred at room temperature for 30 min, and the aqueous layer was separated and washed with ethyl acetate (50 mL), quenched with saturated NaHCO_3_ solution until pH > 7, extracted with ethyl acetate, washed with water and brine, dried over Na_2_SO_4_, and filtered and concentrated. The residue was purified by silica gel column chromatography to give compound **9** (0.700 g, 4.20 mmol) in 47% yield.

#### (*E*)-4-Methyl-6-(phenyldiazenyl)picolinic Acid (**10**)

The General Procedure A was followed using compound **9** (0.700 g, 4.20 mmol) and nitrosobenzene (0.540 g, 5.04 mmol) to give compound **10** (0.720 g, 2.98 mmol) in 72% yield.

#### *tert*-Butyl (*E*)-(4-Methyl-6-(phenyldiazenyl)pyridin-2-yl)carbamate (**11**)

The General Procedure B was followed using compound **10** (0.720 g, 2.98 mmol), triethylamine (0.330 g, 3.26 mmol), *t*BuOH (2.9 mL, 30 mmol), and DPPA (0.910 g, 3.30 mmol) to get compound **11** (0.650 g, 2.08 mmol) in 70% yield.

#### (*E*)-4-Methyl-6-(phenyldiazenyl)pyridin-2-amine (OptoNAM-2)

The General Procedure C was followed using compound **11** (0.312 g, 0.999 mmol), methanol (5 mL), and 6 M HCl (1 mL) to give the **OptoNAM-2** (0.100 g, 0.470 mmol) in 47% yield. ^1^H NMR (400 MHz, DMSO-*d*_*6*_) δ: 7.85 (m, 2H, H_Ar_), 7.57 (m, 3H, H_Ar_), 6.79 (s, 1H, H_Ar_), 6.42 (s, 1H, H_Ar_), 6.22 (s, 2H, NH_2_), 2.25 (s, 3H, CH_3_); HRMS (ESI^+^) *m*/*z*: calcd for [C_12_H_12_N_4_ + H]^+^: 213.1140, found 213.1136 (mass error −1.9 ppm); HPLC–MS (ESI) *m*/*z*; (λ = 235 nm): Rt = 5.30 min; 213.1136 [M + H]^+^.

#### 2-(4-Nitrophenyl)-*N*-(pyridine-4-yl)acetamide (**12**)

To a mixture of 4-aminopyridine (0.940 g, 10.0 mmol, 1 equiv) and 4-nitrophenylacetic acid (1.81 g, 10.0 mmol, 1 equiv) in THF (30 mL), DCC (2.26 g, 11.0 mmol, 1.1 equiv) was added at 0 °C under argon, and the mixture was stirred overnight at room temperature. The insoluble solid was filtered off and washed with THF (10 mL), and the filtrate was evaporated. To the residue was added CHCl_3_ (50 mL), after which the obtained solid was filtered off, washed with CHCl_3_ (20 mL), and dried to give compound **12** (1.75 g, 6.80 mmol) in 68% yield.

#### *N*-(4-Nitrophenylethyl)pyridin-4-amine (**13**)

To a solution of compound **12** (1.75 g, 6.80 mmol, 1 equiv) in THF (50 mL), borane dimethyl sulfide (2.60 g, 34.0 mmol, 5 equiv) was added dropwise at 0 °C and the mixture was stirred for 2 h at room temperature and for 3 h at 65 °C. After cooling to 0 °C, an aqueous solution of 2 M HCl (50 mL) was added dropwise and the mixture was stirred at room temperature for 2 h. The reaction mixture was then quenched with an aqueous solution of 2 M NaOH (100 mL) and extracted with ethyl acetate. The organic layers were then combined, washed with water and brine, dried over anhydrous Na_2_SO_4_, and concentrated. The crude material was purified by silica gel column chromatography to give compound **13** (0.970 g, 3.99 mmol) in 59% yield.

#### *tert*-butyl (4-Nitrophenylethyl)pyridin-4-yl)carbamate (**14**)

To a solution of compound **13** (0.970 g, 3.99 mmol, 1eq) and Boc_2_O (0.872 g, 4.00 mmol, 1 equiv) in THF (20 mL), DMAP (3–5 mg) was added and the resulting mixture was stirred at 65 °C for 3 h. The solvent was evaporated under reduced pressure to give compound **14** (1.37 g, 3.99 mmol) in quantitative yield.

#### *tert*-butyl (4-Aminophenylethyl)(pyridin-4-yl)carbamate (**15**)

The General Procedure D was followed using compound **14** (1.37 g, 3.99 mmol), methanol (30 mL), and Pd/C 10% (0.150 g) to give compound **15** (1.25 g, 3.99 mmol).

#### *tert*-butyl (*E*) (4-(Phenyldiazenyl)phenylethyl)(pyridin-4-yl)carbamate (**16**)

To a solution of compound **15** (1.25 g, 3.99 mmol) in glacial acetic acid (10 mL), nitrobenzene (0.640 g, 5.98 mmol, 1.5 equiv) was added and the mixture was stirred for 8 h at room temperature. The reaction mixture was then quenched with satd. NaHCO_3_ solution (100 mL) and extracted with ethyl acetate. The combined organic layers were dried over anhydrous Na_2_SO_4_ and concentrated. The crude material was purified by silica gel column chromatography to give compound **16** (1.13 g, 2.81 mmol) in 70% yield.

#### (*E*)-*N*-(4-(Phenyldiazenyl)phenethyl)pyridin-4-amine (OptoNAM-3)

The General Procedure C was followed using compound **16** (0.200 g, 0.497 mmol), methanol (5 mL), and 6 M HCl (1 mL). The crude product was purified by silica gel column chromatography followed by reverse-phase HPLC to give the **OptoNAM-3** (0.050 g, 0.165 mmol) in 33% yield. ^1^H NMR (500 MHz, DMSO-*d*_*6*_) δ: 8.02 (d, *J*_H-2,H-3_ = *J*_H-6,H-5_ = 6.5 Hz, 2H, H-2, H-6), 7.88 (m, 2 H, H-16, H-20), 7.84 (d, *J*_H-11,H-10_ = *J*_H-13,H-14_ = 8.5 Hz, 2H, H-11, H-13), 7.62–7.54 (m, 3H, H-17, H-18, H-19), 7.50 (d, *J*_H-10,H-11_ = *J*_H-14,H-13_ = 8.5 Hz, 2H, H-10, H-14), 6.60 (t, *J*_NH,H-7_ = 5.5 Hz, 1H, NH), 3.37 (m, 2H, H-7), 2.94 (t, *J*_H-8,H-7_ = 7.0 Hz, 2H, H-8); ^13^C NMR (126 MHz, DMSO-*d*_*6*_) δ: 153.3 (C-4), 151.9 (C-15), 150.5 (C-12), 149.4 (C-2, C-6), 143.5 (C-9), 131.3 (C-18), 129.8 (C-10, C-14), 129.4 (C-17, C-19), 122.6 (C-11, C-13), 122.4 (C-16, C-20), 107.1 (C-3, C-5), 42.9 (C-7), 34.3 (C-8); HRMS (ESI^+^) *m*/*z*: calcd for [C_19_H_18_N_4_ + H]^+^: 303.1610, found 303.1603 (mass error −2.3 ppm); HPLC–MS (ESI) *m*/*z*; (λ = 235 nm): Rt = 7.61 min; 303.1603 [M + H]^+^.

#### *tert*-Butyl (4-((6-Nitro-1*H*-benzo[*d*]imidazol-2yl)methyl)phenyl)carbamate (**17**)

Methyl chloroformate (0.284 g, 3.00 mmol, 1 equiv) was added to a mixture of 2-(4-((*tert*-butoxycarbonyl)amino)phenyl)acetic acid (0.753 g, 3.00 mmol, 1eq), triethylamine (0.303 g, 2.99 mmol, 1 equiv), and DMF (10 mL) at −20 °C. After 15 min of stirring, 4-nitrobenzene-1,2-diamine (0.46 g, 3.00 mmol, 1 equiv) was added and the reaction mixture was stirred at 20 °C for 4 h. The volatiles were evaporated and the residue was partitioned between water and EtOAc. The organic layer was washed with 5% aqueous NaHCO_3_, brine, and water; then dried over Na_2_SO_4_; and concentrated in vacuo to afford the amino amide. A solution of crude amino amide in glacial acetic acid (10 mL) was heated at 65 °C for 12 h, and then, the volatiles were evaporated and the residue was partitioned between water and EtOAc. The organic layer was washed with water, dried over Na_2_SO_4_, and then concentrated under reduced pressure. The crude material was then purified by silica gel column chromatography to give compound **17** (0.446 g, 1.32 mmol) in 44% yield.

#### *tert*-Butyl (4-((6-Amino-1*H*-benzo[*d*]imidazol-2yl)methyl)phenyl)carbamate (**18**)

The General Procedure D was followed using compound **17** (0.368 g, 1.0 mmol), MeOH (10 mL), and Pd/C 10% (0.05 g) to give compound **18** (0.338 g, 1.0 mmol) in quantitative yield.

#### *tert*-Butyl (4-((6-Methylsulfonamido)-1*H*-benzo[*d*]imidazol-2yl)methyl)phenyl)carbamate (**19**)

To a cooled and magnetically stirred solution of compound **18** (0.338 g, 1 mmol, 1 equiv) in CH_2_Cl_2_ (10 mL) were successively added pyridine (0.12 g, 1.5 mmol, 1.5 equiv) and methanesulfonyl chloride (0.137 g, 1.2 mmol, 1.2 equiv). The resulting mixture was allowed to stand at room temperature for 16 h. Water was added and the aqueous layer was separated and washed with CH_2_Cl_2_. The combined CH_2_Cl_2_ layers were successively washed with water, aqueous NaHSO_4_, and brine; dried over Na_2_SO_4_; and filtered and concentrated to give compound **19** (0.395 g, 0.95 mmol) in 95% yield.

#### HCl·*N*-(2-(4-Aminobenzyl)-1*H*-benzo[*d*]imidazol-6-yl)methanesulfonamide (**20**)

To a solution of compound **19** (0.208 g, 0.5 mmol) in MeOH (5 mL) was added 4 M HCl in dioxane (2 mL) and stirred at room temperature for 12 h. The solvent was evaporated under reduced pressure and the residue was dried to give compound **20** (0.176 g, 0.5 mmol) in quantitative yield, as hydrochloride form.

#### (*E*)*-N*-(2-(4-(Phenyldiazenyl)benzyl)-1*H*-benzo[*d*]imidazol-6-yl)methanesulfonamide (OptoNAM-4)

Compound **20** (0.074 g, 0.2 mmol, 1 equiv) in hydrochloride form and KOAc (0.06 g, 0.6 mmol, 3 equiv) were dissolved in glacial acetic acid (3 mL) at room temperature. Nitrosobenzene (0.064 g, 0.6 mmol, 3eq) was added to this solution in one portion, and the reaction mixture was stirred for 8 h, then quenched with saturated aqueous NaHCO_3_ solution, and extracted with ethyl acetate. The combined organic layers were dried over anhydrous Na_2_SO_4_ and concentrated under reduced pressure. The crude material was then purified by silica gel column chromatography to give the target **OptoNAM-4** (0.015 g, 0.037 mmol) in 19% yield. ^1^H NMR shows the presence of the two tautomers of benzimidazole (see Spectrum S4A). ^1^H NMR (400 MHz, DMSO-*d*_*6*_) δ 12.50, 12.40 (2s, 1H, NH_Im_), 9.50, 9.40 (2s, 1H, NHSO_2_), 8.00–6.70 (m, 12H, H_Ar_), 4.30 (s, 2H, CH_2_), 2.88, 2.86 (2s, 3H, CH_3_); HRMS (ESI^+^) *m*/*z*: calcd for [C_21_H_19_N_5_O_2_S+ H]^+^: 406.1358, found 406.1332 (mass error −1.5 ppm); HPLC–MS (ESI) *m*/*z*; (λ = 235 nm): Rt = 7.30 min; 406.1332 [M + H]^+^.

### Spectroscopic Analyses of OptoNAMs

UV–vis absorbance spectra were measured from in 1 cm long quartz cuvettes on a NanoPhotometer NP80 spectrometer (Implen, Germany). We used solutions of 10, 50, or 100 μM OptoNAMs diluted in Ringer at pH 7.3 (for TEVC extracellular recording solution, see below) from the 50 mM stock solution in DMSO. Photostationary states of OptoNAMs in Ringer for different illumination wavelengths were obtained by continuous illumination with multiwavelength LEDs (pE-2 and pE-4000, CoolLED, UK [power ∼75 mW]) or photochemical reactor RPR-100 Rayonet US (power ∼2 mW) for 350 nm, until no further change in the absorption spectra could be observed. For all irradiation wavelengths tested, 10 min illumination was sufficient to reach the steady state.

The *cis*–*trans* compositions of OptoNAM-3 in the dark and after illumination by 365, 350, and 550 nm were determined by HPLC using the relative integrated areas of the *cis* and *trans* peaks at the isosbestic point at 280 nm, where the isomers have the strongest absorbance, considering that the sum of integrated area for two peaks is 100%. We checked that ratios measured at the isosbestic point at 390 nm were similar. Analytical HPLC was performed on an Agilent 1200 series equipped with a quaternary pump using a Nucleodur C18 HTech column from Macherey-Nagel Inc. (particle size 3 μm, 150 × 4.6 mm column). The compounds were eluted with a flow of 1 mL/min using a gradient of acetonitrile (0–100% over 15 min) in water, with both solvents containing 0.1% TFA. Detection was performed at 220, 280, and 390 nm.

The dark and 365 nm PSS were furthermore characterized by ^1^H NMR spectroscopy with the OptoNAMs diluted in DMSO-*d*6 and the relative abundance of *trans* and *cis* isomers quantified using ^1^H NMR spectra. Indeed, because the aromatic protons of *trans* and *cis* exhibit sufficiently different chemical shifts, their relative abundances at each PSS can be determined by peak integration (see Spectra S1G and S3K).

The *cis*–*trans* composition of OptoNAM-3 after illumination at other wavelengths was calculated from their UV–vis spectra using the following formula, considering from the HPLC analysis that the dark PSS is composed of 100% *trans* and the 365 nm state is composed of 18% of *trans*: %*trans* = (*A*_λ_ – *p*)/(*A*_dark_ – *p*), where *p* represents the theoretical absorbance of a pure *cis* population, *p* = (*A*_UV_ – 0.18 *A*_dark_)/0.82, and *A*_λ_, *A*_UV_, and *A*_dark_ represent the absorbances of the compounds after illumination at a given wavelength after illumination at 365 nm and in the dark, respectively, measured at the absorption peak of the *trans* state (329 nm).

Thermal stability of the *cis* state (365 nm PSS) was measured by irradiating the solution with 365 nm light during 10 min, then letting it relax in the dark, at room temperature (20–22 °C), inside the spectrophotometer. Spectra were acquired at regular intervals, up to 1 day, after irradiation.

### Molecular Biology

We used cDNA coding for rat GluN1–1a (named GluN1 herein) (GenBank: U08261.1), rat GluN2A (GenBank: D13211.1), mouse GluN2B (GenBank: D10651.2), rat GluA1 flop (GenBank: M36418.2), and rat Myc-GluA2(Q)-flip (GenBank: AF164344.1) cloned into pcDNA3-based expression plasmids. GluK2 (GenBank: Z11548.1) corresponds to the fully RNA-edited version.^80^ The GluN2B-ΔNTD construct is from ref^45^ and GluN2B–N615K is from ref^81^. Single mutants GluN2B-Q110G and GluN1-L135H were from ref^19^. The GluA2 cDNA contains a Myc tag (EQKLISEEDL) fused between the signal peptide and the N-terminus sequences, and the GluA2 subunit is edited for its Q/R/N site (GluA2(Q)) to allow permeation of homomeric GluA2 receptors.^2^ cDNAs coding for rat GluN2C (GenBank: M91563.1) and GluN2D subunits (GenBank: L31611.1) were in prK5 plasmids.

### Oocyte Treatment and Microinjection

Oocytes from *Xenopus laevis* were used for heterologous expression of recombinant NMDA receptors studied using a two-electrode voltage clamp (TEVC). Female *Xenopus laevis* were housed and ovary bags harvested according to the European Union guidelines (husbandry authorizations #C75–05–31 and #D75–05–31; project authorizations #05137.02; and Apafis #28867–2020121814485893). Fragments of ovary bags were also purchased from the “Centre de Ressources Biologiques Xenopes” (now TEFOR, Paris Saclay, France) and from the European Xenopus Resource Center (EXRC, Portsmouth, UK). *Xenopus laevis* oocytes were harvested and prepared as previously described in ref^82^.

Expression of recombinant NMDA receptors was obtained by oocyte nuclear coinjection of 37 nL of a mixture of cDNAs (at 30 ng/μL) coding for GluN1–1a and various GluN2 subunits (ratio 1:1). The protocol in ref^43^ was followed for the expression of GluN1/GluN2A/GluN2B triheteromers. The oocytes were transferred in 96-well plates filled with Barth solution (in mM: 88 NaCl, 1 KCl, 0.33 Ca(NO_3_)_2_, 0.41 CaCl_2_, 0.82 MgSO_4_, 2.4 NaHCO_3_, and 7.5 HEPES, pH adjusted to 7.3 with NaOH), supplemented with gentamicin (50 μg/μL) and 50 μM APV, a selective NMDA receptor antagonist. Plates were then stored at 18 °C for 24 h for GluN1/GluN2A expression and for 48––72 h for the expression of GluN1/Glu2B, GluN1/GluN2A/GluN2B, and mutants.

The expression of recombinant GluA1, GluA2(Q), and GluK2 receptors was obtained by oocyte injection of 50 nL of mRNAs (at 1 μg/μL). mRNAs were generated using the T7 (GluA1 and GluA2) and T3 (GluK2) mMessage mMachine transcription kits (Invitrogen) after cDNA linearization with the SacII restriction enzyme. The injected oocytes were stored in the same conditions as for NMDARs and used 72–96 h postinjection.

### Two-Electrode Voltage Clamp and Recording Solutions

TEVC recordings were performed 1–3 days following injection. TEVC recordings were performed using an Oocyte Clamp amplifier OC-725 (Warner Instruments) and computer-controlled via a 1440A Digidata (Molecular Devices). Currents were sampled at 100 Hz and low-pass-filtered at 20 Hz using an 8-pole Bessel filter (900 Series, Frequency Devices Inc.). Data were collected with Clampfit 10.3. During the recording, the cells were continuously perfused with external recording Ringer solution at pH 7.3 (in mM: 100 NaCl, 0.3 BaCl_2_, 5 HEPES, and 2.5 KCl, pH adjustment to 7.3 by the addition of NaOH). The NMDA currents were induced by simultaneous application of l-glutamate and glycine (agonist solution) at saturating concentration (100 μM each), and DTPA (10 μM) to prevent receptor inhibition by ambient zinc (∼20 nM).^83^ A control solution (100 μM glycine and 10 μM DTPA in Ringer pH 7.3) was used for washout of drug and test solutions. AMPA and kainate receptor currents were induced in the same conditions as NMDA currents (100 μM glutamate and glycine + 10 μM DTPA). Oocytes expressing GluK2 kainate receptors were treated with concanavalin A (2 mM, equivalent to 1.1 mg/mL) during 10 min before recording to decrease receptor desensitization. Unless notified, recordings were performed at a holding potential of −60 mV. All experiments were performed at room temperature.

OptoNAMs were diluted as stock solutions of 50 to 0.5 mM in DMSO. On the day of the experiment, they were diluted to the appropriate concentration in the recording solution and kept in the dark during the whole duration of the experiment to avoid photoconversion. Since DMSO itself induces a small inhibition of NMDAR currents, control and agonist solutions were also supplemented with DMSO up to 0.1%. 365 nm PSS solution was obtained by irradiating from the top 25 mL of *trans*-OptoNAM solutions with a 365 nm light LED (pE-2, CoolLED, UK, power ∼75 mW) for 10 min in a graduated cylinder covered with aluminum foil. To obtain 350 nm PSS solutions, solutions containing OptoNAM *trans* were put in a quartz cuvette in a photochemical reactor (RPR-100, Rayonet, US, irradiance power ∼2 mW) at 5 cm from a 350 nm neon for 15 min. Recordings with 350 nm PSS were performed within 20 min after 350 nm irradiation to avoid photoconversion.

### Whole-Cell Patch-Clamp Recordings and Photomodulation in HEK Cells

HEK293 cells were used for heterologous expression of recombinant NMDA receptors studies using whole-cell patch-clamp. Wild-type NMDARs were expressed in HEK-293 cells (obtained from ECACC, Cat #96121229). HEK cells were cultured in DMEM + glutamax medium supplemented with 10% fetal bovine calf serum and 1% penicillin/streptomycin (5000 U/mL) and cultured under standard cell culture conditions (5% CO_2_, 37 °C). Transfections were performed using polyethylenimine (PEI) in a cDNA/PEI ratio of 1:3 (v/v). Cells were cotransfected with a DNA mixture containing plasmids encoding wild-type GluN1, wild-type GluN2A or GluN2B, and eGFP. The total amount of DNA was 1.0 μg per 500 μL of transfected medium containing 12 mm^2^ diameter coverslip, and the mass ratio of GluN1:GluN2B:eGFP was 1:2:1 (1:1:1 for GluN2A). 150 μM of d-APV was added to the culture medium after transfection.

Receptor functionality, OptoNAM-3 photodependence (dark then UV light) of activity, and association/dissociation kinetics (with and without UV light) were assessed in patch-clamp recordings of lifted whole cells 24–72 h post transfection (cells were not lifted for the 2 light cycles protocol, i.e., when “perfusion turned off” was written below current traces). Positively transfected cells were visualized by GFP fluorescence. The extracellular solution contained (in mM) 140 NaCl, 2.8 KCl, 1 CaCl_2_, 10 HEPES, 20 sucrose, and 0.01 DTPA (290–300 mOsm), pH adjusted to 7.3 using NaOH. Patch pipettes had a resistance of 3–6 MΩ (whole-cell) and were filled with a solution containing (in mM) 115 CsF, 10 CsCl, 10 HEPES, and 10 BAPTA (280–290 mOsm), pH adjusted to 7.2 using CsOH. Currents were sampled at 10 kHz and low-pass filtered at 2 kHz using an Axopatch 200B amplifier, a 1550B Digidata, and Clampex 10.6 (Molecular Devices). Agonists (100 μM glutamate and 100 μM glycine) were applied using a multibarrel solution exchanger (RSC 200; BioLogic). Recordings were performed at a holding potential of −60 mV and at room temperature.

Computer-controlled light pulses during electrophysiological recordings were provided from high power sensitive LEDs (CoolLED pE-4000: 4 channels each controlling 4 wavelengths from 365 to 770 nm). The LED port was directly coupled via a microscope adaptor to the fluorescence port of an inverted IX73 Olympus microscope. The output beam of the LED entry was directed toward the sample thanks to a mirror (Chroma) and applied to the center of the recording dish through a 10× objective (irradiance ∼4 mW/mm^2^ for 365, 385, and 435 nm; ∼6.5 mW/mm^2^ for 460 nm; ∼2 mW/mm^2^ for 490 and 525 nm; ∼1.5 mW/mm^2^ for 500 nm, and ∼4.5 mW/mm^2^ for 550 and 580 nm) (Olympus, 0.30 N.A.). Light power was measured in the center of the recording dish plane with an optical power meter (1916-C, Newport) equipped with a calibrated UV/D detector, and irradiance was obtained upon dividing light power by the illuminated field of the microscope 10× objective (19 mm^2^).

### Primary Cortical Neuron Cultures

Mice were housed in the IBENS rodent central facility duly accredited by the French Ministry of Agriculture. All experiments were performed in compliance with French and European regulations on care and protection of laboratory animals (EU Directive 2010/63, French Law 2013–118, February 6, 2013), and were approved by local ethics committees and by the French Ministry of Research and Innovation (authorization numbers #05137.02, APAFIS #28867-2020121814485893). Dissociated cultures of cortical neurons were prepared from mouse embryos at E18 as described previously.^84^ After removing meninges, cortices were placed in ice-cold HBSS solution. Cell dissociation was performed individually for cortices of each embryo. Cortices were incubated in 2.5% trypsin at 37 °C for 7 min, rinsed three times with 37 °C phosphate buffer saline, and finally suspended in plating medium (Neurobasal Medium, supplemented with 0.5 mM l-glutamine, 1% B27 supplement, and penicillin/streptomycin). The neurons were further dissociated by trituration, and cells were plated on poly-d-lysine coated coverslips in 24-well culture dishes at a density of 3 × 10^5^ cells per well for the excitotoxicity test and that of 1 × 10^5^ cells per well for patch clamp experiments. Cultures were incubated at 37 °C in a humidified atmosphere of 5% CO_2_. Cells were fed by changing 1/2 medium to fresh Neurobasal medium every 4 days. For the excitotoxicity experiments, after 5 days in vitro, growth of non-neuronal cells was halted by a 24 h exposure to FDX (5 mM uridine and 5 mM 5-fluoro-2′-deoxyuridine). The cultures were used for experiments after 6 to 14 days in vitro (DIV6–8 for patch clamp experiments and DIV14 for excitotoxicity tests).

### Whole-Cell Patch-Clamp Recordings in Wild Type Cultured Cortical Neurons

Whole-cell patch-clamp recordings on neurons were performed 6 to 8 days after the culturing step (DIV 6–8) using the same recording conditions as for HEK cells. Recordings were performed at a holding potential of −60 mV and at room temperature. Currents were elicited by NMDA (300 μM) and d-serine (50 μM) to specifically activate NMDARs.

#### *Trans* to *Cis* Isomerization Study

OptoNAM-3 was perfused in the dark onto the patched neuron (Figure 5A–C). Once the inhibition reached steady-state, indicating that the compound was in its binding site, perfusion was stopped and wavelengths from 435 to 580 nm followed by 365 nm (the wavelength allowing optimal *trans*–*cis* isomerization) were applied. Stopping perfusion ensures that trans-OptoNAM coming from the perfusion tube can compete with the wavelength PSS during the illumination procedure.

#### *Cis* to *Trans* Isomerization Study

OptoNAM-3 was perfused in the dark onto the patched neuron (Figures 4G,H and 5F,H). Once the inhibition reached steady-state, indicating that the compound was in its binding site, perfusion was stopped and 365 nm was applied to the cell. Then, wavelengths from 435 to 580 nm were applied and the inhibition recovery was monitored.

### Neuronal Toxicity Experiments and Assessment of Neuronal Death

Excitotoxicity tests were performed 14 days after the culturing step as described in ref^15^. Just before the experiments, the neuronal medium was changed and the neurons incubated in the external patch recording solution (see above). Two replicates of 24 well plates containing cultured cortical neurons were exposed to the same 4 conditions, in which the following compounds were added to the medium: (1) glycine (10 μM) alone (control); (2) glycine (10 μM) + NMDA (100 μM); (3) glycine (10 μM) + NMDA (100 μM) + 5 μM ifenprodil; and (4) glycine (10 μM) + NMDA (100 μM) + 5 μM *trans*-OptoNAM-3 (dark). One of the two plates was exposed to UV light (Jena analytic US, UVP Hand-held UV lamp UVGL-58, power ∼1 mW) for 2 min. The two plates were then put back in the 37 °C incubator for 10 min. Agonist exposure was terminated by washing out the exposure solution with conditioned Neurobasal medium, prior to returning the dishes to the incubator for 24 h before assessment of neuronal death.

Overall neuronal cell death was determined 24 h after NMDA exposure by the MTT test. Mitochondrial and cytosolic dehydrogenases of living cells reduce the yellow tetrazolium salt (MTT) to produce a purple formazan dye that can be detected spectrophotometrically.^85^ MTT was dissolved in phosphate-buffered saline (PBS) buffer at 5 mg/mL and filtered through a 0.2 μm membrane to sterilize it and remove a small amount of insoluble residues present in some batches of MTT. A day after the cell exposure to the different conditions, 50 μL of stock MTT was added to all 500 μL medium containing wells, and plates were incubated at 37 °C for 4 h. The medium was removed, and 200 μL of warm DMSO was added per well and mixed thoroughly to dissolve the dark blue crystals. After 10 min at 37 °C to ensure that all crystals were dissolved, the absorbance of the wells was monitored at 540 nm on a multimode plate reader Infinite 200 PRO R (Tecan, Switzerland).

### Data Analysis

Data were collected and analyzed using pClamp 10.5 (Molecular Devices) and fitted using Sigmaplot 11.0 (SSPS). Unless otherwise mentioned, error bars represent the standard deviation of the mean value. Dose–response curves were fitted with the following Hill equation: , where *I*_rel_ = *I*_antago_/*I*_control_ is the mean relative current, [B] is the drug concentration, (1–*a*) is the maximal inhibition, and *n*_H_ is the Hill coefficient. IC_50_, *a*, and *n*_H_ were fitted as free parameters. IC_50_ errors represent the error of the fit.

Theoretical “pure *cis”* dose–response curves of OptoNAM-3 (Figure 3C) were obtained by calculating the theoretical relative current elicited by a pure OptoNAM *cis* population (*I*_Rel*cis*_) at each concentration (C) data point from the following equation: *I*_Rel*cis*_ = 1 + (*I*_RelUVPSS_ – *I*_Rel*trans*_). I_RelUVPSS_ is the relative current obtained from the 365 nm PSS dose–response curve following application of a concentration *C* of OptoNAM, corresponding to 82% *cis*-OptoNAM and 18% *trans*-OptoNAM (and 91% cis/9% trans if starting from the 350 nm PSS dose–response curve). *I*_Rel*trans*_ is the relative current obtained from the *trans*-OptoNAM dose–response curve (dark) following application of a concentration of 0.18× C of *trans*-OptoNAM-3 (or 0.09× C if deduced from the 350 nm PSS dose–response curve).

The theoretical 18% (365 nm PSS *trans*% obtained by HPLC) and 9% of *trans* (350 nm PSS obtained in HPLC) dose–response curves (Figure S3A,B) were obtained starting from the *trans-*OptoNAM-3 dose–response curve by considering that the dark state of OptoNAM-3 solution is composed of 100% of the *trans* isomer and that each relative current obtained for one concentration *I*_RelC_ is equal to the relative current *I*_Rel%C_ exerted by the application of 18% or 9% of this concentration.

The kinetics of photoinactivation and OptoNAM-3 dissociation (Figure 4E,F) were obtained by fitting currents with a single exponential function as follows: *Y* = *A* × exp(−*t*/τ) + *C*, with *A* as the initial current amplitude, Tau (τ) as the decay time constant, and *C* as the steady-state level.

For the *trans* to *cis* and *cis* to *trans* isomerization study in the binding site (Figure 5C,H respectively), we decided to normalize (scale) the inhibition values of OptoNAM-3 at different wavelengths by the inhibition induced by OptoNAM-3 at 365 nm and in the dark since these two conditions represent respectively the minimum and maximum of the inhibition. We followed this equation:  where *m* ∈ [*r*_365_,*r*_dark_] is the measurement (inhibition) to be scaled, *r*_365_ and *r*_dark_ correspond to the minimum (365 nm state inhibition) and maximum (dark state inhibition) of the measurement, respectively, and *t*_365_ and *t*_dark_ represent the minimum and maximum of the range (OptoNAM-3 365 nm PSS and dark PSS mean inhibition). To reduce the decreasing effect of normalization onto SEM, the SEMs represented in the figures were calculated with the data injected in the first part of the equation ().

To estimate the ratio of *trans*/*cis* isomers obtained upon illumination of bound OptoNAM-3 (Figure 5E,J, respectively), we calculated the residual currents obtained as described in the paragraph above and deduced their corresponding concentrations from the dose–response curve equation of OptoNAM-3 in the dark (assuming the dark state represents 100% *trans*). The obtained concentration was then divided by the initial *trans* concentration (2 μM) to obtain the percentage of *trans* isomer at each wavelength.

For the excitotoxicity test (Figure S4), data were normalized by the mean of control (considered as 100% survival) and NMDA condition (considered as 0% survival) of the well plate. We followed this equation: , where *m* is the measurement (absorbance) to be normalized and moy_NMDA_ and moy_ctrl_ are the mean absorbance of the NMDA and control condition for the well plate, respectively.

### Behavioral Tests in *Xenopus laevis* Tadpoles

*Xenopus laevis* embryos were obtained from the European Xenopus Resource Centre (EXRC, Portsmouth, UK) or TEFOR Paris Sackay (Saclay, France), maintained in Modified Barth’s Saline (MBS) 0.1× (pH adjusted to 7.8 with NaOH), and supplemented with 10 μg/mL of gentamicin, in 10 cm Petri dishes with a maximum of 50 tadpoles per dish. The embryos were stored at 18 °C until they reached stage 49 (12–14 days postfertilization).^86^ Experiments on *Xenopus* tadpoles were performed according to European Union guidelines (project authorization Apafis #44976–2023092616413977)

For the experiments (see Figure S5A), stage 49 *X. laevis* tadpoles were placed in the center of a 12-well plate at a density of three animals per well in 0.1× MBS. The 12-well plate was covered with a 3D-printed dome equipped with a liquid guide (15 cm) connected to a multichannel LED delivery system (CoolLed pE-4000) at the top. The plate was positioned on optical cast plastic infrared filters, which allowed only wavelengths above 650 nm to pass, and red light (770 nm at 15% intensity) was continuously applied during recording to enable video recording in the dark. To habituate the tadpoles, the red light was gradually increased to 15% (0.03 mW) over a 15 min period, and their baseline locomotion was recorded for 3 min. Subsequently, the tadpoles were incubated in the dark for 30 min in a 60 × 15 mm Petri dish containing 5 mL of either the vehicle (0.1% DMSO in MBS 0.1×) or OptoNAM-3 solution (5 μM in MBS 0.1×) to facilitate drug penetration. Afterward, the tadpoles were transferred back to the 12-well plate, which contained either the vehicle solution or OptoNAM-3 (5 μM), and habituation to red light was performed again for 15 min. A 3 min video was then recorded to assess the locomotion in the dark after a 45 min incubation and during cycles of 1 min illumination at 365 nm (or 460 nm) and 550 nm, alternating with 3 min rest periods (365 nm, 460 nm, power ∼ 0.05 mW; 550 nm, power ∼ 0.36 mW). UV–green light cycles were consecutively repeated twice. Following the pharmacological treatments, the tadpoles were returned to water. The next day, the animals were observed for abnormalities.

To extract multianimal tracking and pose estimation from the recorded tadpole videos, we employed the open-source deep learning toolbox DeepLabCut.^57,58^ The toolbox is based on transfer learning and adapted from ImageNet-pretrained ResNets, specifically Resnet50, which is a model pretrained with over a million images. The original videos of the 12-well plates were cropped into individual wells to track the movement of three tadpoles per video. We selected 20 frames per video and labeled them with seven markers representing different body parts (right and left eye, stomach, and 4 points on the tail) to train the model for pose recognition using a multitask convolutional neural network (CNN) that performs pose estimation by localizing key points in images. To enable the connection of key points to a given animal, additional deconvolution layers were added to the program.^57^ Model evaluation was conducted by calculating the pixel error between the predictions and manually labeled frames. Once the model achieved satisfactory training (network evaluation with a 1.3-pixel error for both training and testing), the video was analyzed, and individual tadpole traveled distances were calculated based on the x and y pixel coordinates of the labels provided by the data sheet obtained from video analysis. The stomach traveled distance yielded the best results; therefore, the tadpole traveled distance reported here represents the distance traveled by the “stomach” label. Since the identity of the 3 tadpoles could not be determined across the videos, the mean of the traveled distance per 3 tadpoles in one well was calculated in order to do a paired statistical test between light conditions. In the figures representing the *in vivo* experiment, one point therefore represents the mean locomotion of 3 tadpoles of the well. Tadpole locomotion was quantified by dividing the traveled distance by the recording duration and normalizing it twice: first by their baseline locomotion (Figure S5B,C) and second by the control (Figure 6).

### In Silico Docking and Molecular Dynamics

To model the protein/ligand interactions, we used the crystallographic structure 5EWJ.^19^ Missing residues were modeled by overlapping 5EWJ with the structure predicted by AlphaFold^87^ for the same sequence. Residues 96 to 103 and 184 to 209 from chain A and residues 42 to 65 from chain B were then extracted from the AlphaFold structure and added to the 5EWJ one to obtain a complete protein structure. Three-dimensional structures of the ligands Ifenprodil and OptoNAM-3 were obtained from their SMILES description with Gypsum-DL,^88^ and their protonation states were manually corrected. When four different starting conformations of Ifenprodil were docked in 5EWJ with Vina,^89,90^ only one pose was found similar to the crystallographic conformation of ifenprodil within 5EWJ. However, when these conformations were docked with Gnina^91^ (which is a fork of Vina that uses neural networks for the docking and the scoring), the four poses were found in a similar orientation as the experimental one. Thus, we have used Gnina as a docking engine. We add here that if the starting conformation of ifenprodil is the one extracted from 5EWJ (and not the ones coming from Gypsum-DL), the docked pose perfectly overlaps the crystallographic pose. The Ifenprodil pose with the best score (−11.2) is displayed in Figure S9A in green, together with the crystallographic pose that is displayed in orange. OptoNAM-3 was then docked: 10 different conformations were used, and the most relevant one (with the pyridinium of OptoNAM-3 overlapping with the phenol of ifenprodil, and the two terminal phenyls in the same pocket) is displayed in Figure S9B in purple (score of −9.5).

The geometry of OptoNAM-3 was then optimized at the M06-2X/6-31+G(d,p) level of geometry in a PCM of water with Gaussian09 A.02.^92^ The RESP charges were then obtained at the HF/6-31G(d) level of geometry with Gaussian09 C.01, and acpype^93^ and antechamber^94^ were used to get the GAFF2 force field parameters for OptoNAM-3.^95^ Finally, the force field was modified with an improved description of the azo moiety.^96^

Molecular dynamics (MD) simulations were performed with Gromacs^97−99^ with the Amber14SB force field to describe the protein atoms and ions^100^ and the TIP3P description of water.^101^ After solvation in a rhombic odecahedron box with at least 8 Å between the solute atoms and the edges of the box, the system was neutralized with 18 sodium ions. The energy was then minimized with steepest descent to avoid steric clashes. The system was equilibrated during a 500 ps NPT simulation where we used a simulated annealing procedure to gradually heat the system from 100 to 300 K in 400 ps; the system then evolved in 100 ps at 300 K. During the equilibration, the velocity-rescale thermostat^102^ and the Berendsen barostat^103^ were used, with a time step of 1 fs. Bonds containing an hydrogen were constrained with the LINCS algorithm^104,105^ with default parameters. Nonbonded interactions were described with PME for the electrostatics^106^ with standard values for the force field (i.e., change between direct and reciprocal spaces at 8 Å) and default parameters, and cutoff of van der Waals interactions at 8 Å. We then performed the production simulation during 1 μs, where the parameters were similar to those in the equilibration step except for the barostat, which was changed to Parrinello–Rahman,^107^ and for the time step, which was increased to 2 fs.

### In Silico Quantum Mechanics Simulations

Quantum mechanics (QM) and quantum mechanics/molecular mechanics (QM/MM) calculations were performed using the ORCA 5.0.4 suite of programs.^108^ Geometry optimizations were carried out at the restricted Kohn–Sham density functional theory (DFT) level without the use of symmetry, employing the PBE0 functional,^109^ the def2-TZVP basis set^110^ with matching auxiliary basis sets,^111^ and the D3 correction.^112,113^ The RIJCOSX approach applying the resolution of identity (RI) approximation to the Coulomb part and the chain of spheres (COS) seminumerical integration algorithm to the exchange term was used.^114,115^ The convergence criteria for both the SCF was set to TIGHT and all the other parameters were chosen as default. For time-dependent DFT (TDDFT) calculations, the double-hybrid B2PLYP functional^116,117^ was chosen with at least 5 roots and without the TDA approximation. In more details, for calculations done in water, the linear response conductor-like polarizable continuum model (LR-CPCM) perturbation of the density was included for both DFT and TDDFT calculations.^118,119^ For calculations done in the protein, the ORCA multiscale module was used. QM/MM calculations were performed using the additive scheme together with an electrostatic embedding. OptoNAM-3 consists of the QM level while the rest of the system is treated at the MM level using the AMBER14SB^95,100,120^ force field as required by ORCA software. All the other parameters were chosen as default.

### Statistical Analysis

Data are presented as mean ± standard deviation of the mean (SEM). All sample numbers (*n*) and statistical tests are specified in the figure legends. Statistical significances are indicated with *, **, and *** when *p* values are below 0.05, 0.01, and 0.001, respectively. n.s. indicates nonsignificane. Significance was defined as *p* < 0.05.

## Supporting Information Available

Chemical characterization of the four OptoNAMs (NMR and HPLC-MS spectra); description of the photochemical and biological characterizations of OptoNAMs (Text S1); detailed procedure of docking and molecular simulations of OptoNAM-3 in its binding site (Text S2); photochemical properties of OptoNAM-1 to -4 and their photodependent activity at GluN1/GluN2B receptors (Figure S1); decreased pKa of OptoNAM-1 and -2 compared to their parent compounds (Figure S2); additional data relative to Figure 3 (Figure S3); OptoNAM-3 decreases NMDA-induced neuronal death in a photodependent manner (Figure S4); OptoNAM-3 photomodulates *Xenopus* tadpole locomotion in vivo (Figure S5); OptoNAM-3 photochemical properties in different solvents (Figure S6); evolution of trans-OptoNAM-3 conformation in its binding site and in water (Figure S7); additional data relative to Figure 7 (Figure S8); overlap between crystallographic and docked poses of ifenprodil and overlap between the crystallographic pose of ifenprodil and the docked pose of trans-OptoNAM-3 (Figure S9); summary of the IC50s of OptoNAMs in the dark and UV compared to the activity of their parent compounds (Table S1); computed vertical energies and oscillator strengths of 11 snapshots of free trans-OptoNAM-3 in implicit water (Table S2); computed vertical energies and oscillator strengths of 11 snapshots of bound trans-OptoNAM-3 inside the protein (Table S3); computed vertical energies and oscillator strengths of snapshot 0 of bound trans-OptoNAM-3 (Table S4) (PDF)
Two movies of the effect of OptoNAM-3 on *Xenopus* tadpole locomotion under UV–green and blue–green illumination cycles (ZIP)
Input files for QM/MM simulations (ZIP)
Input files for MD simulations (ZIP)
Source data (XLSX)

## Abbreviations

AcOH: acetic acid
BINAP: ([1,1′-binaphtalene]-2,2′-diyl)bis(diphenylphosphane
Boc_2_O: di-*tert*-butyl dicarbonate
DCC: *N*,*N*′-dicyclohexylcarbodiimide
DFT: density functional theory
DMAP: 4-dimethylaminopyridine
DMF: *N*,*N*′-dimethylformamide
DMSO: dimethyl sulfoxide
DPPA: diphenyl phosphorazidate
eq: equivalent
Et_3_N: triethylamine
HEK cells: human embryonic kidney cells
MeOH: methanol
MD: molecular dynamics
NAM: negative allosteric modulator
NMDA: *N*-methyl-d-aspartate
NTD: N-terminal domain
Pd_2_(dba)_3_: Tris(dibenzylideneacetone)dipalladium
PSS: photostationary state
QM/MM: quantum mechanics/molecular mechanics
RT: room temperature
*t*BuOH: *tert*-butanol
THF: tetrahydrofuran
TMSBr: bromotrimethylsilane
