# Supplementary material for: Reversible Control of Native GluN2B-Containing NMDA Receptors with Visible Light
Source: ACS Chem Neurosci. 2024 Sep 6;15(18):3321–43. doi: 10.1021/acschemneuro.4c00247 (PMC11413854; doi:10.1021/acschemneuro.4c00247)
Supplement: Supplementary file 1 — cn4c00247_si_001.pdf [file cn4c00247_si_001.pdf]

Supporting information

Fast and reversible inhibition of native GluN2B-containing NMDA receptors with visible light

Chloé Geoffroy[1], Romain Berraud-Pache[2], Nicolas Chéron[3], Isabelle McCort-Tranchepain[4], Julia Doria[1], Pierre Paoletti[1], and Laetitia Mony[1,*]

[1]Institut de Biologie de l'Ecole Normale Supérieure (IBENS), Ecole Normale Supérieure, Université PSL, CNRS, INSERM, F-75005 Paris, France

[2]Sorbonne Université, Laboratoire d'Archéologie Moléculaire et Structurale (LAMS), CNRS UMR 8220, Paris 75005, France

[3]PASTEUR, Département de chimie, École normale supérieure, Université PSL, Sorbonne Université, CNRS, 75005 Paris, France

[4]Université Paris Cité, Laboratoire de Chimie et Biochimie Pharmacologiques et Toxicologiques, CNRS UMR8601, 75006 Paris, France

*Corresponding author: Dr Laetitia Mony
*To whom correspondence should be addressed (Dr. Laetitia Mony) E-mail: laetitia.mony@ens.psl.eu

Table of Contents

Chemistry

Spectra S1A−K: ^{1}H, Cosy,^{13}C NMR, HSQC, HMBC and HPLC-MS spectra of **OptoNAM-1** .... S3−S8

Spectra S2A,B: ^{1}H NMR and HPLC-MS spectra of **OptoNAM-2** S9

Spectra S3A−P: ^{1}H, Cosy,^{13}C NMR, HSQC , HMBC and HPLC-MS spectra of **OptoNAM-3** .... S10−S17

Spectra S4A,B: ^{1}H NMR and HPLC-MS spectra of **OptoNAM**-4 .... S18

Photochemical and biological characterizations of OptoNAMs

Text S1: Photochemical and biological characterizations of OptoNAM-1, -2 and -4 activity S19

Text S2: Docking and molecular dynamics simulations S20

Figure S1: Photochemical properties of OptoNAM-1 to -4 and their photodependent activity at GluN1/GluN2B receptors .... S21−S22

Figure S2: Decreased pKa of OptoNAM-1 and -2 compared to their parent compounds are likely responsible for their decreased activity .... S23−S24

Figure S3: Additional data relative to Figure 3 .... S25−S26

Figure S4: OptoNAM-3 decreases NMDA-induced neuronal death in a photodependent manner S27

Figure S5: OptoNAM-3 photomodulates Xenopus tadpole locomotion in vivo: protocol and tadpole locomotion normalized to baseline locomotion. Additional data relative to Figure 6 .... S28

Figure S6: OptoNAM-3 photochemical properties in different solvents S29

Figure S7: Evolution of *trans*-OptoNAM-3 conformation in its binding-site and in water .... S30−S31

Figure S8: Additional data relative to Figure 7.. S32

Figure S9: Overlap between crystallographic and docked poses of ifenprodil and overlap between crystallographic pose of ifenprodil and docked pose of *trans*-OptoNAM-3 .... S33

Table S1: Summary of the IC_{50}s of OptoNAMs in the dark and UV compared to the activity of their parent compounds S34

Table S2: Computed vertical energies and oscillator strengths of 11 snapshots of free *trans*-OptoNAM-3 in implicit water for the 2 first visible transitions using the B2PLYP functional S35

Table S3: Computed vertical energies and oscillator strengths of 11 snapshots of bound *trans*-OptoNAM-3 inside the protein for the 2 rotamers and for the 2 first transitions using the B2PLYP functional S36

Table S4: Computed vertical energies and oscillator strengths of snapshot 0 of bound *trans*-OptoNAM-3 for the 2 rotamers and for the 1st transition (n → π*), inside the protein; without the protein without optimization, and without the protein after being optimized in vacuum S37

Supplementary Movie 1 (separate file) S38

Supplementary Movie 2 (separate file) S38

Supplementary Data 1 (separate file) S38

Supplementary Data 2 (separate file) S38

Supplementary Data 3 (separate file) S38

References S39

Spectra S1A,B

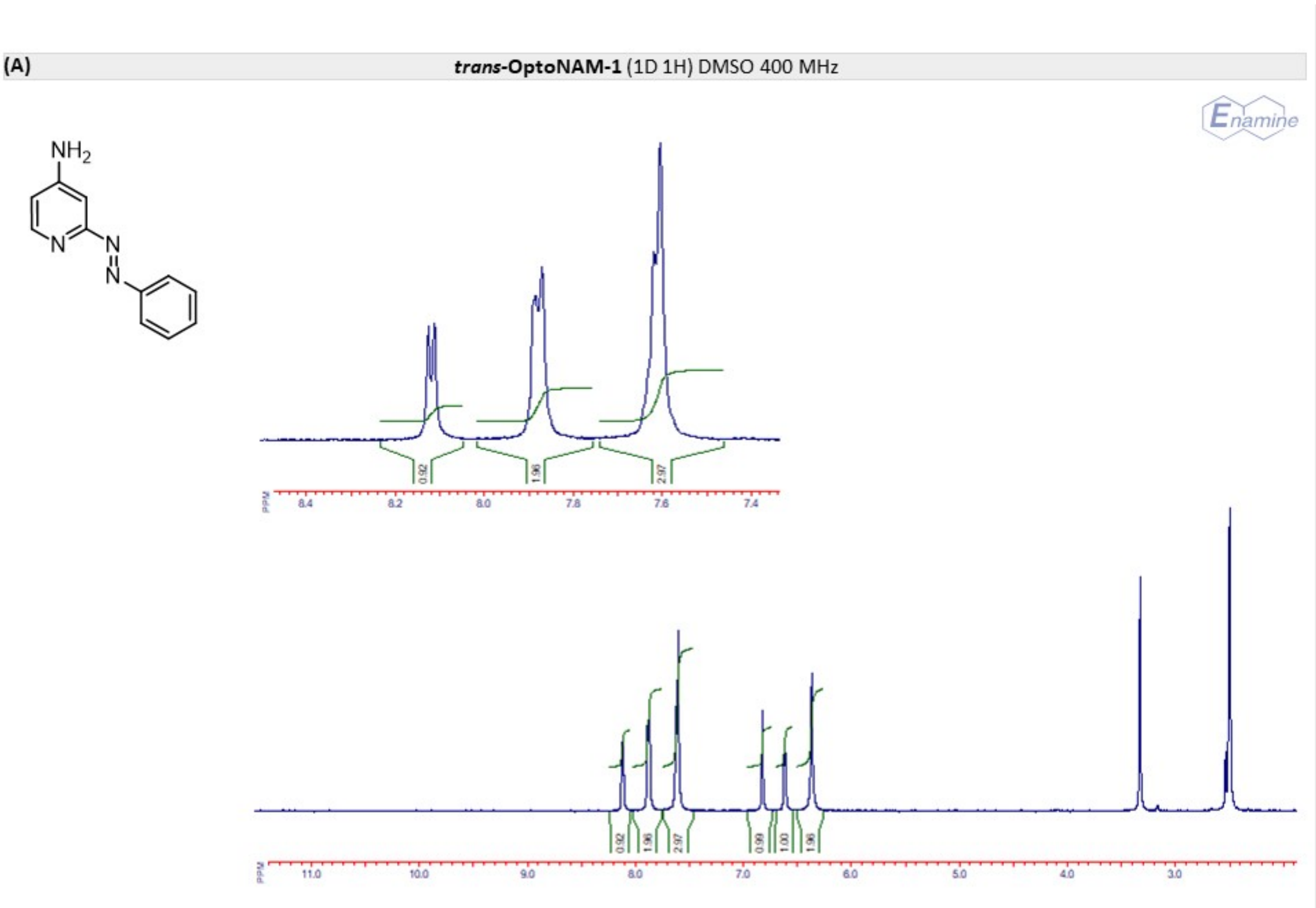

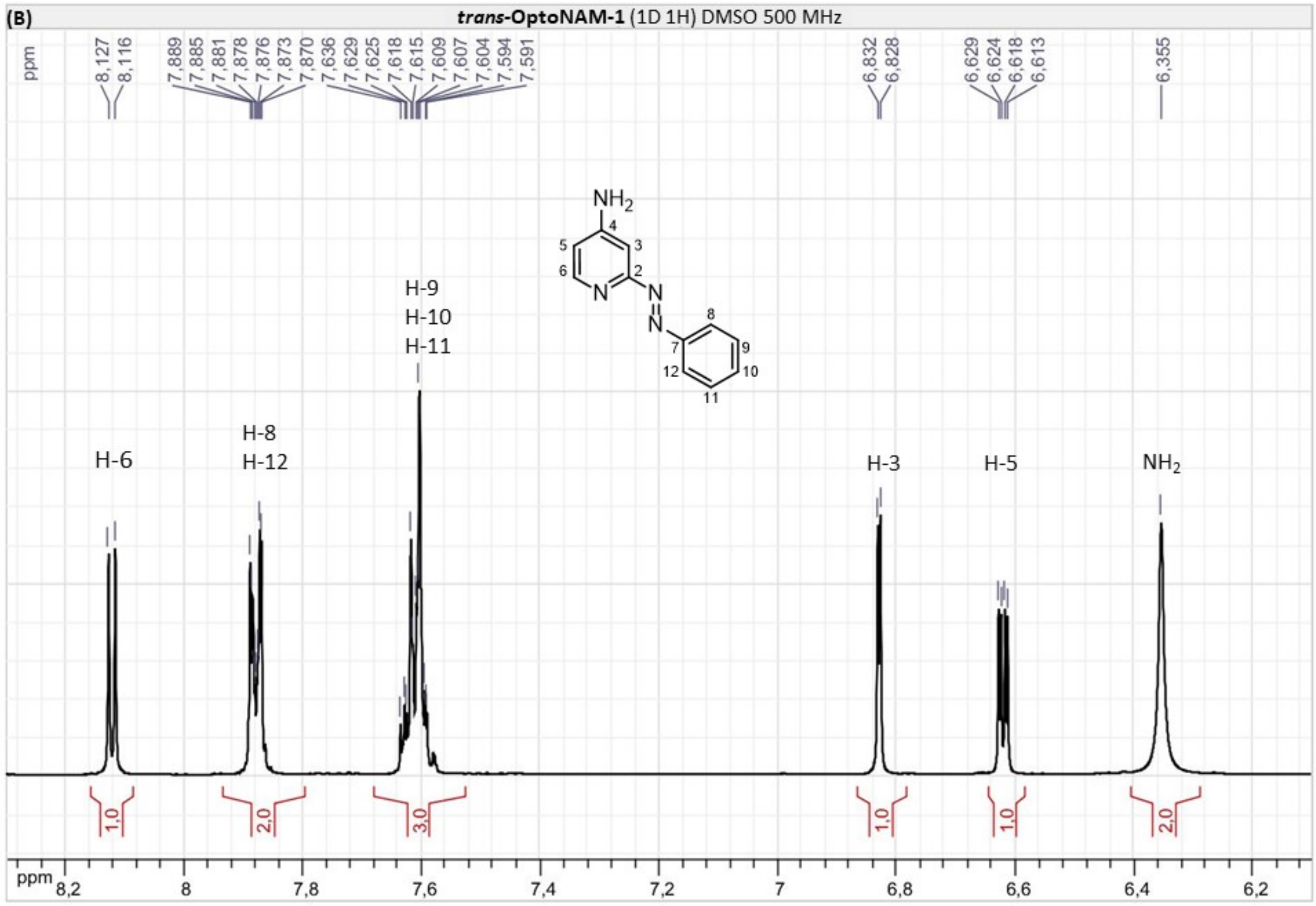

Spectra S1C,D

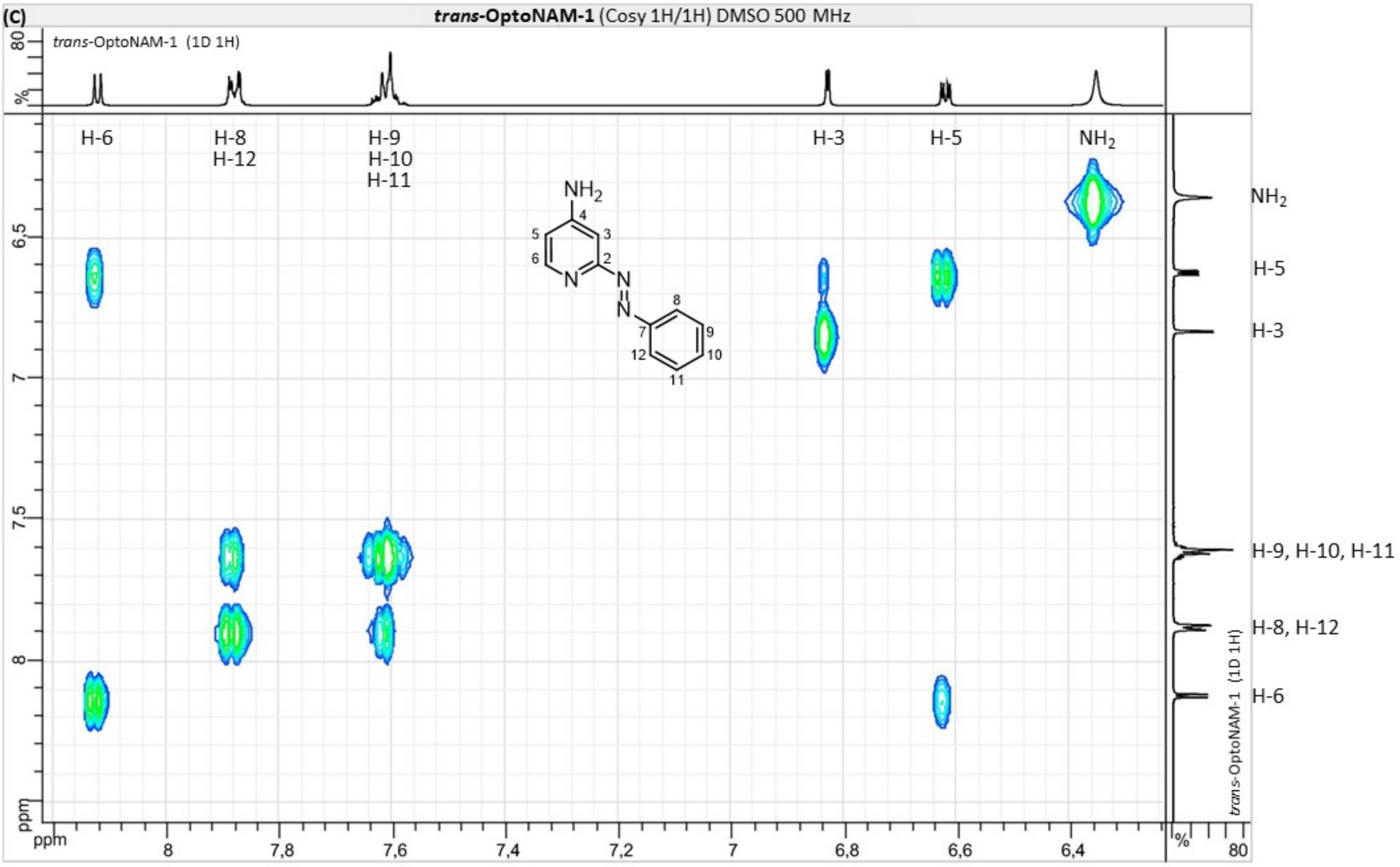

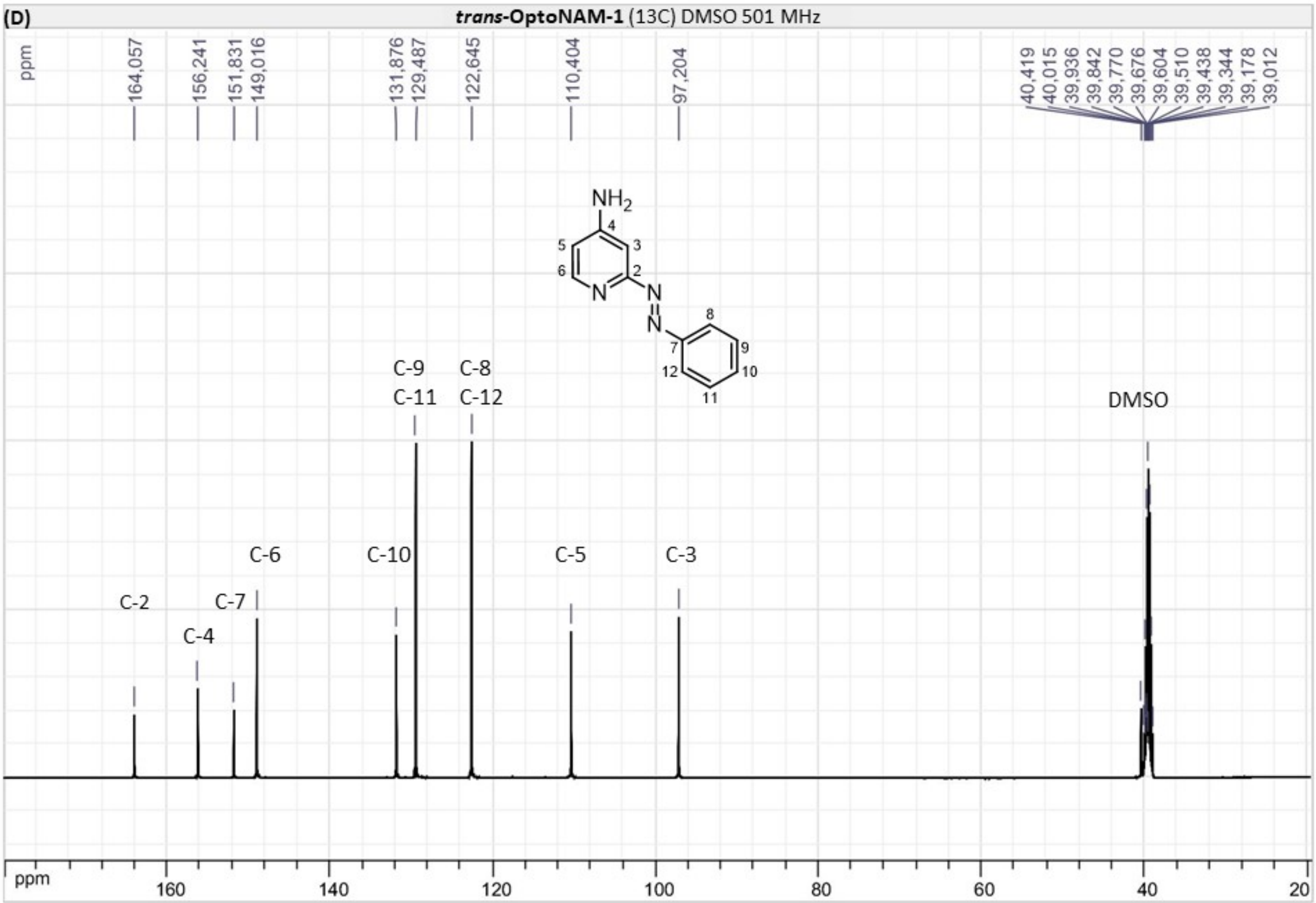

Spectra S1E,F

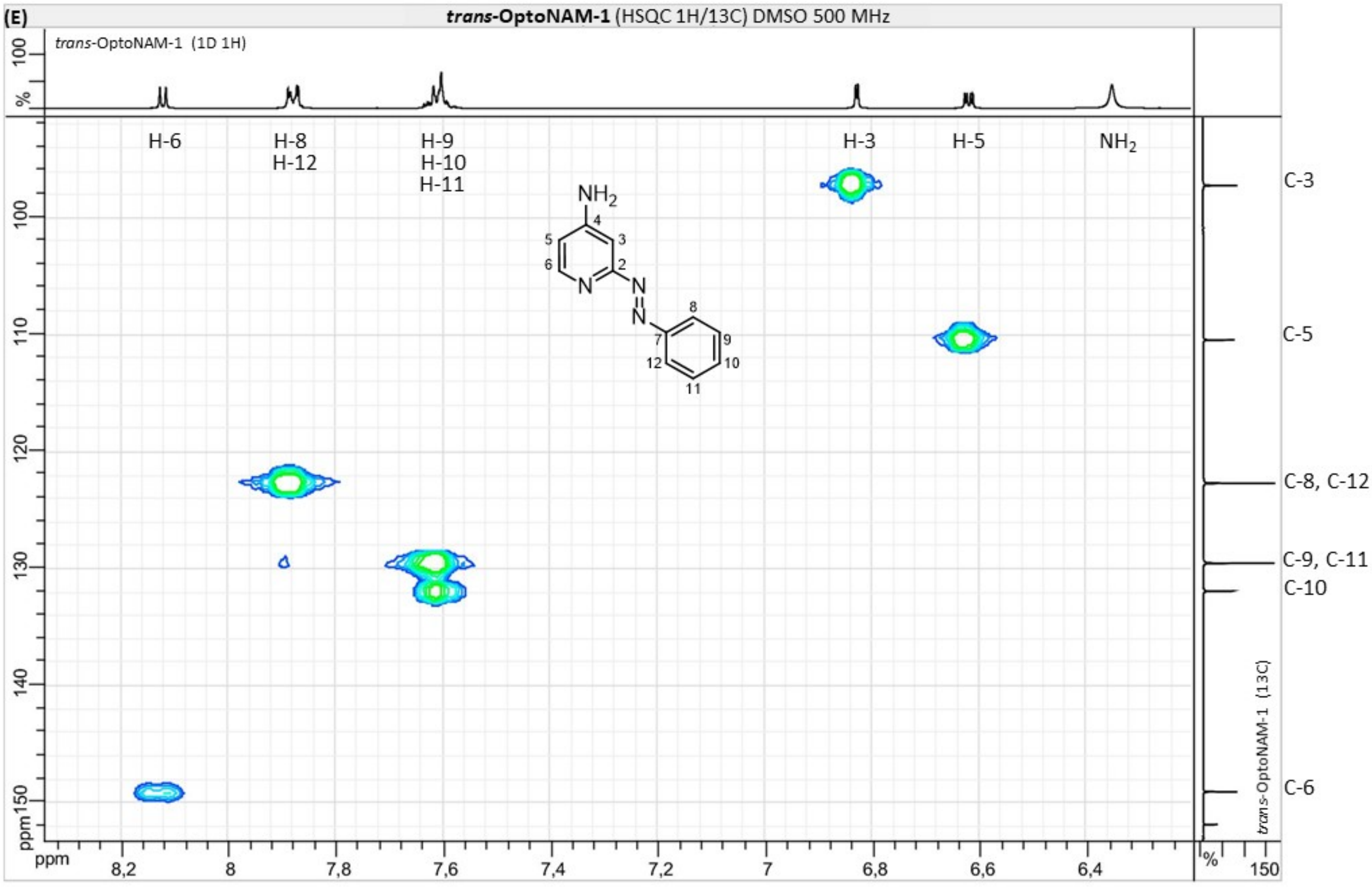

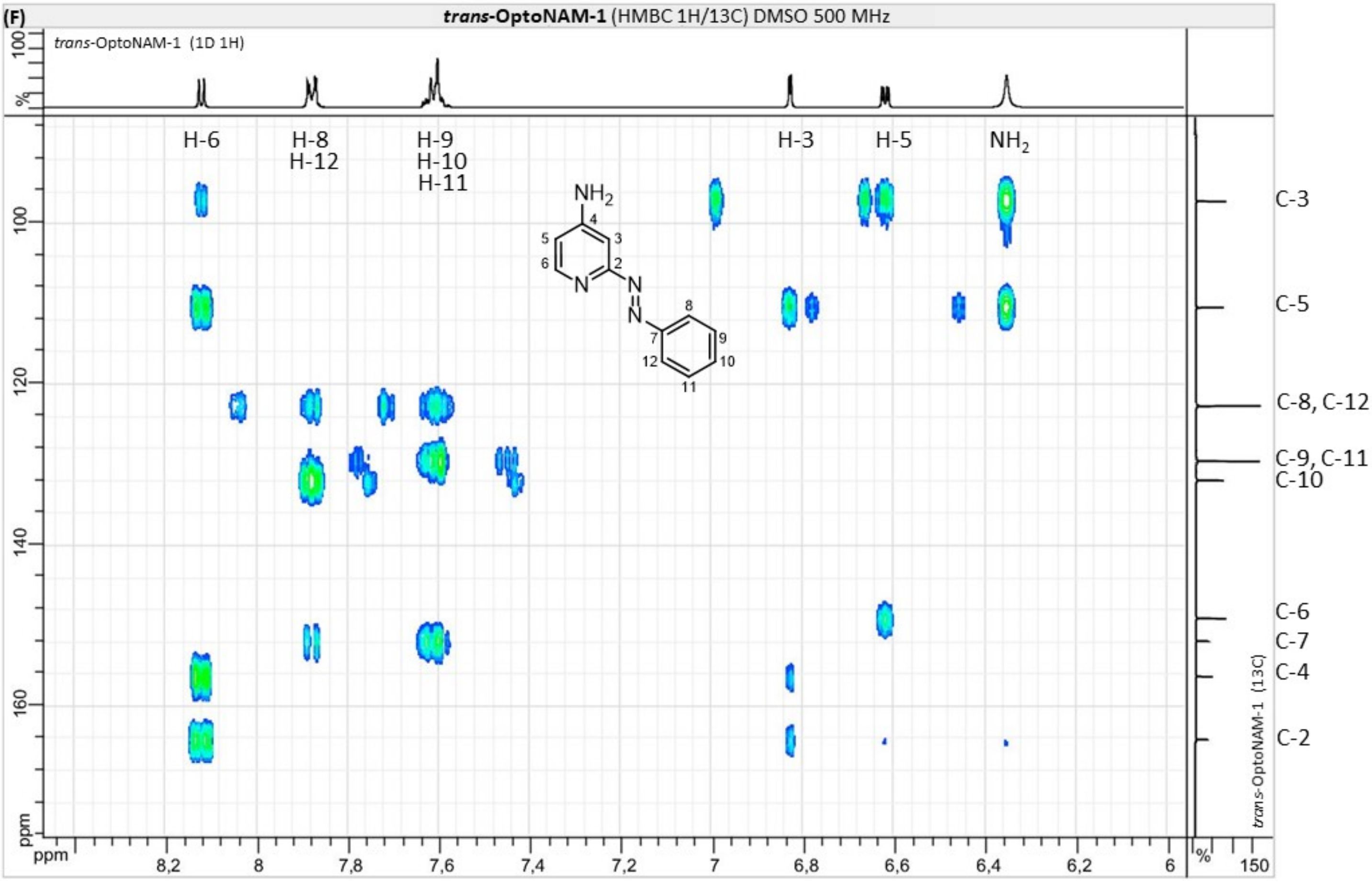

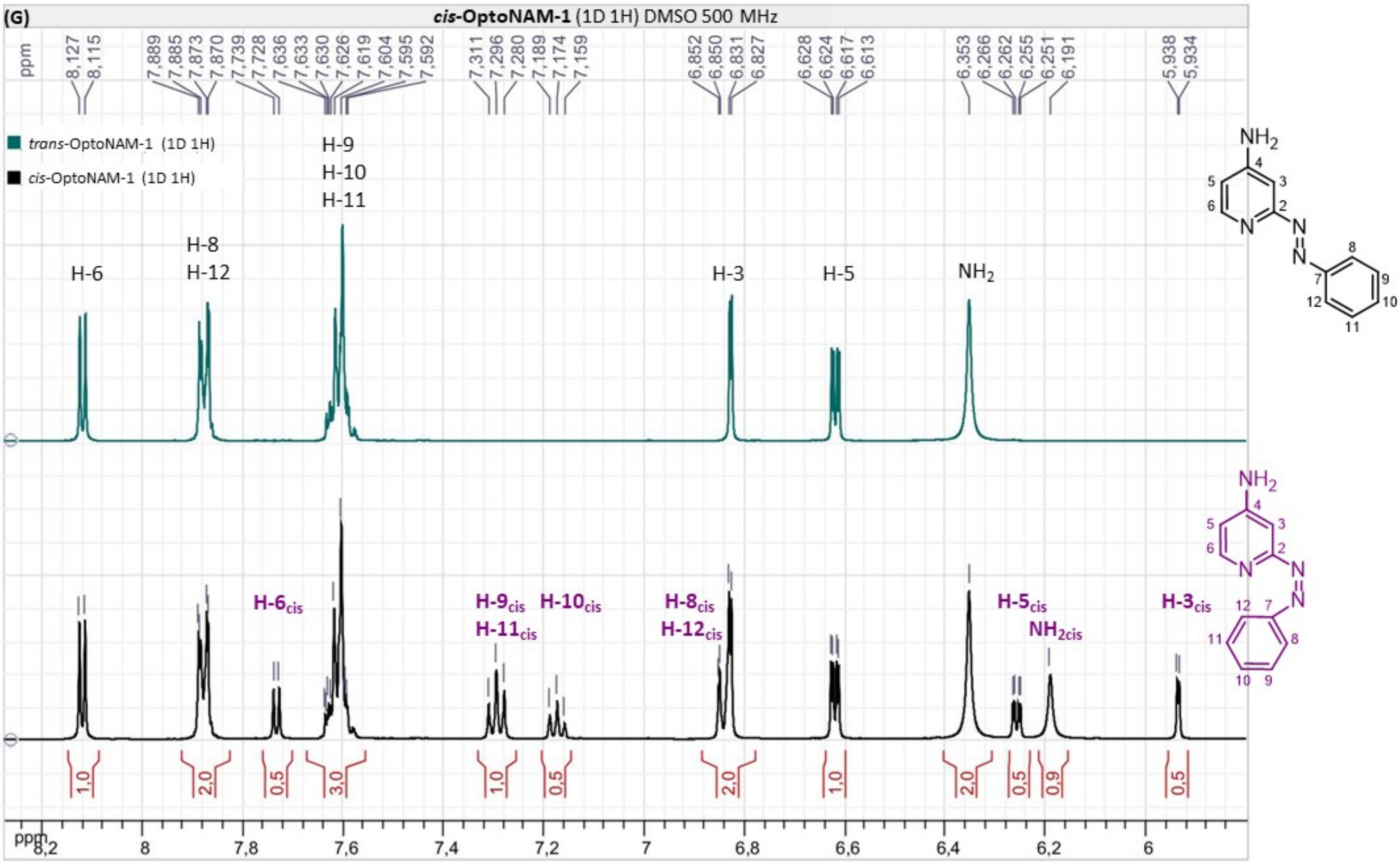

Upper panel: ^{1}H NMR spectrum of **OptoNAM-1** in the dark (100% *trans*). Lower panel: ^{1}H NMR spectrum of **OptoNAM-1** 365 nm PSS. Percentage of *cis* and *trans* isomers PSSs was calculated from the integration of the peaks at 8.12 ppm and 7.73 ppm.

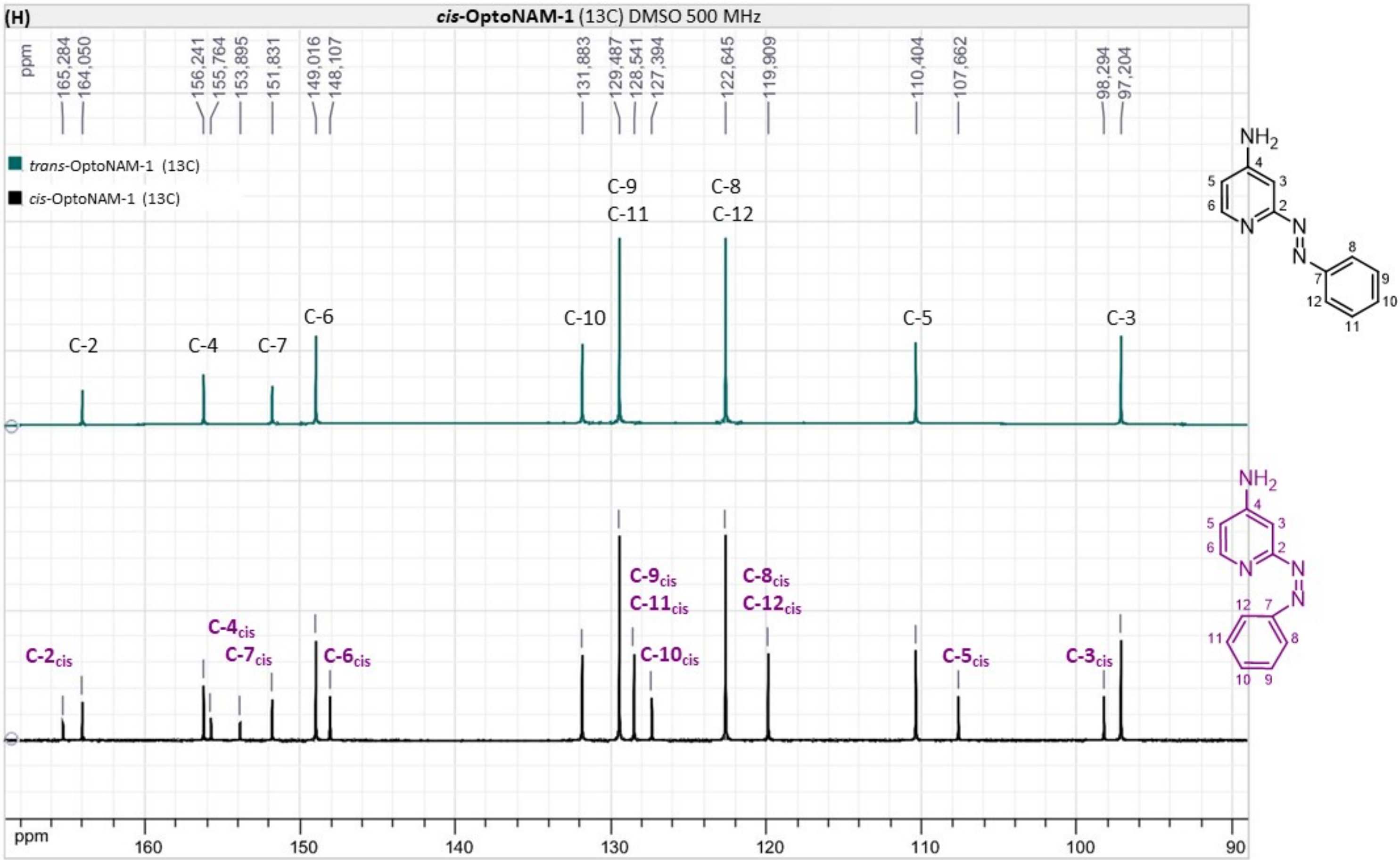

Spectra S1I,J

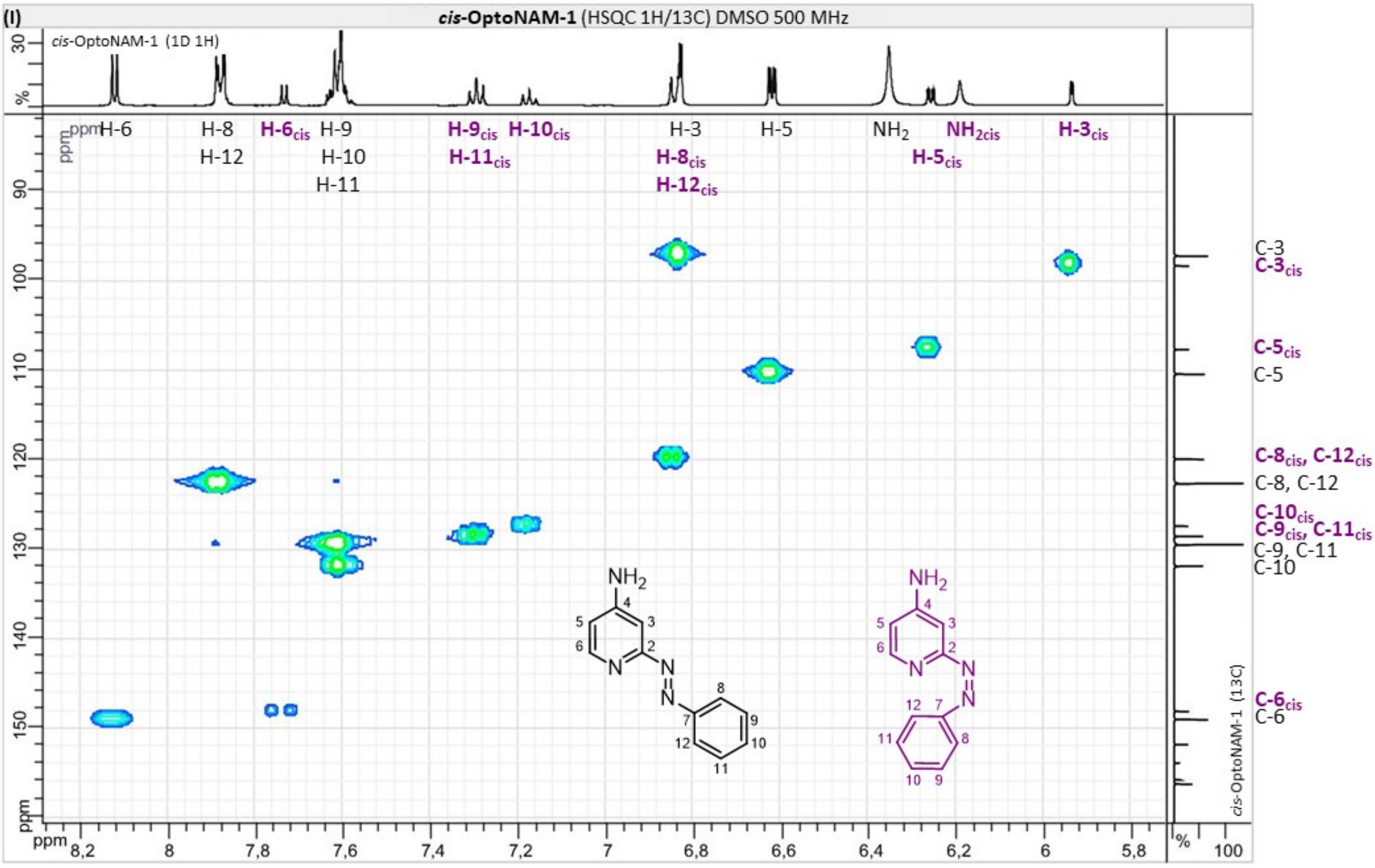

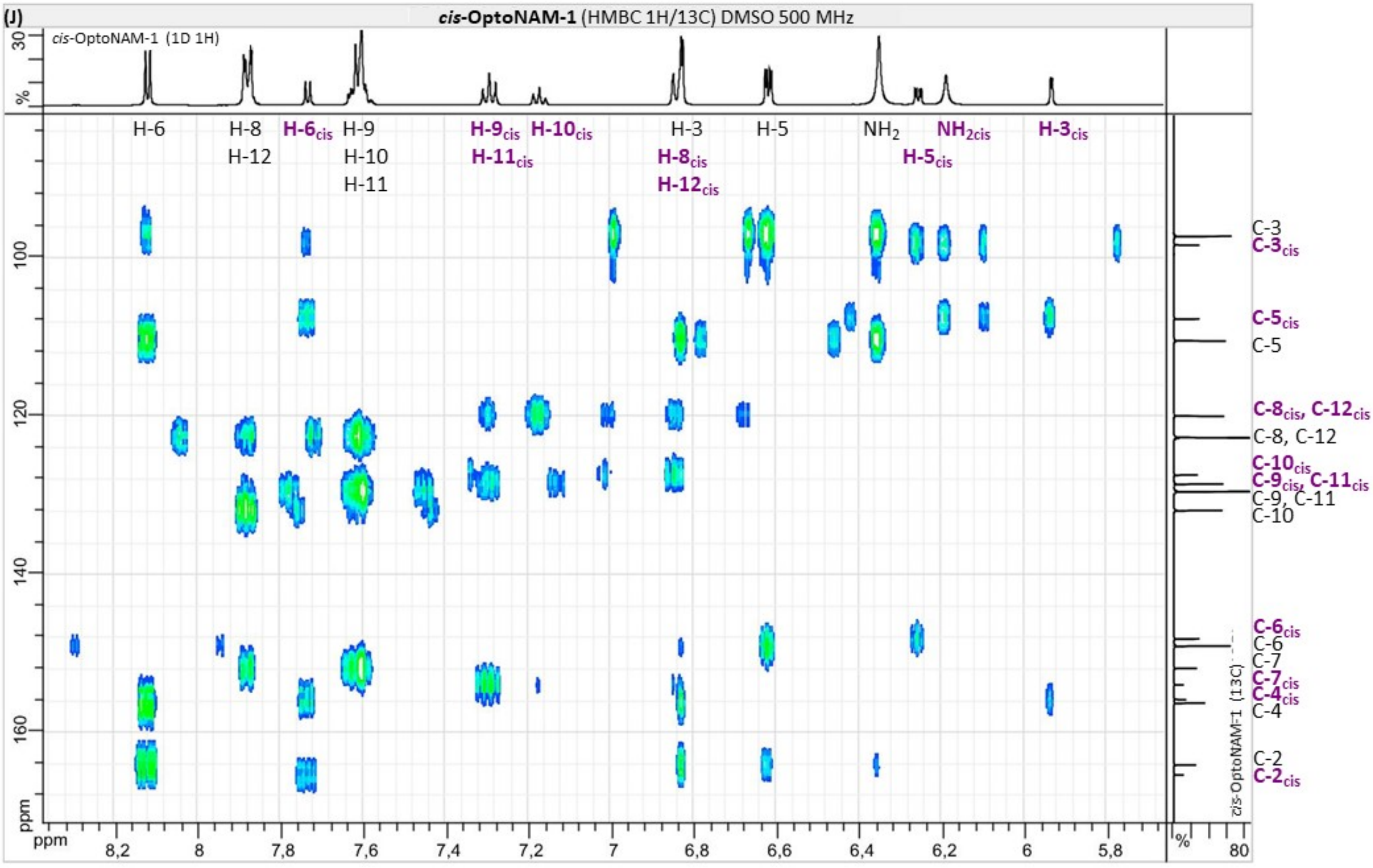

(K) **HPLC-MS of OptoNAM-1**

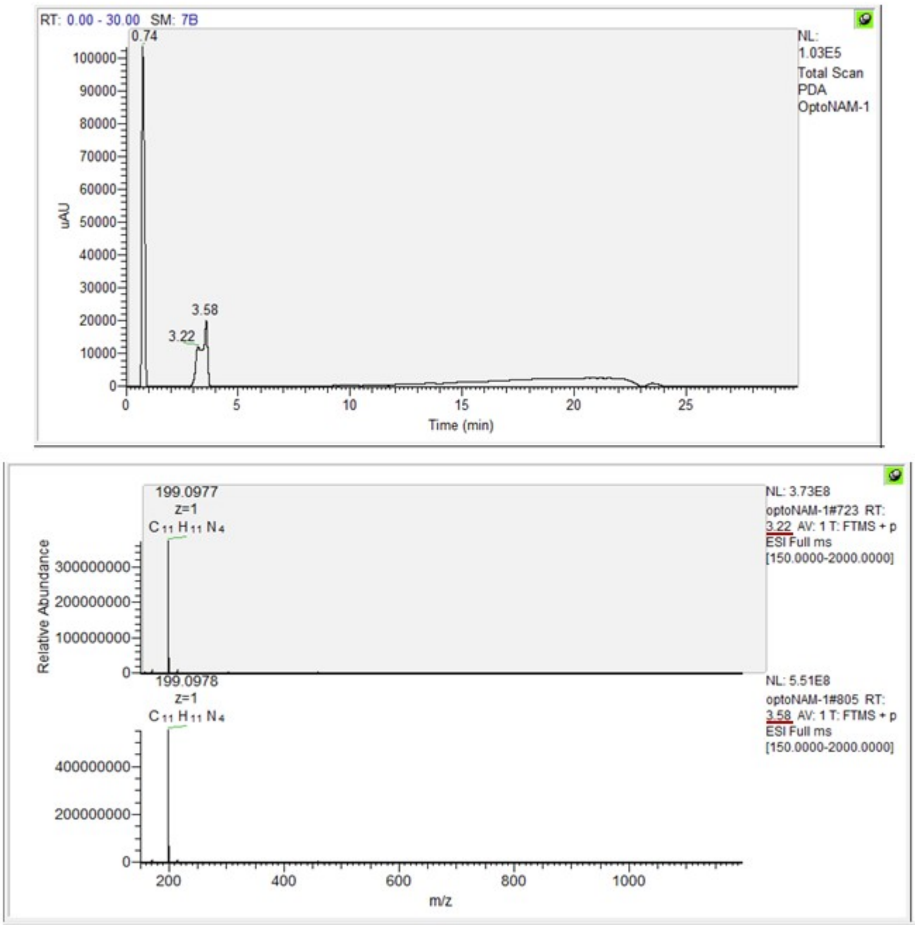

The peaks at retention time 3.22 min and 3.58 min have the same mass profile.

Spectra S2A,B

(A) *trans*-**OptoNAM-2** (1D 1H) DMSO 400 MHz

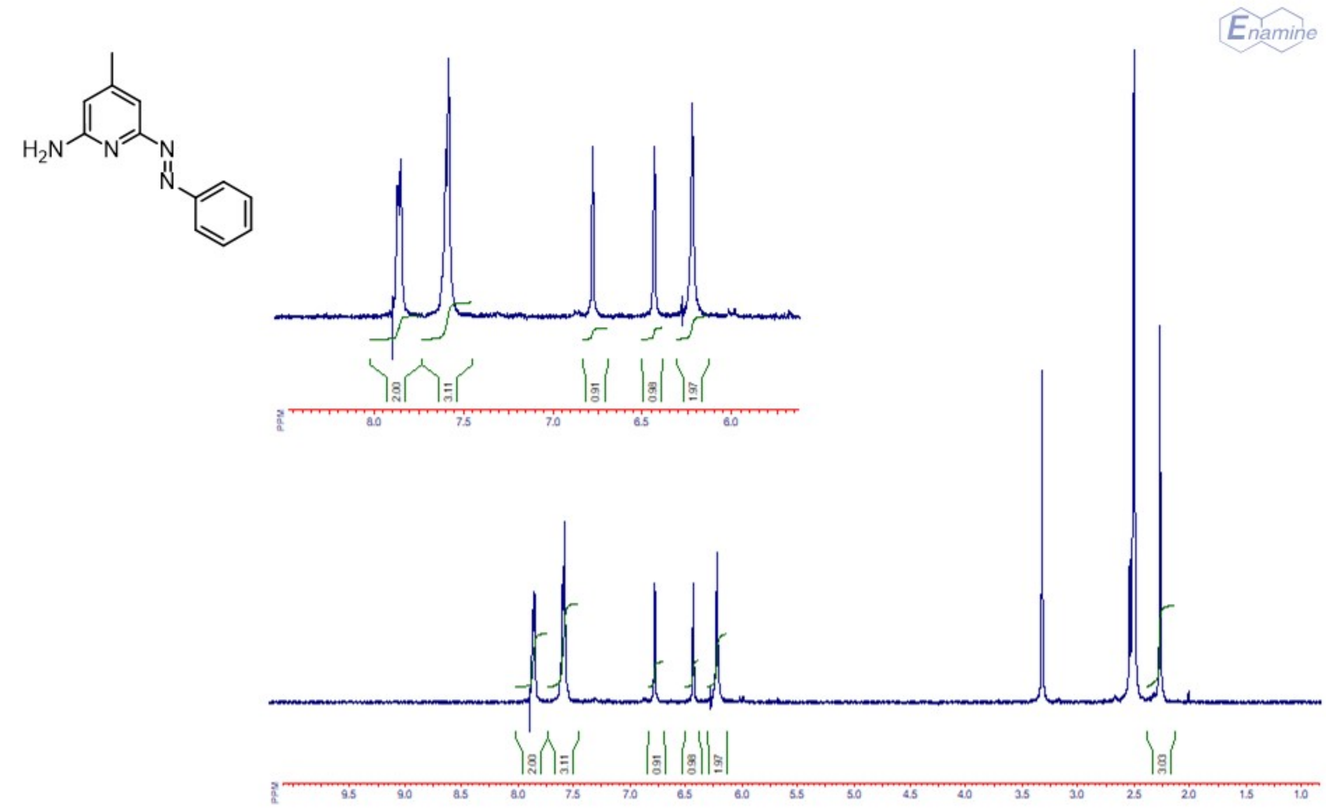

(B) **HPLC-MS of OptoNAM-2**

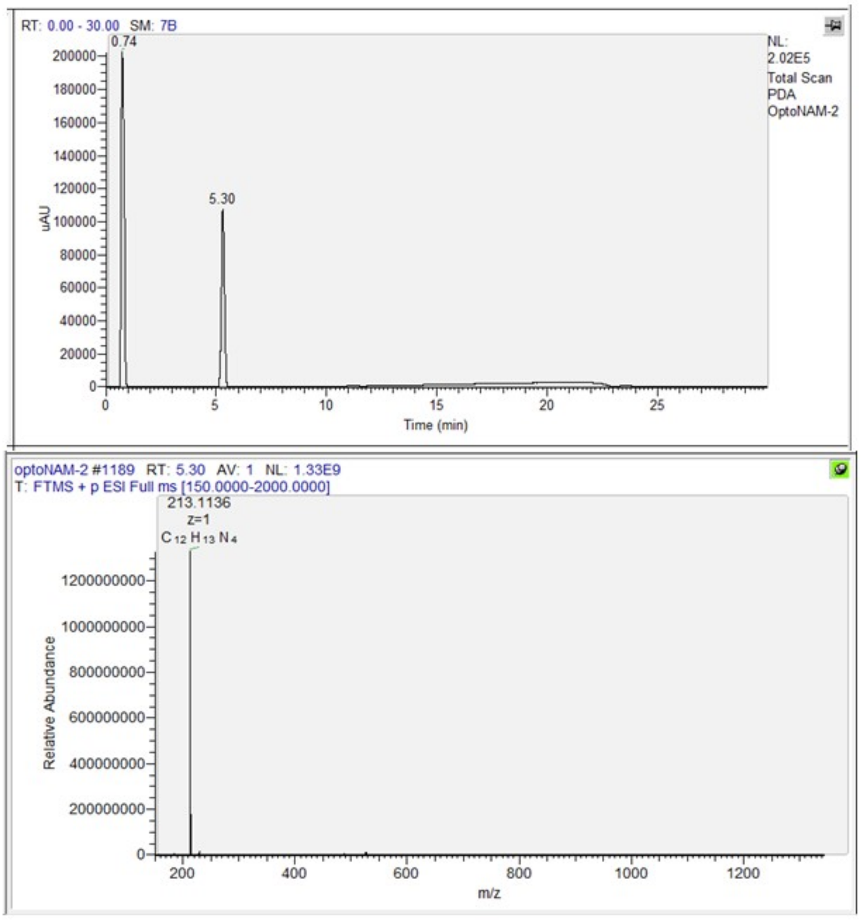

Spectra S3A,B

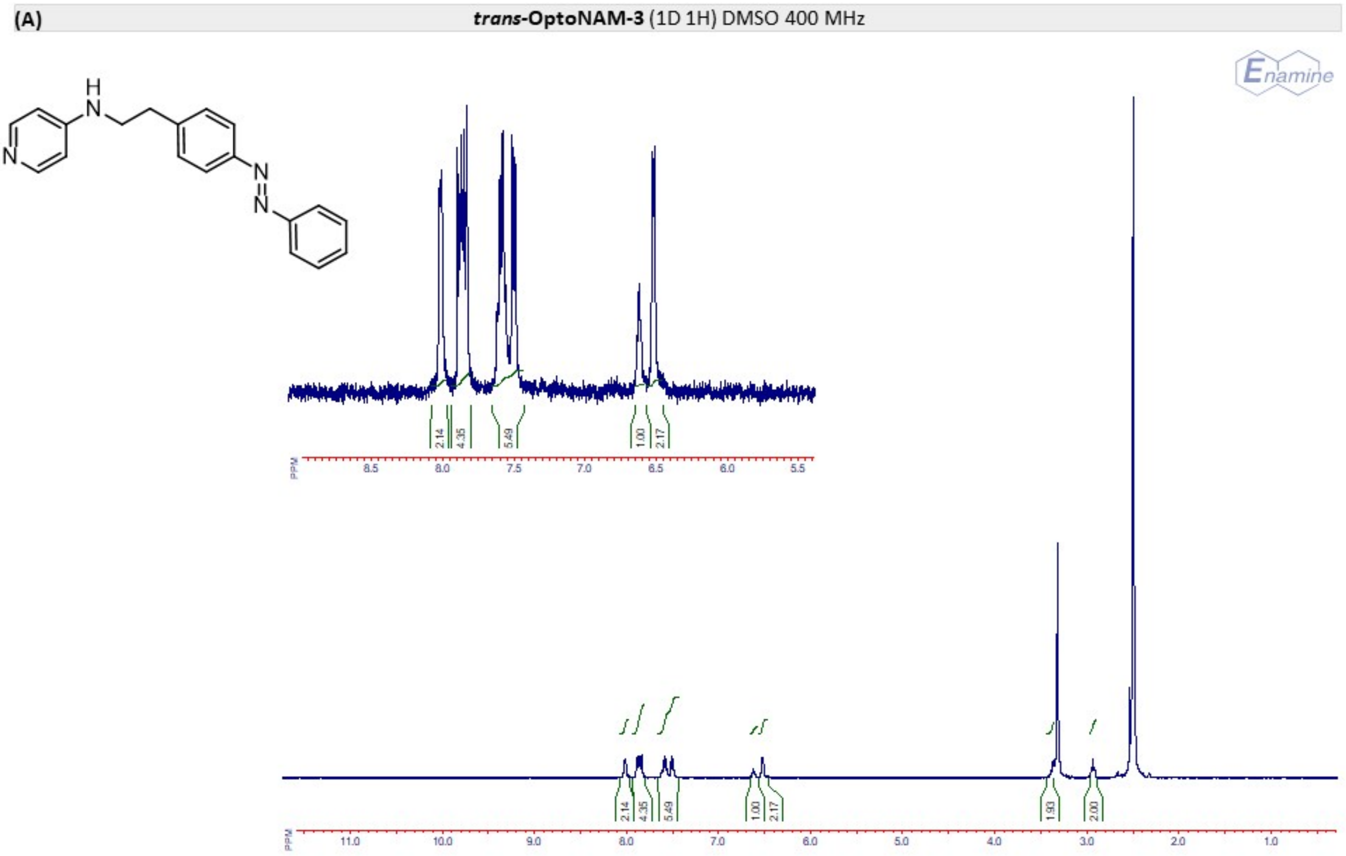

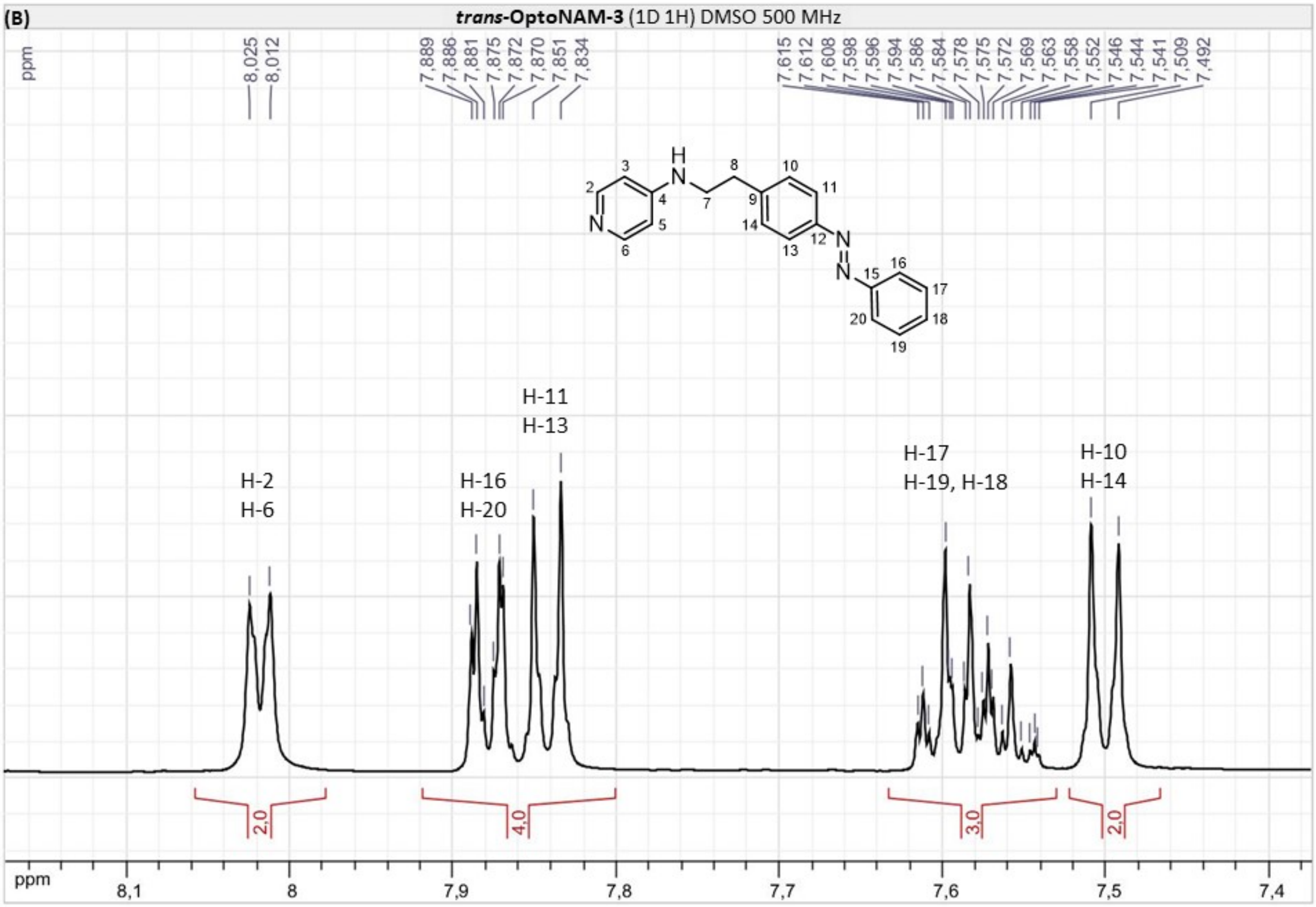

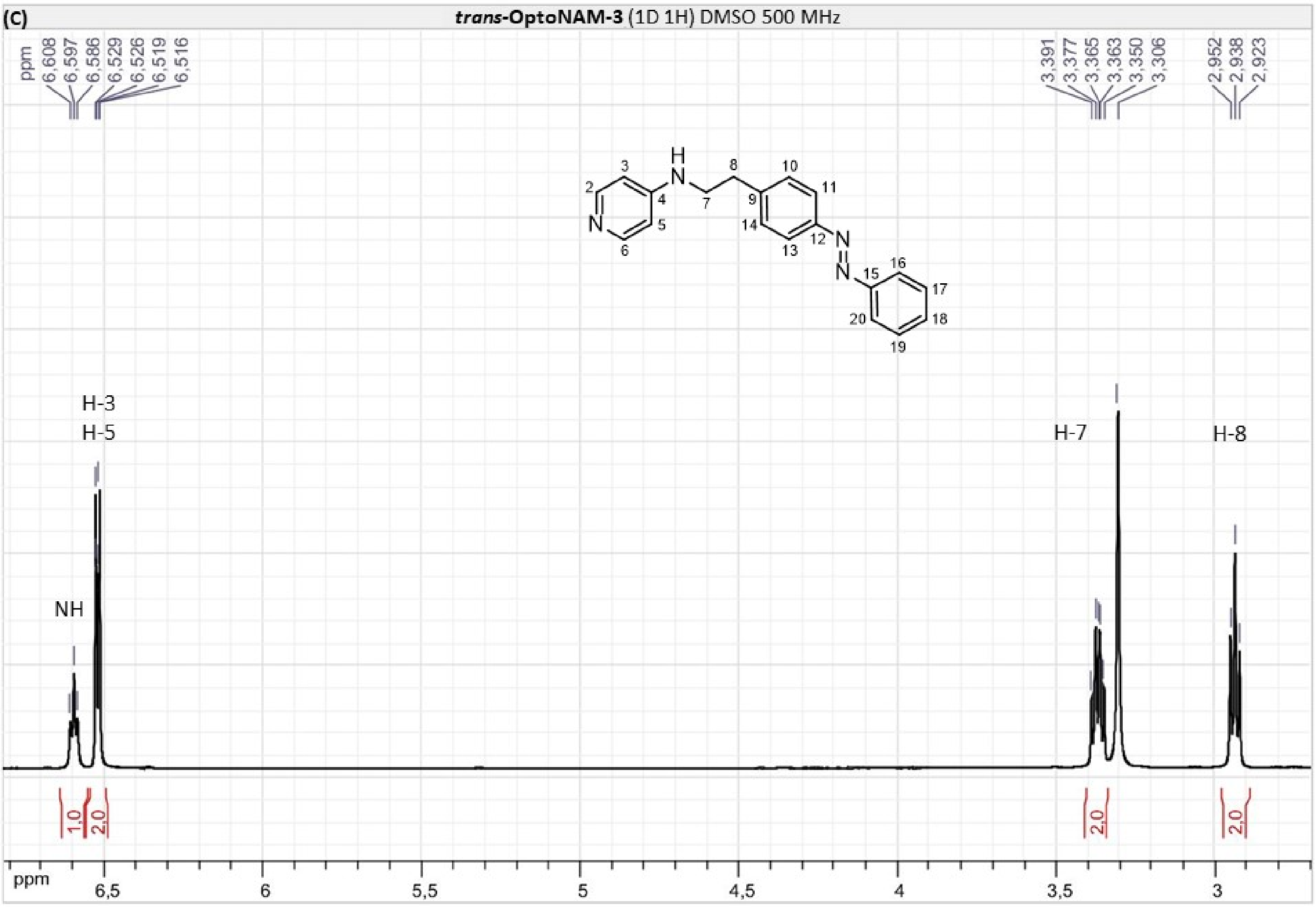
(C)
trans-OptoNAM-3 (1D 1H) DMSO 500 MHz
ppm 6,608 6,597 6,586 6,529 6,526 6,519 6,516
3,391 3,377 3,365 3,363 3,350 3,306
2,952 2,938 2,923
H-3
H-5
NH
H-7
H-8
1,0
2,0
2,0
2,0
ppm
6,5
6
5,5
5
4,5
4
3,5
3

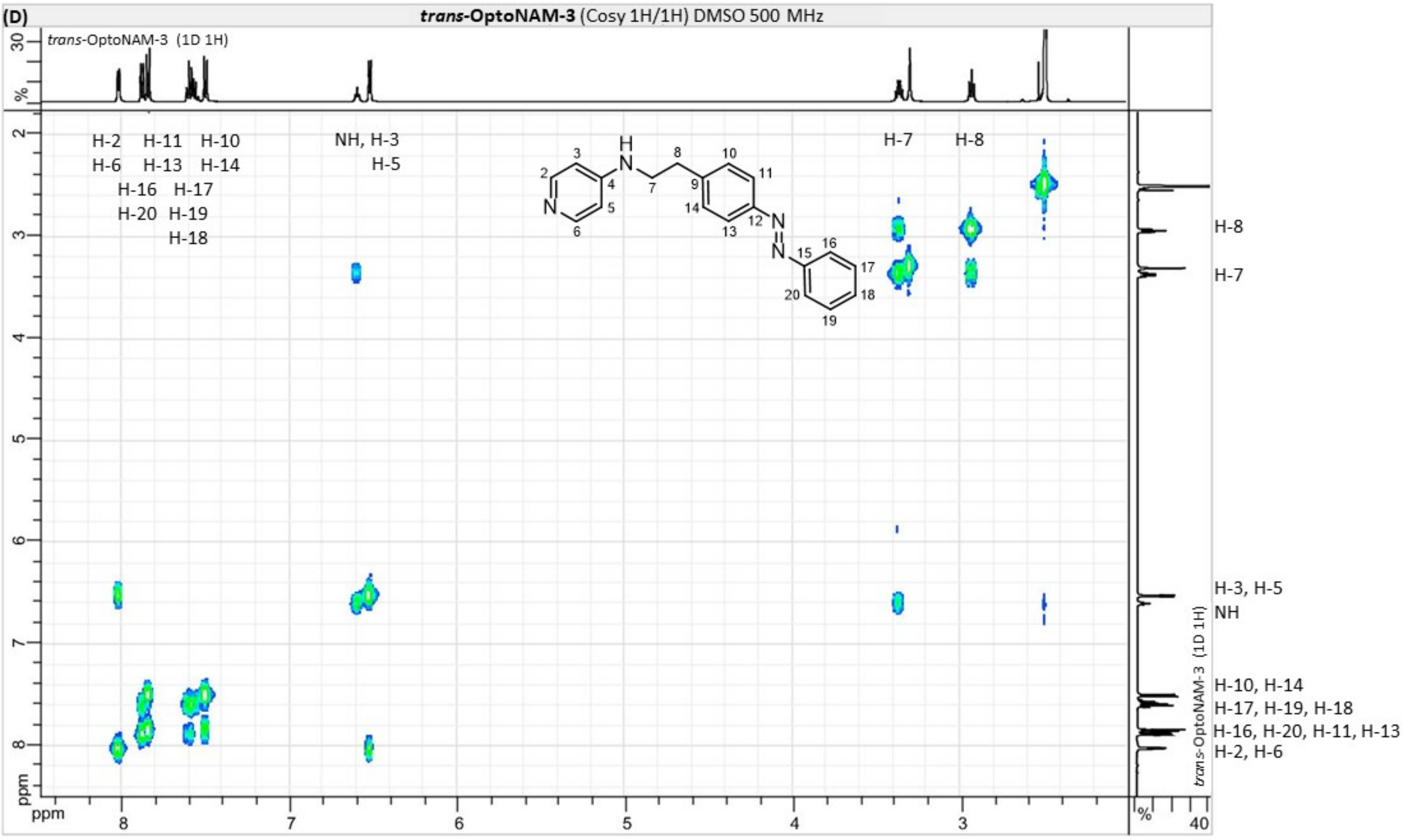
(D)
trans-OptoNAM-3 (Cosy 1H/1H) DMSO 500 MHz
trans-OptoNAM-3 (1D 1H)
H-2 H-6
H-11 H-13 H-16 H-20
H-10 H-14 H-17 H-19 H-18
NH, H-3 H-5
H-7
H-8
H-3, H-5
NH
H-10, H-14
H-17, H-19, H-18
H-16, H-20, H-11, H-13
H-2, H-6
ppm
8
7
6
5
4
3

Spectra S3E,F

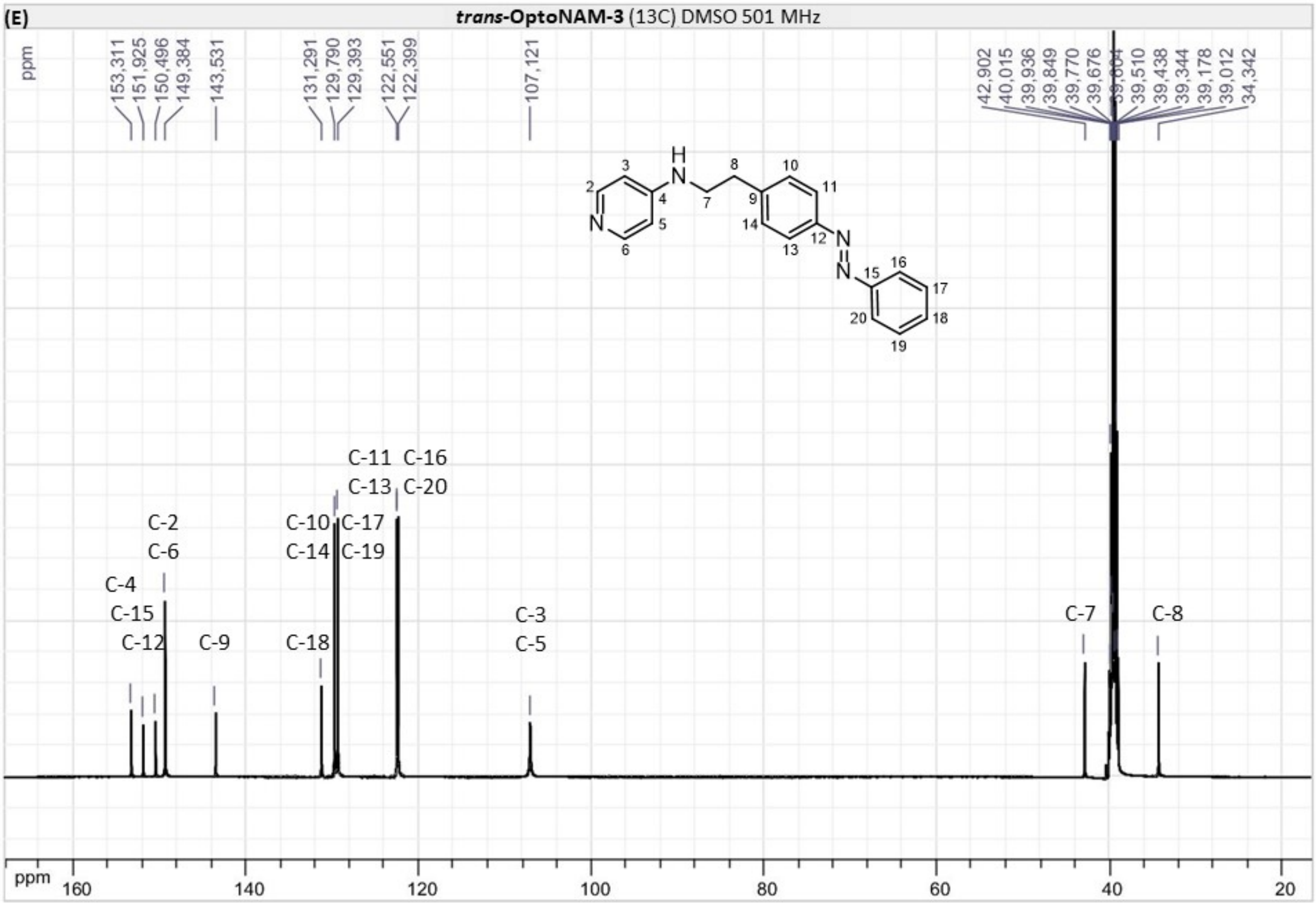

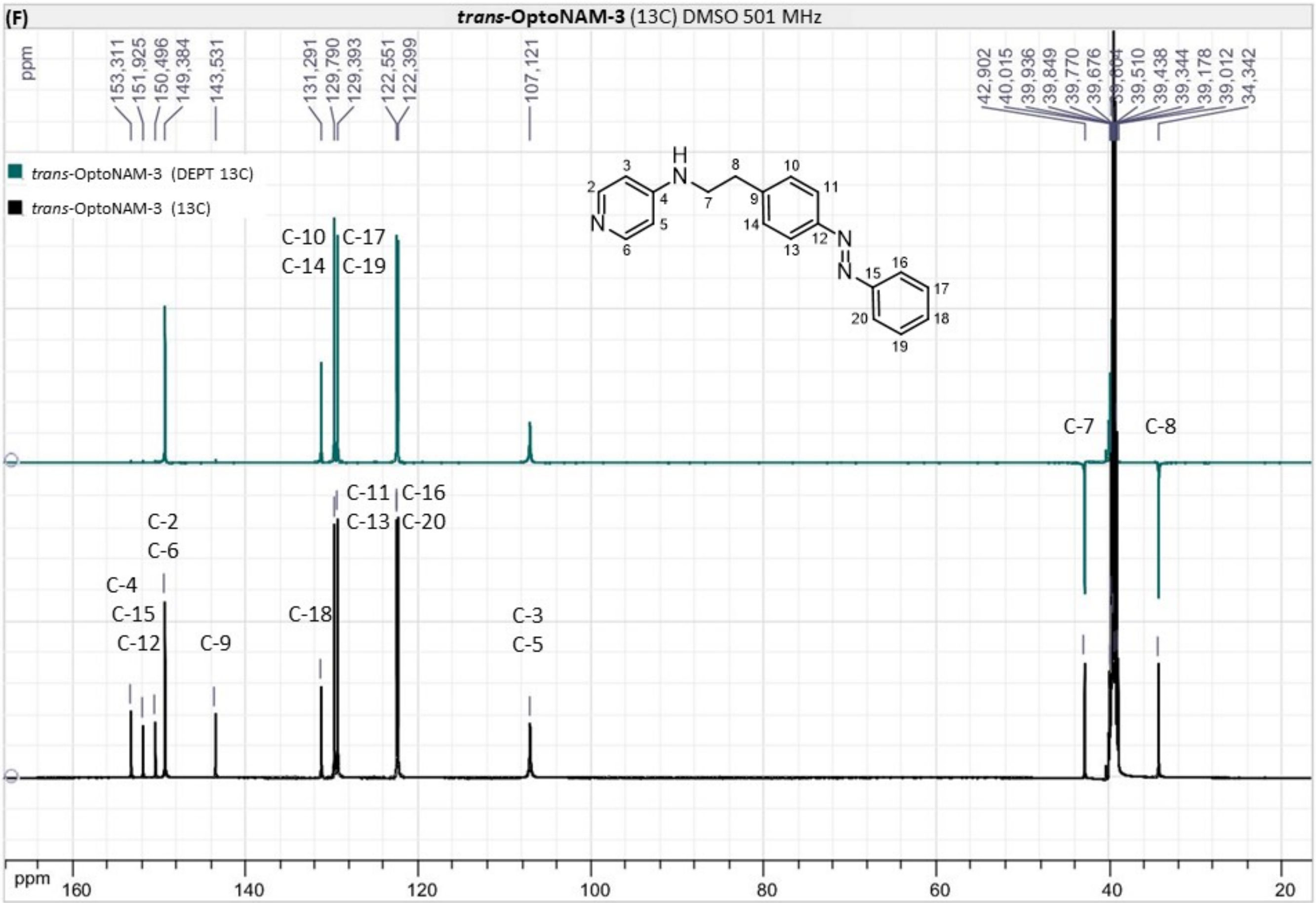

Spectra S3G,H

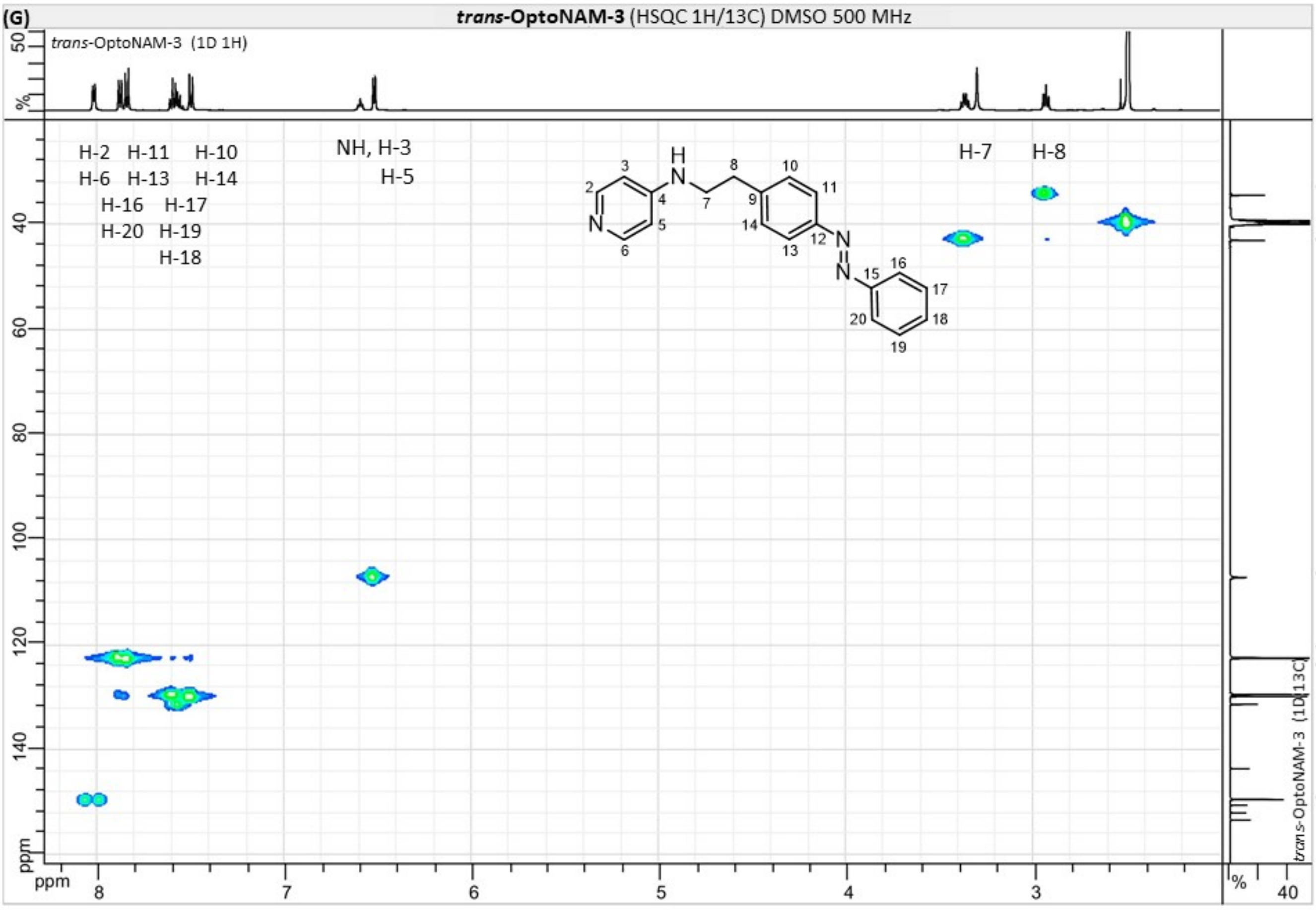

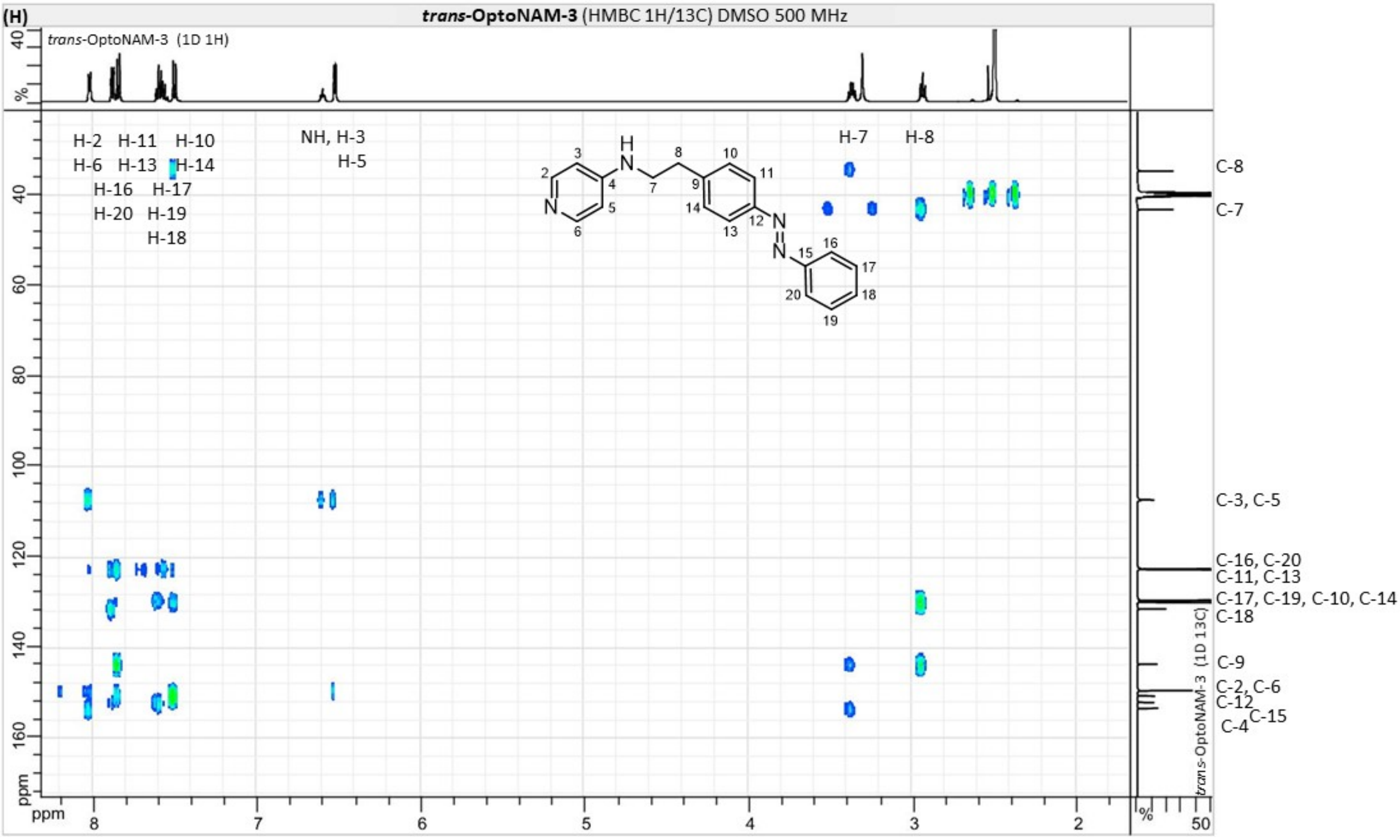

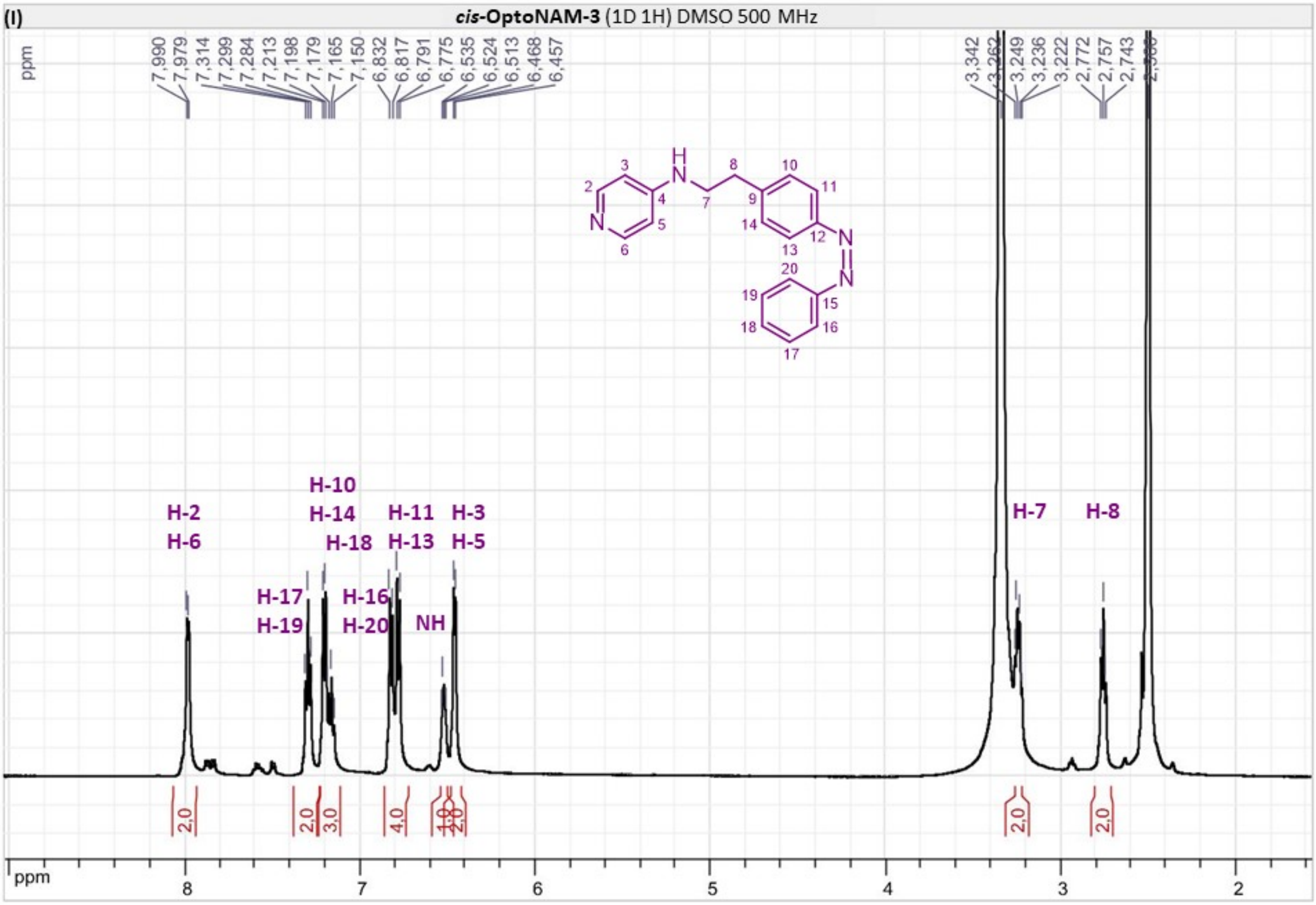
(I)
cis-OptoNAM-3 (1D 1H) DMSO 500 MHz
7,990
7,979
7,314
7,299
7,284
7,213
7,198
7,179
7,165
7,150
6,832
6,817
6,791
6,775
6,535
6,524
6,513
6,468
6,457
3,342
3,262
3,249
3,236
3,222
2,772
2,757
2,743
H-2
H-6
H-17
H-19
H-10
H-14
H-18
H-16
H-20
H-11
H-13
NH
H-3
H-5
H-7
H-8
2,0
2,0
3,0
4,0
1,0
2,0
2,0
2,0
ppm
8
7
6
5
4
3
2

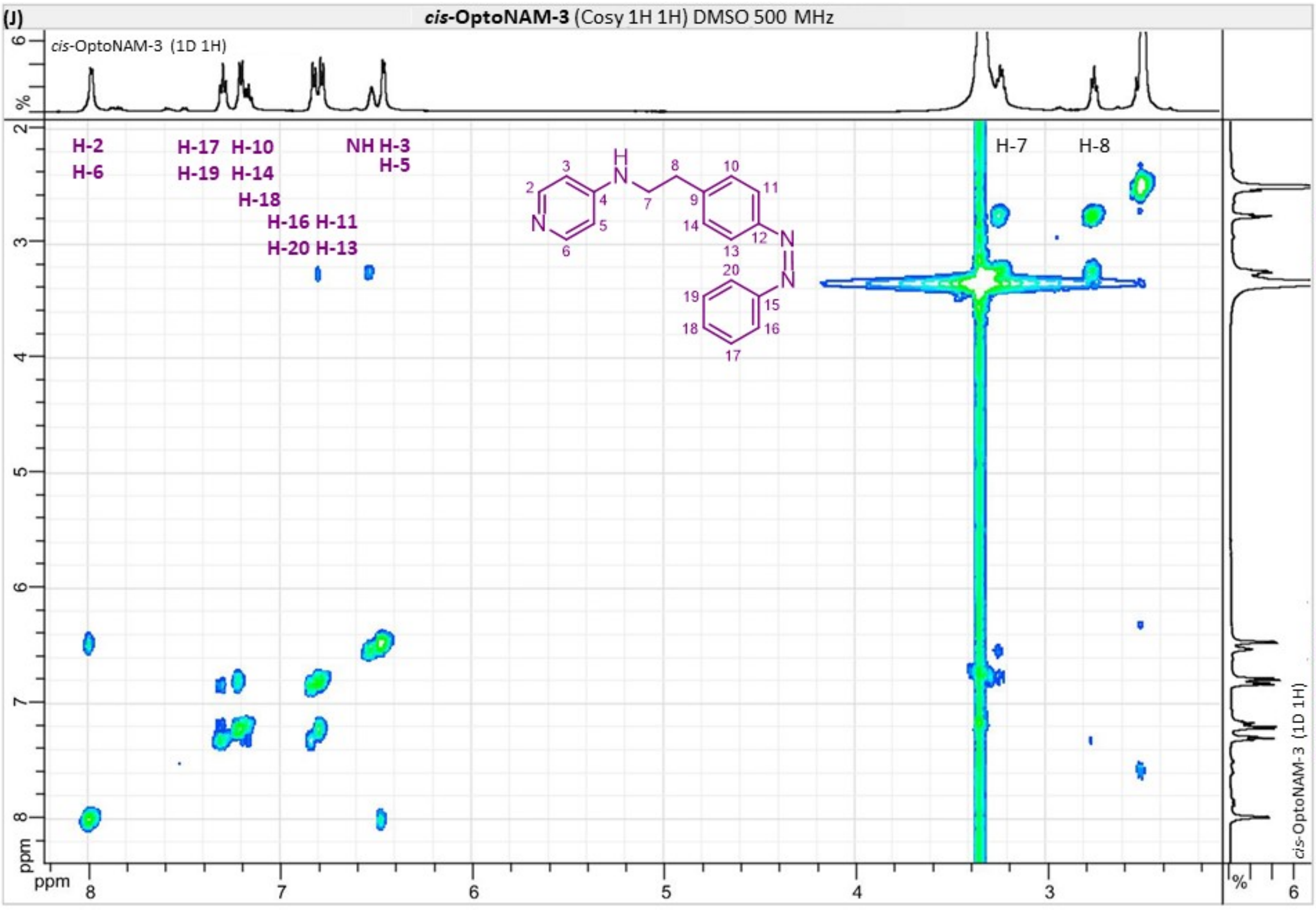
(J)
cis-OptoNAM-3 (Cosy 1H 1H) DMSO 500 MHz
cis-OptoNAM-3 (1D 1H)
H-2
H-6
H-17
H-19
H-10
H-14
H-18
H-16
H-20
H-11
H-13
NH
H-3
H-5
H-7
H-8
H-3, H-5
NH
H-11, H-13
H-16, H-20
H-18
H-10, H-14
H-17, H-19
H-2, H-6
ppm
8
7
6
5
4
3

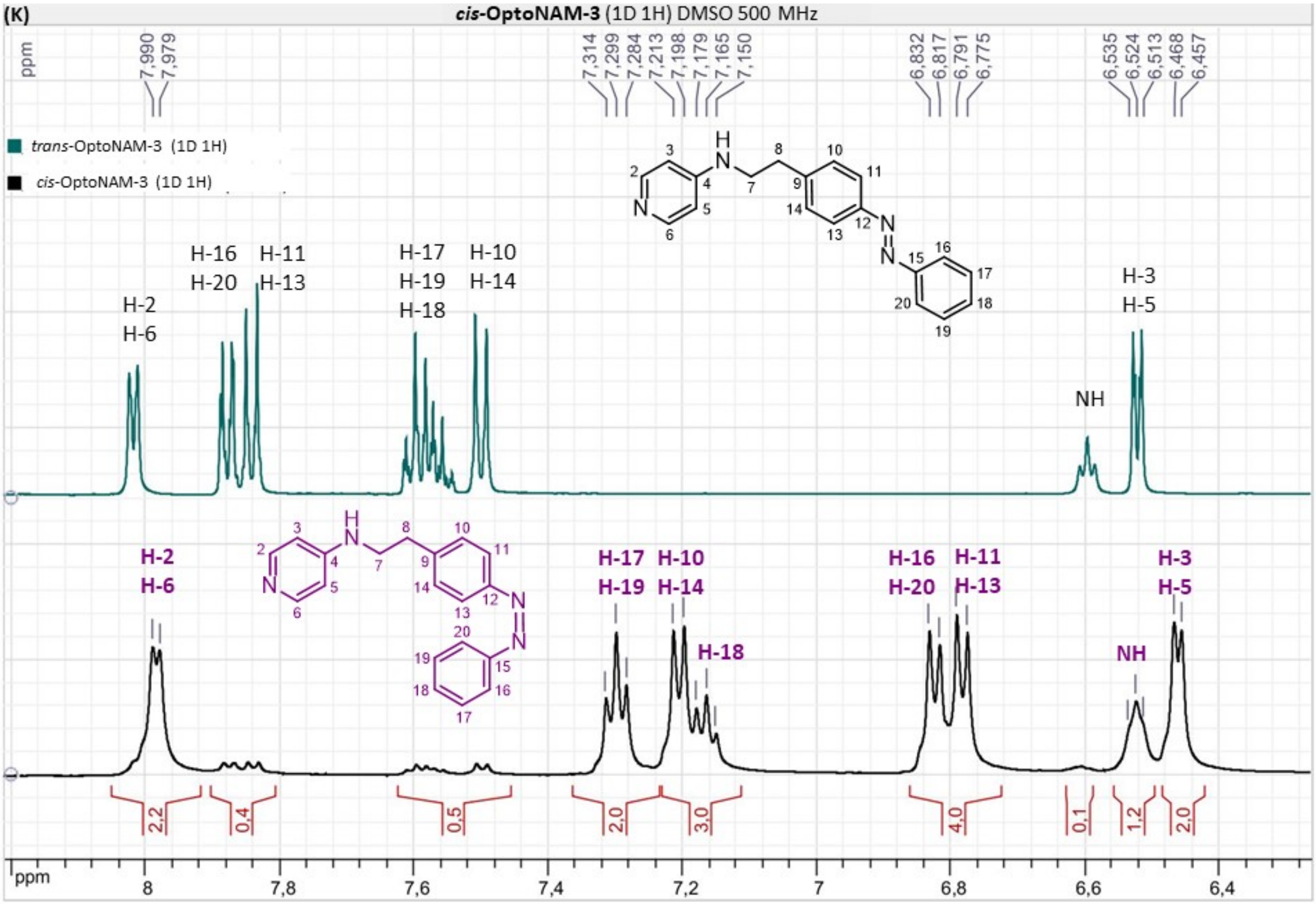

Upper panel: ^{1}H NMR spectrum of **OptoNAM-3** in the dark (100% *trans*). Lower panel: ^{1}H NMR spectrum of **OptoNAM-3** 365 nm PSS. Percentage of *cis* and *trans* isomers PSSs was calculated from the integration of the peaks at 6.80 ppm and 7.98 ppm.

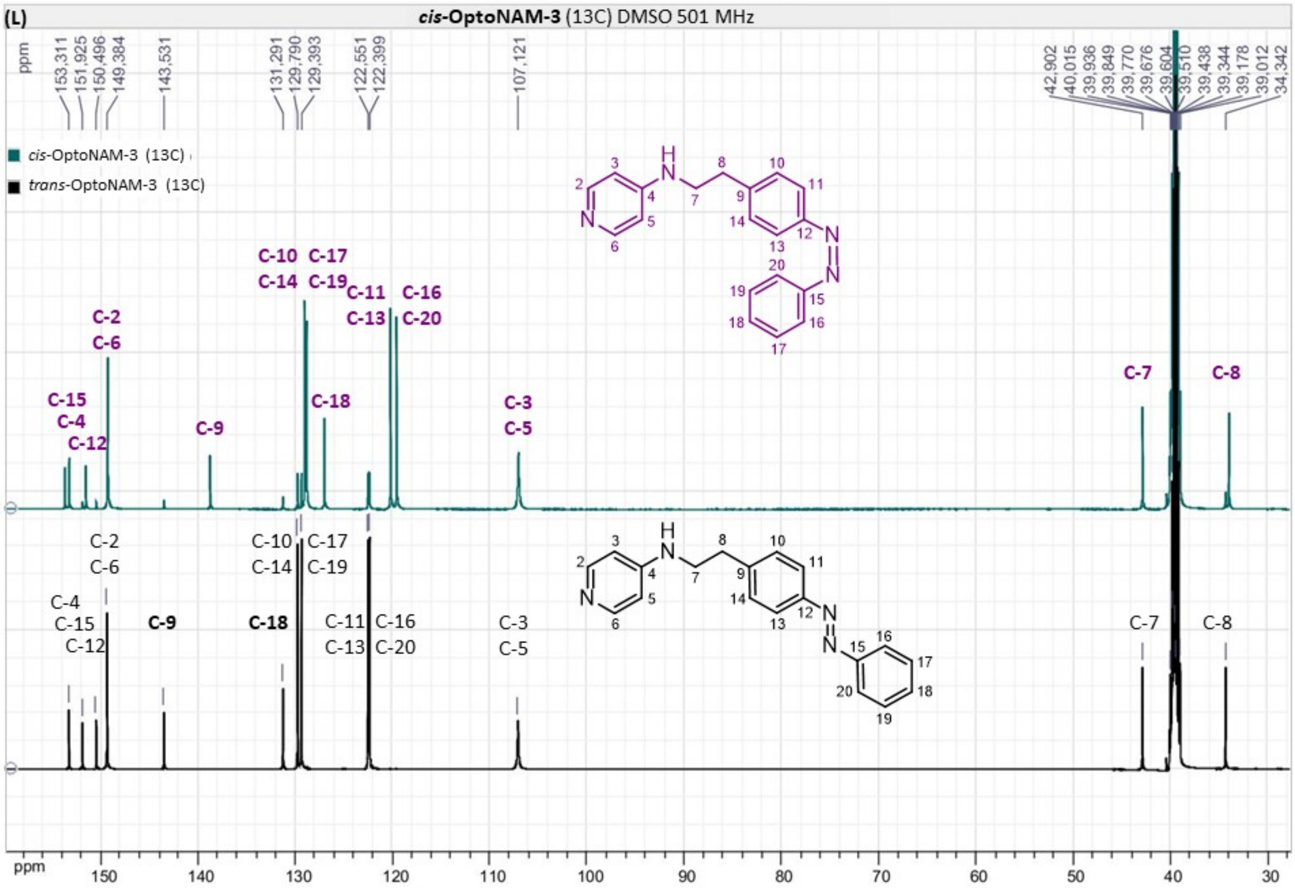

Spectra S3M,N

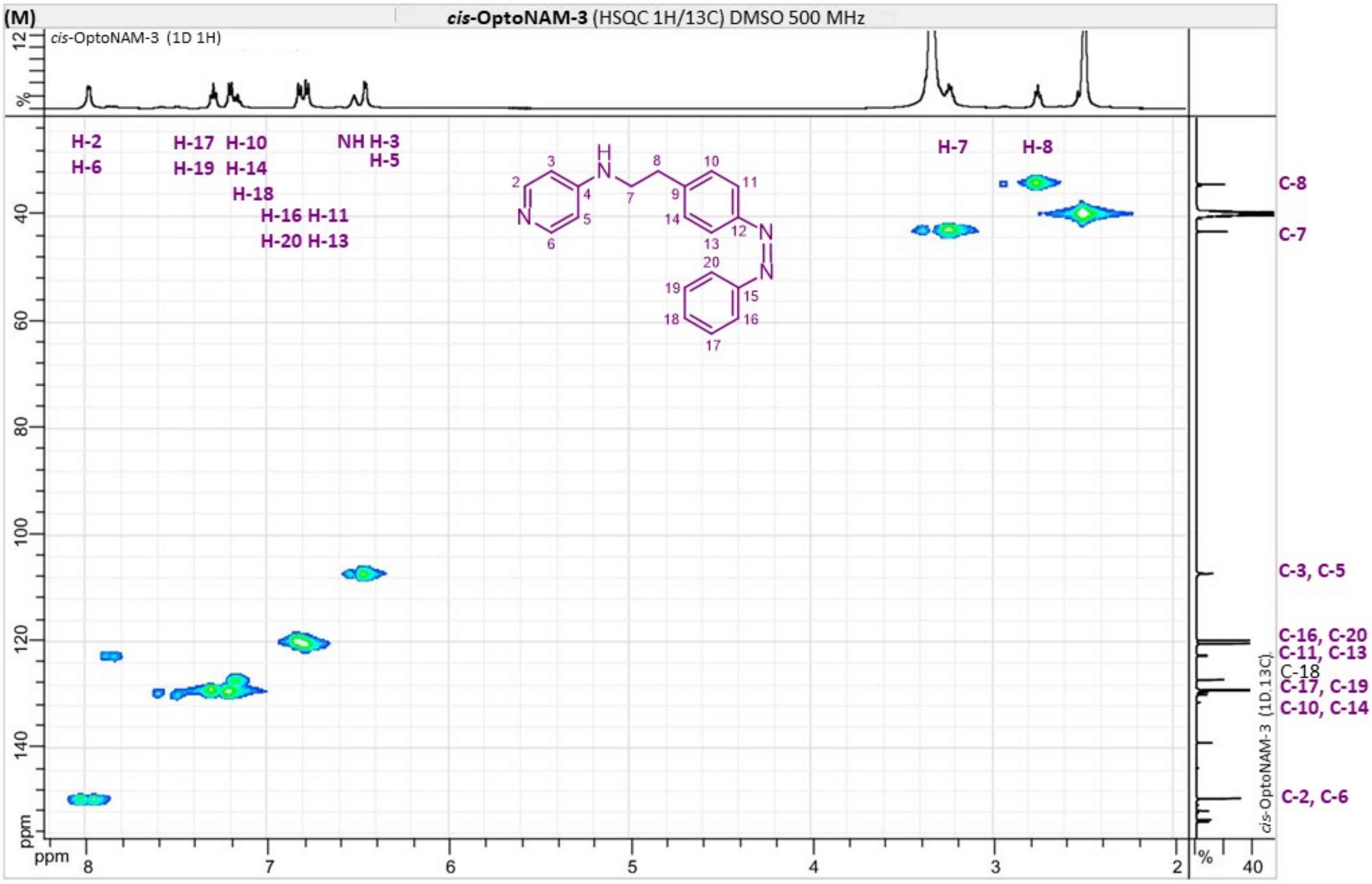

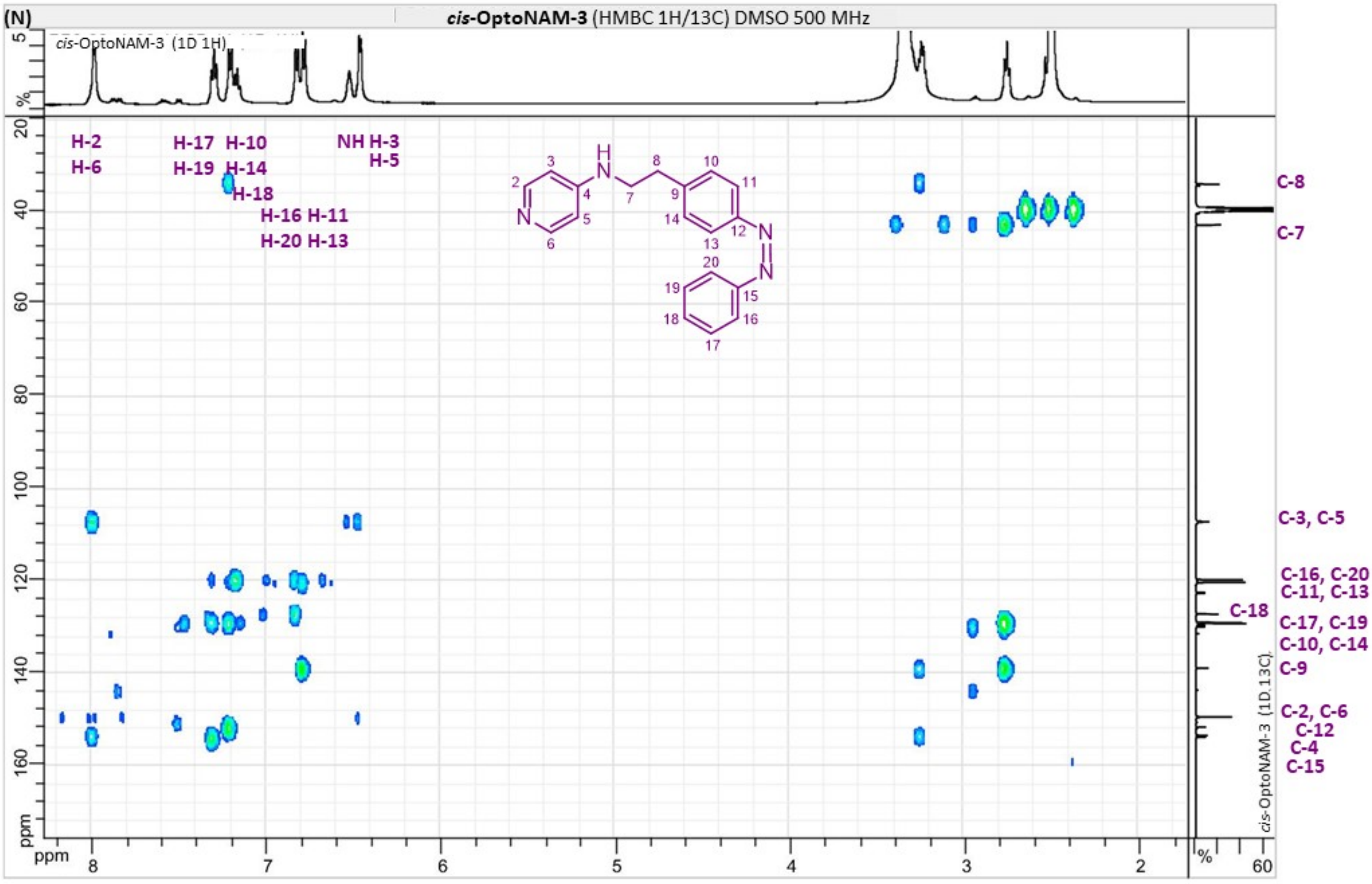

(O) **HPLC-MS of OptoNAM-3**

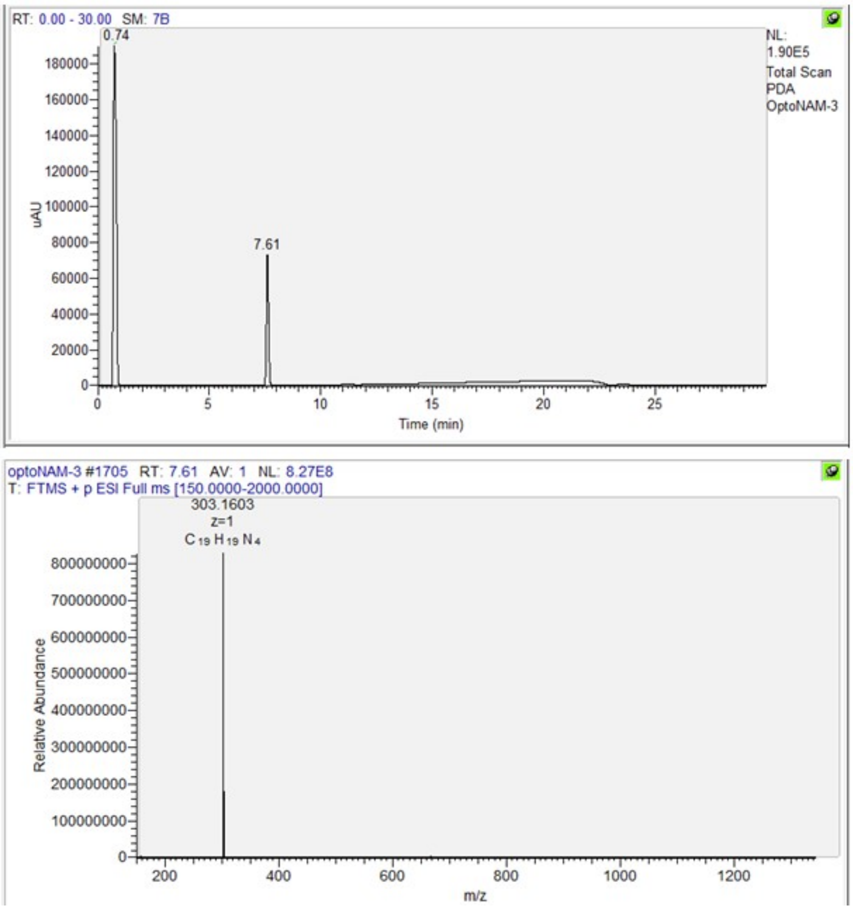

(P) **HPLC-MS of OptoNAM-3: Room Temperature, 2 weeks**

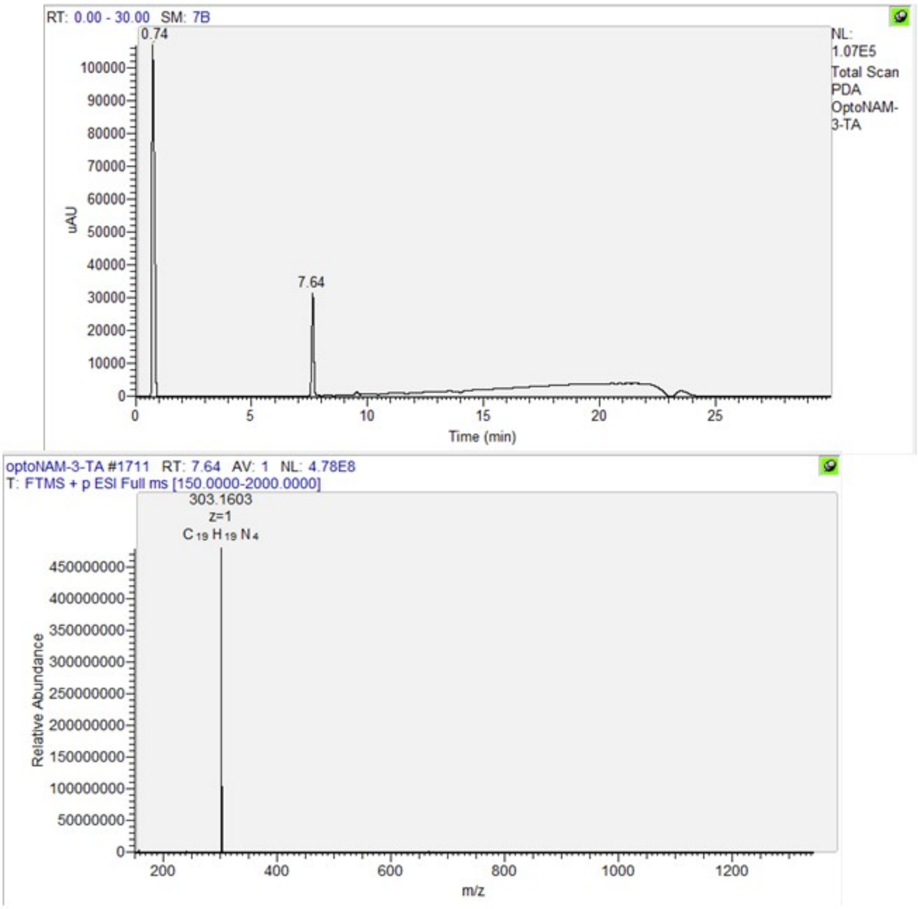

Spectra S4A,B

(A) ***trans*-OptoNAM-4** (1D 1H) DMSO 400 MHz

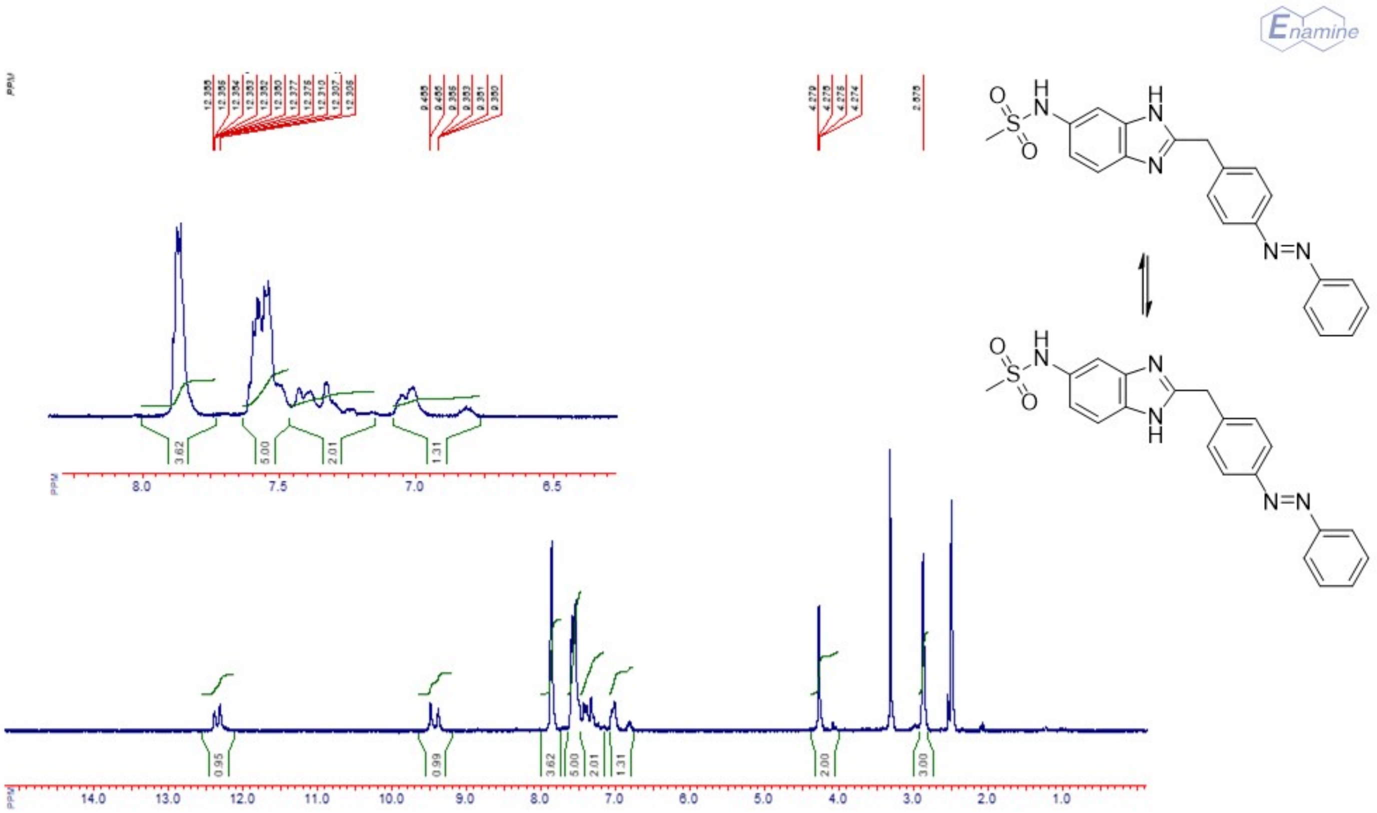

(B) **HPLC-MS of OptoNAM-4**

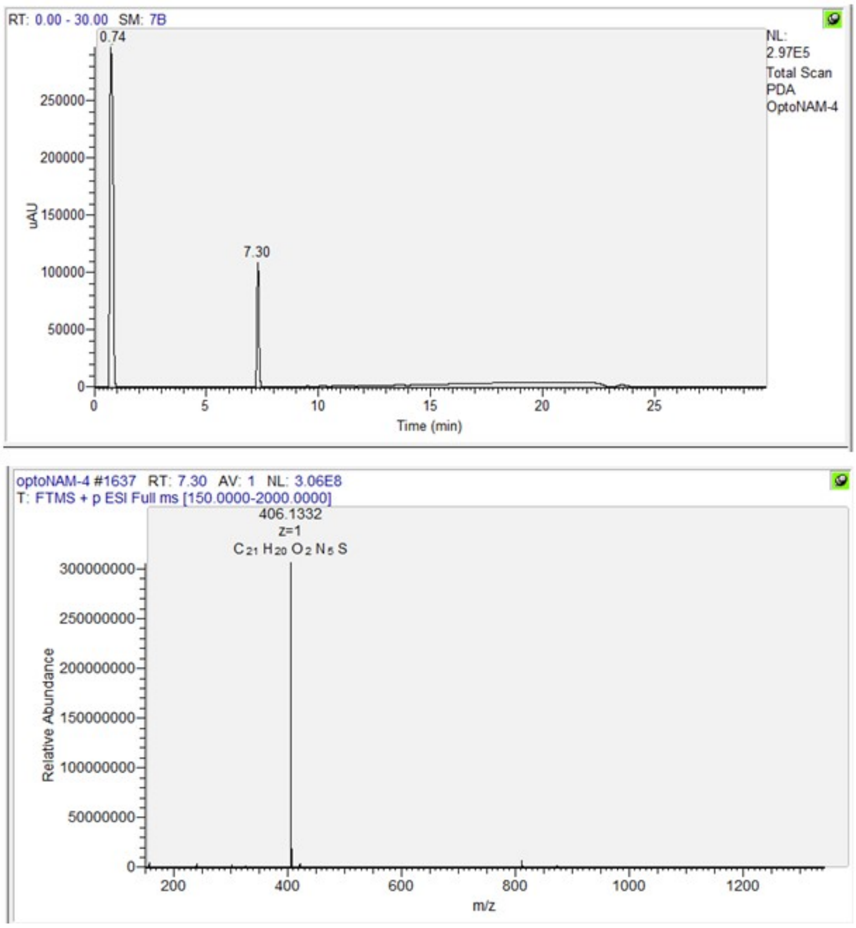

Text S1: Photochemical and biological characterizations of OptoNAM-1, -2 and -4.

To characterize the photochemical properties of OptoNAM-1, -2 and -4, we acquired UV-visible spectra of these compounds diluted in physiological buffer (see Methods) in the dark (*trans* state) and after illumination with various wavelengths. The absorption spectra of OptoNAM-1, -2 and 4 in the dark were characteristic of azobenzenes in their *trans* configuration[1] (black curves in **Figure S1B,F,J**). Application of UV light at a wavelength close to the main absorption peak of the *trans* form (365 nm) gave a completely different spectrum (violet curves in **Figure S1B,F,J**), characteristic of azobenzenes in their *cis* configuration[1].

To determine the appropriate wavelengths to convert the *cis* OptoNAMs back to *trans*, we illuminated the 365 nm PSS OptoNAM-1, -2 and -4 solutions with wavelengths of 440 and 490 nm. 440 nm irradiation provided the strongest *cis*-to-*trans* conversion for OptoNAM-1 and -4 (OptoNAM-1, ~75% *trans* measured at λ_{trans} = 320 nm; OptoNAM-4, 100% *trans* measured at λ_{trans} = 334 nm), while 490 nm was most efficient to convert *cis*-OptoNAM-2 back to *trans* (~52% measured at λ_{trans} = 371 nm) (**Figure S1A,B,E,F,I,J**). The *cis* isomers of all compounds displayed strong thermal stability since the absorption spectrum of the 365 nm PSS kept in the dark did not change over 1 h at room temperature (**Figure S1C,G,K**).

The activities of the dark and 365 nm PSS of OptoNAM-1, -2 and -4 on GluN1/GluN2B NMDARs were assessed by electrophysiology on Xenopus oocytes as described in the main text for OptoNAM-3. OptoNAM-1 and -2 behaved as GluN1/GluN2B NAMs with better apparent affinity in the dark than in the UV condition. However, OptoNAM-1 and 2 in *trans* configuration displayed a drastic loss of potency compared to their parent compounds,[2–6] with a >1000-fold shift in potency (**Figure S1D,H** and **TableS1**). We showed that replacement of the amino-methyl bond of the parent compounds by an azo bond to obtain OptoNAM-1 and -2 induced a loss of protonation of the aminopyridium moiety at physiological pH, which likely disrupts binding of the compounds in the ifenprodil binding site (**FigureS2**). OptoNAM-4, on the other hand, did not display any photodependence of activity (**Figure S1** and **Table S1**).

Text S2: Docking and molecular dynamic simulations

Trans-OptoNAM-3 was docked into the ifenprodil binding pocket using the ifenprodil-bound, GluN1/GluN2B NTD dimer crystal structure 5EWJ[7] (see Methods). After docking, we first performed three simulations of 1 µs with no constraints (Traj01-03 in Figure S7A). They all started from the same structure, but were assigned different initial random velocities during the equilibration procedure (see Methods). We followed the evolution of two dihedral angles during these simulations: one that describes the orientation of the NH from aniline (Figure 7A), and one that describes the orientation of one of the azo group relative to the central azobenzene phenyl (C-C-N=N dihedral angle; orientation of azo; Figure 7A). During these simulations, the C-N=N-C angles from the azo group stayed at 180° i.e. OptoNAM-3 did not convert from *trans* to *cis*. We observed however that both the aniline and C-C-N=N angles could interconvert between 0° and 180° (Figure S7A). Thus, the ligand is flexible in the active site with different possible conformations, and in particular different conformations of the terminal phenyl-azo moiety. We furthermore observed that the protein was also quite flexible. Indeed, the distance between GluN1 and GluN2B NTD lower lobes significantly increased during the simulation (35 Å between the Cα of GluN1-K179 and GluN2B-K185 at the end of the simulations vs 25 Å in the 5EWJ crystal structure). This can easily be explained by the fact that we are simulating a dimer of isolated GluN1 and GluN2B NTDs, whose relative orientation is usually constrained by the rest of the protein in the full-length receptor.[8] We thus decided to perform new simulations where the protein heavy atoms were restrained close to the crystallographic position by a soft harmonic potential of 100 $kJ/mol/nm^2$. One simulation started from the same initial conformation of OptoNAM-3 as before (C-C-N=N angle of 141°, called Rot-1; Figure 7A), whereas in the other we manually rotated the C-C-N=N dihedral angle by 180° (going from 141° to -39°, Rot-2 rotamer; Figure 7A) to reflect the OptoNAM-3 conformational mobility observed in the first round of simulations.

To study the behavior of free *trans*-OptoNAM-3, we performed a 4 µs-long MD simulation of the ligand alone in water. We observed transitions of both the aniline and the C-C-N=N angle all along the trajectory (Figure S7C). Compared to the simulations performed in the protein, we observed much more transitions between each conformation in solution, which means that the free energy barrier to go from one conformation to the other is much smaller in solution than when bound to the protein.

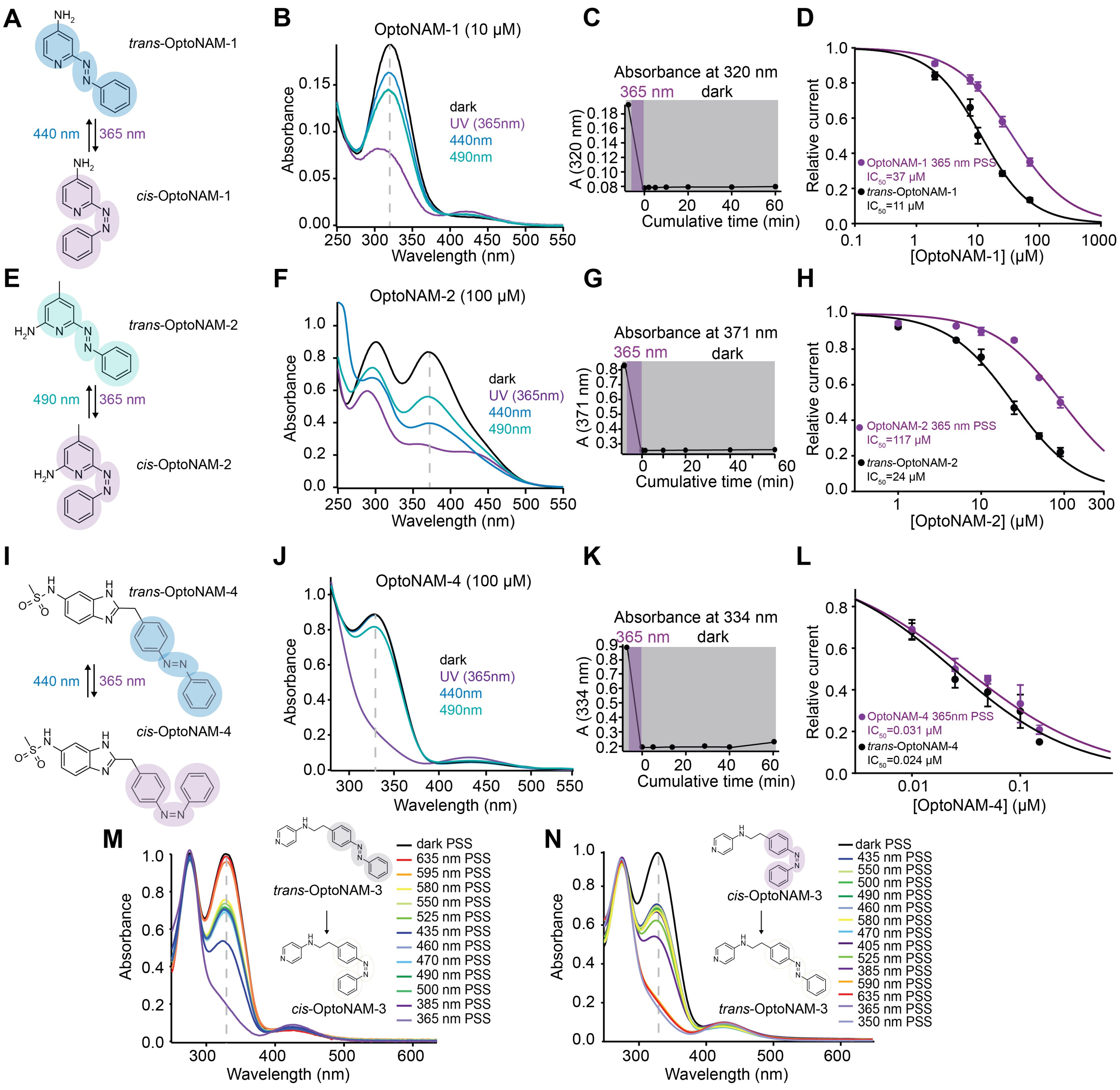

Figure S1. Photochemical properties of OptoNAM-1 to -4 and their photodependent activity at GluN1/GluN2B receptors.

(A-D) OptoNAM-1.

(A) In solution, OptoNAM-1 can be switched from *trans* to *cis* configuration by UV illumination (365 nm) and back to *trans* by 440 nm light. **(B)** UV-visible absorption spectra of OptoNAM-1 in physiological aqueous buffer (Ringer pH 7.3, see Methods) in the dark (black curve), after 365 nm illumination (violet curve) and subsequent illumination of the 365 nm PSS by 440 or 490 nm light. Dashed line represents the wavelength of peak absorption of *trans*-OptoNAM-1 (320 nm). **(C)** OptoNAM-1 365 nm PSS (mostly *cis*) displays strong thermal stability in the dark in Ringer pH 7.3, at room temperature: no change of the absorption spectra was observed up to 60 min after 365 nm illumination. **(D)** Dose-response curves of OptoNAM-1 activity on GluN1/GluN2B receptors in the dark (black curve, IC_{50} = 11 ± 1 µM, n = 4-6) or pre-illuminated with 365 nm (violet curve, IC_{50} = 37 ± 2 µM, n = 5-17).

(E-H) OptoNAM-2.

(E) In solution, OptoNAM-2 can be switched from *trans* to *cis* by UV illumination (365 nm) and back to *trans* by 490 nm light. **(F)** UV-visible absorption spectra of OptoNAM-2 in physiological aqueous buffer (Ringer pH 7.3, see Methods) in the dark (black curve), after 365 nm illumination (violet curve) and subsequent illumination of the 365 nm PSS by 440 or 490 nm light. Dashed line represents the wavelength of peak

absorption of *trans*-OptoNAM-2 (371 nm). **(G)** OptoNAM-2 365 nm PSS (mostly *cis*) displays strong thermal stability in the dark in Ringer pH 7.3, at room temperature: no change of the absorption spectra was observed up to 60 min after 365 nm illumination. **(H)** Dose-response curves of OptoNAM-2 activity on GluN1/GluN2B receptors in the dark (black curve, IC_{50} = 24 ± 2 µM, n = 3-5) or pre-illuminated with 365 nm (violet curve, IC_{50} = 117 ± 10 µM, n = 3-8).

(I-L) OptoNAM-4.

(I) In solution, OptoNAM-4 can be switched from *trans* to *cis* by UV illumination (365 nm) and back to *trans* by 440 nm light. **(J)** UV-visible absorption spectra of OptoNAM-4 in aqueous buffer (Ringer pH 7.3, see Methods) in the dark (black curve), after 365 nm illumination (violet curve) and subsequent illumination of the 365 nm PSS by 440 or 490 nm light. Note that 440 nm allows full return to the dark state. Dashed line represents the wavelength of peak absorption of *trans*-OptoNAM-2 (334 nm). **(K)** OptoNAM-4 365 nm PSS (mostly *cis*) displays strong thermal stability in the dark in Ringer pH 7.3, at room temperature: only little change of the absorption spectra was observed up to 60 min after 365 nm illumination. **(L)** Dose-response curves of OptoNAM-4 activity on GluN1/GluN2B receptors in the dark (black curve, IC_{50} = 24 ± 26 nM, n = 3) or pre-illuminated with 365 nm (violet curve, IC_{50} = 31 ± 29 nM, n = 3).

(M,N) OptoNAM-3 *trans-to-cis* and *cis-to-trans* isomerization for different illumination wavelengths.

(M) OptoNAM-3 UV-visible absorption spectra in physiological aqueous buffer (Ringer pH 7.3) in the dark (black curve) and PSS obtained after illumination with wavelengths ranging from 350 to 635 nm of the dark PSS. These spectra were used to create panel **D** and **E** from Figure 4. **(N)** OptoNAM-3 UV-visible absorption spectra in the dark (black curve), after 365 nm illumination (violet curve), and PSS obtained after illumination with wavelengths ranging from 350 to 635 nm of the 365 nm PSS. These spectra were used to create panels **I** and **J** from **Figure 5**.

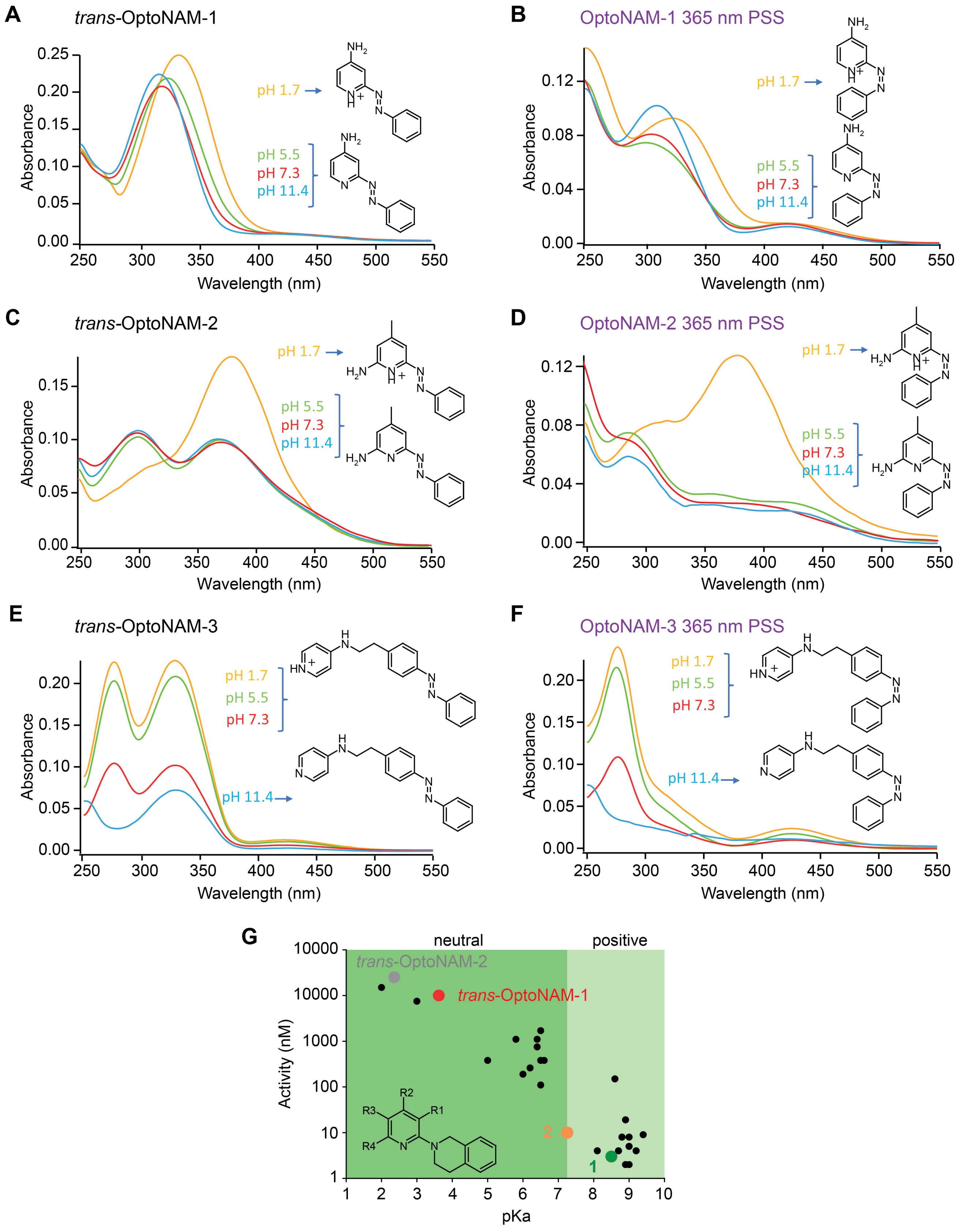

Figure S2. Decreased pKa of OptoNAM-1 and -2 compared to their parent compounds are likely responsible for their decreased activity.

(A-F) pKa estimation of the *trans* (**A**,**C**,**E**) and *cis* (365 nm PSS) (**B**,**D**,**F**) isomers of OptoNAM-1 (**A**,**B**), OptoNAM-2 (**C**,**D**) and OptoNAM-3 (**E**,**F**) by UV-visible spectrum analysis. UV-visible absorption spectra were measured at different pH (1.7 in yellow, 5.5 in green, 7.3 in red and 11.4 in blue). Correspondence between spectra at different pH and OptoNAM protonated and unprotonated chemical structures is indicated. A leftward shift and a decrease of the absorbance peak corresponding to the aminopyridine carrying the charge was observed upon OptoNAM deprotonation. This effect is most obvious for OptoNAM-2 and characteristic of the deprotonated spectra of 2,6-diaminopyridine bases.[9] Based on this analysis, at physiological pH, OptoNAM-1 and -2, either in *cis* or *trans*, are unprotonated, while OptoNAM-3 is protonated.

(G) Relationship between the activity and the measured pKa of compounds from the same chemical series as parent compound **1** (values from ref. 3) (black dots). Parent compound **1** is highlighted as a thick green dot. Parent compound **2** (orange), as well as *trans*-OptoNAM-1 (red) and -2 (grey) were added to the plot according to their published or measured activity (Supplementary Table 1), and predicted pKa (pKa was predicted by Marvin, Chemaxon https://www.chemaxon.com). “Neutral” (dark green) and “Positive” (light green) indicate whether the compounds of a given pKa are neutral (dark green) or positively charged (light green) at physiological pH (pH = 7.3). Note the tight correlation between pKa and activity, suggesting that the decreased pKa of OptoNAM-1 and -2 induced by azologization of the parent compounds, resulting in a loss of protonation at physiological pH, is responsible for the large decrease of activity of these compounds.

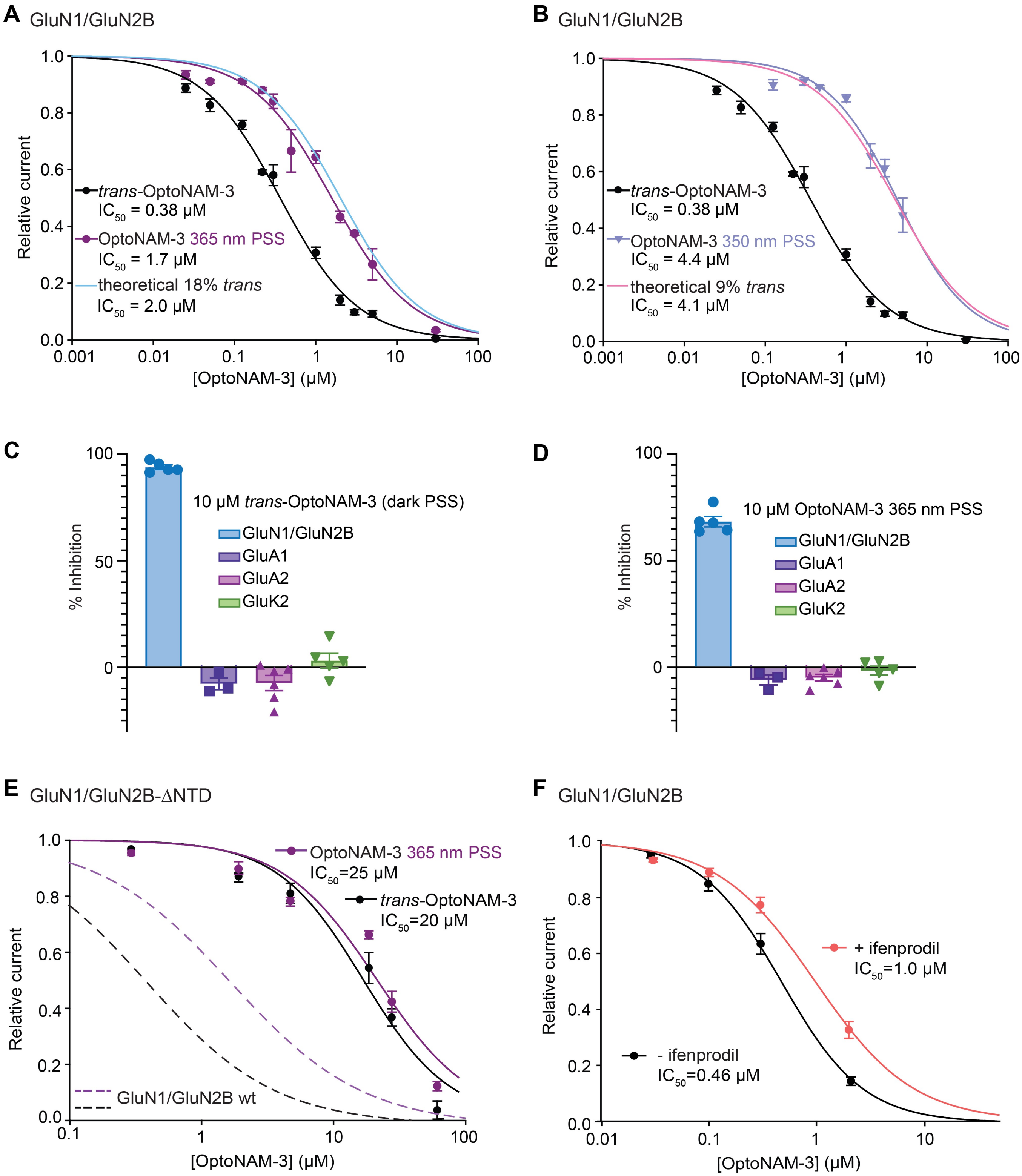

Figure S3. Additional data relative to Figure 3.

(A) Inhibition by OptoNAM-3 365 nm PSS is exclusively mediated by the remaining *trans* isomer still present in solution. (A) Dose-response curves of OptoNAM-3 activity on GluN1/GluN2B in the dark (black curve, IC_{50} = 0.38 ± 0.03 µM, n = 4-21), pre-illuminated by 365 nm (purple curve, IC_{50} = 1.7 ± 0.2 µM, n = 4-17) and theoretical dose-response curve (blue) of a mixture of 18% *trans*- and 82% *cis*-OptoNAM-3 (corresponding to the 365 nm PSS determined by HPLC) assuming that only the *trans* isomer is active. The theoretical curve was calculated from the *trans* dose-response curve in the dark. The 18% *trans* theoretical dose-response curve superimposes well to the one of OptoNAM-3 365 nm PSS. **(B)** Dose-response curves of OptoNAM-3 activity on GluN1/GluN2B in the dark (black curve, IC_{50} = 0.38 ± 0.03 µM, n = 4-21), pre-illuminated by 350 nm (lavender curve, IC_{50} = 4.4 ± 0.6 µM n = 3-13) and theoretical dose-response curve (pink) of a mixture of 9% *trans*- and 91% *cis*-OptoNAM-3 (corresponding to the 350 nm PSS determined by HPLC) assuming only the *trans* isomer is active. The 9% *trans* theoretical dose response curve is also well superposed to the one of OptoNAM-3 350 nm PSS. This shows that the activity of the 365 nm PSS and 350 nm PSS of OptoNAM-3 can entirely be explained by the amount of remaining *trans* isomer in the PSS.

(C,D) Among iGluRs, OptoNAM-3 is selective for NMDARs. Inhibition by 10 µM *trans*-OptoNAM-3 (dark PSS, **C**) and OptoNAM-3 365 nm PSS **(D)** of agonist-induced currents (glutamate and glycine, 100 µM each) carried by GluN1/GluN2B NMDARs, GluA1 and GluA2 AMPA receptors, and GluK2 kainate receptors. Oocytes expressing GluK2 receptors were treated with concanavalin A (2 mM) before recording to decrease receptor desensitization. **(C)** *Trans*-OptoNAM-3 inhibitions: 94 ± 1%, n = 5 for GluN1/GluN2B; -7.4 ± 2.7%, n = 3 for GluA1; -7.4 ± 3.5%, n = 6 for GluA2, 3.1 ± 3.4%, n = 5 for GluK2. **(D)** *Cis*-OptoNAM-3 inhibitions: 68 ± 6%, n = 5 for GluN1/GluN2B, -6.0 ± 2.3%, n = 3 for GluA1, -4.9 ± 1.5%, n = 6 for GluA2, -1.5 ± 2.1%, n = 5 for GluK2.

(E,F) OptoNAM-3 binds at the ifenprodil binding site. (E) Dose-response curves of OptoNAM-3 on GluN1/GluN2B-ΔNTD receptors in the dark (black curve, IC_{50} = 20 ± 4 µM, n = 3-5) or pre-illuminated with 365 nm (violet curve, IC_{50} = 25 ± 6 µM, n = 3-5). OptoNAM-3 dose-response curves on wild type GluN1/GluN2B receptors are shown as dashed curves (black and violet for the dark and 365 nm PSS conditions, respectively). The remaining, low affinity inhibition of OptoNAM-3 on GluN1/GluN2B-ΔNTD receptors is likely due to a non-selective pore block of NMDARs at negative holding potentials, as previously shown for ifenprodil.[10,11] **(F)** Dose-response curves of *trans*-OptoNAM-3 in the dark in absence (black curve, IC_{50} = 0.46 ± 0.06 µM, n = 4) and in presence of 0.2 µM ifenprodil (a concentration close to ifenprodil IC_{50}, orange curve). Ifenprodil increases OptoNAM-3 IC_{50} by ~2-fold (IC_{50} = 1.0 ± 0.1 µM, n = 5 in presence of 0.2 µM ifenprodil), which is consistent with a competitive interaction between these two compounds.

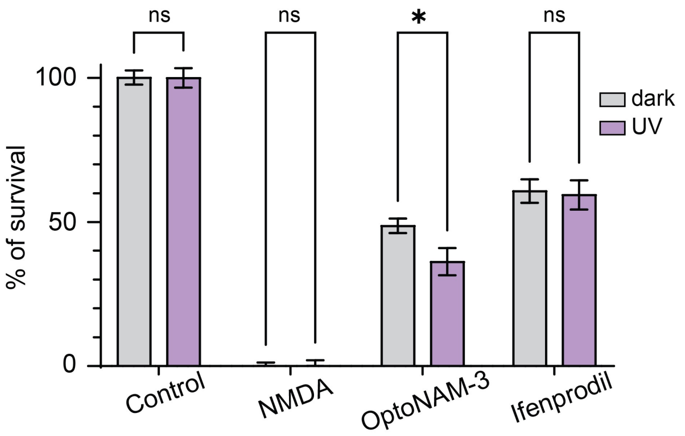

Figure S4. OptoNAM-3 decreases NMDA-induced neuronal death in a photodependent manner.
Percentage of neuronal survival in cultured cortical neurons exposed either to control (0.01% DMSO and 10 µM Glycine), NMDA (100 µM NMDA + 10 µM Glycine), NMDA + ifenprodil or NMDA + OptoNAM-3 (100 µM NMDA + 10 µM Glycine + 5 µM of inhibitor), in the dark (grey bar) or after 2 min UV (365 nm) illumination (violet bar). In the presence of *trans*-OptoNAM-3 (dark) or ifenprodil, cell survival increased to 50% and 60%, respectively. When 365 nm illumination followed the addition of OptoNAM-3, cell survival was decreased to 35% but the extent of survival induced by ifenprodil was not affected, precluding any deleterious effect of the UV light treatment on cell survival. Multiple comparisons were performed by two-way ANOVA with Bonferroni's correction; n.s., $p > 0.05$; *, $p < 0.05$; n = 5-8 batches of cultures per condition, in each culture 4-6 wells/condition. Data presented here (mean and SEM) were normalized to the control and NMDA conditions (see Methods for the calculation protocol).

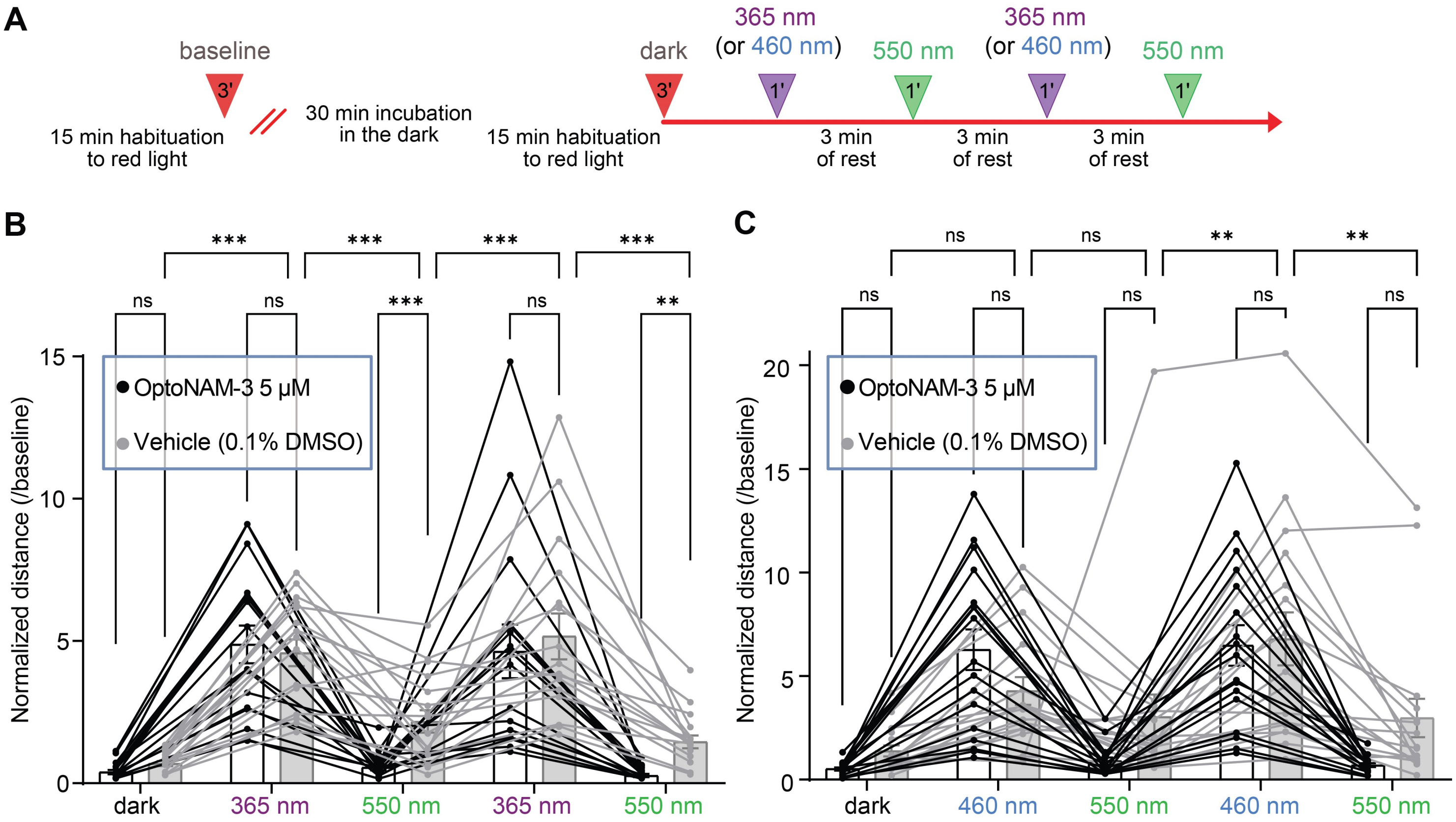

Figure S5. OptoNAM-3 photomodulates Xenopus tadpole locomotion *in vivo*: protocol and tadpole locomotion normalized to baseline locomotion. Additional data relative to Figure 6.

(**A**) Experimental design of the behavioral tests performed on tadpoles incubated in control (0.1% DMSO) or OptoNAM-3 at 5 µM and exposed to UV/green (or blue/green) light cycles. **(B,C)** Normalized distance (compared to baseline) traveled by tadpoles (1 point represents the mean distance traveled by the 3 tadpoles of one well) incubated in control (white bars and black points) or in 5 µM OptoNAM-3 (grey bars and points), in the dark and during UV/green light cycles (or blue-green light cycles for panel C). Note that UV light influences tadpole locomotion on its own. n = 16 wells for (B) and n = 17 wells for (C), which corresponds to a total of 48 and 51 tadpoles, respectively. The means of 3 tadpoles per well were used to conduct a paired statistical test. n.s., $p > 0.05$; ***, $p < 0.001$; **, $p < 0.01$; Friedman test followed by Dunn's multiple comparison test.

A

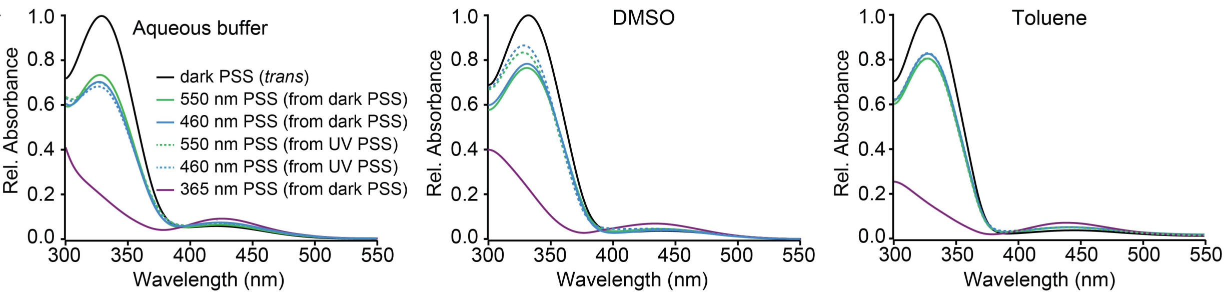

B

	Dark PSS[a]	550 nm PSS from dark PSS[a]	460 nm PSS from dark PSS[a]	550 nm PSS from UV PSS[a]	460 nm PSS from UV PSS[a]	365 nm PSS[a]
Aqueous buffer	329 (1), *~421 (0.057)*	328 (0.73), *~424 (0.062)*	327 (0.70), *~424 (0.073)*	327 (0.70), *~422 (0.069)*	326 (0.68), *~424 (0.069)*	<300, *426 (0.09)*
DMSO	332 (1), *~441 (0.041)*	331 (0.75), *~438 (0.044)*	330 (0.78), *~441 (0.039)*	328 (0.83), *~438 (0.044)*	329 (0.87), *~434 (0.049)*	<300, *4 34 (0.068)*
Toluene	329 (1), *~445 (0.026)*	328 (0.78), *~443 (0.037)*	328 (0.80), *~441 (0.038)*	327 (0.78), *~441 (0.037)*	328 (0.80), *~440 (0.038)*	<300, *400 (0.057)*

[a] π → π* transition, *n → π* transition* (in nm). Absorbance of the peaks relative to the absorbance of the π → π* transition peak of the dark PSS is indicated in parenthesis

Figure S6. OptoNAM-3 photochemical properties in different solvents.
(A) UV-visible absorption spectra of OptoNAM-3 (25 µM) in physiological aqueous solution (Ringer at pH 7.3), DMSO and toluene. Black curves represent the dark PSS, the violet ones OptoNAM-3 365 nm PSS, the blue and green curves represent OptoNAM-3 460 nm and 550 nm PSS obtained after illumination of the dark PSS, and the dotted blue and green curves represent OptoNAM-3 460 nm and 550 nm PSS obtained after illumination of the 365 nm PSS. **(B)** Maximum absorption wavelengths of the peaks corresponding to the π → π* transition (in regular police) and n → π* transition (in italic) for the different OptoNAM-3 PSS in the different solvents. For each solvent, the absorbance of each peak relative to the absorbance of the π → π* transition peak of the dark PSS is indicated in parenthesis.

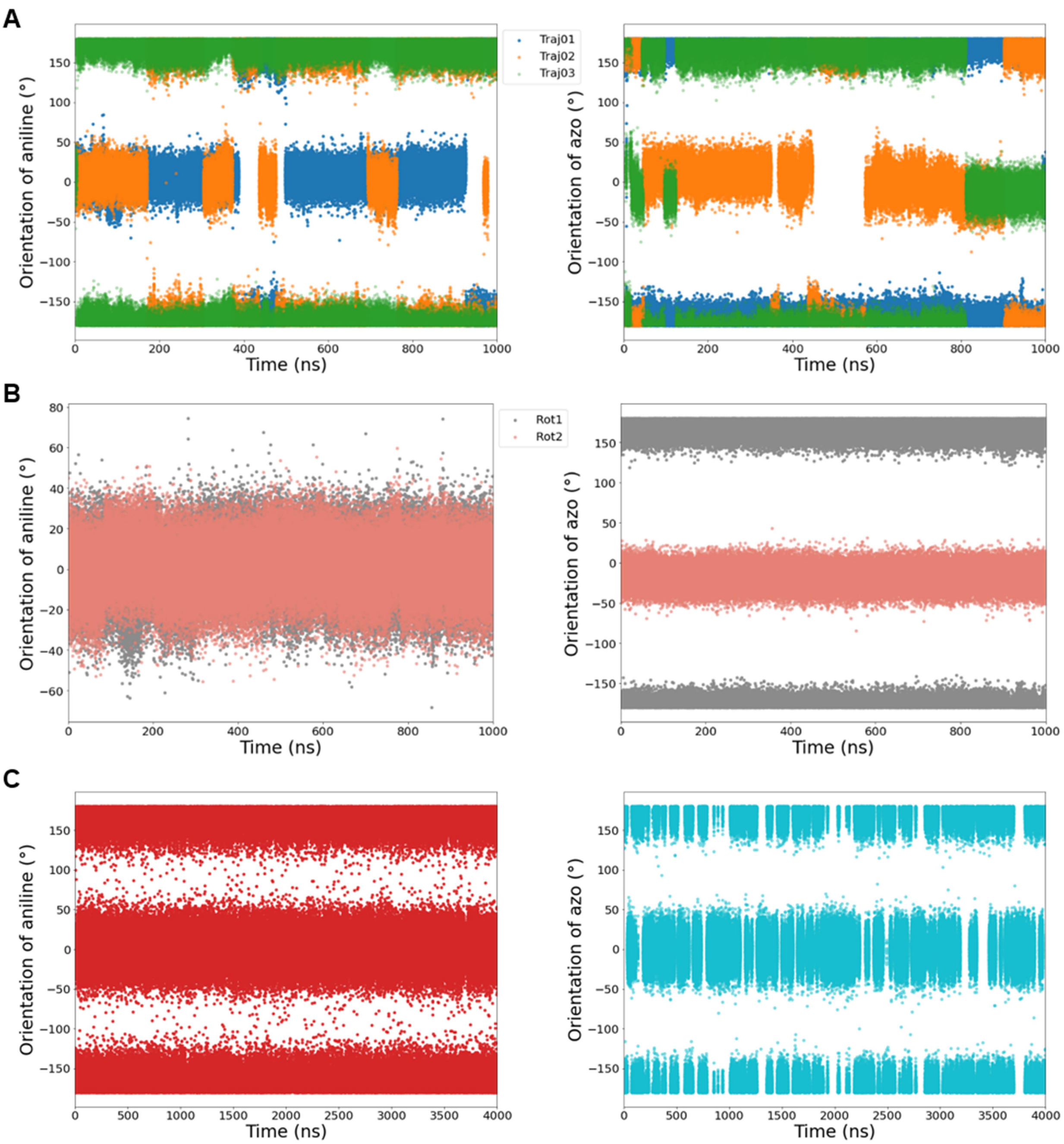

Figure S7. Evolution of *trans*-OptoNAM-3 conformation in its binding site and in water.

(A) Evolution of the two angles that describe bound *trans*-OptoNAM-3 conformations during 3 trajectories of a MD simulation without constraints. The three colors represent the three trajectories. Left: orientation of the aniline. Right: orientation of the phenyl-azo moiety (C-C-N=N angle) (**Figure 7A**). For the orientation of aniline, in trajectories 01 and 02 we observed exchanges between 0 and 180°, whereas in trajectory 03 the angle stayed at 180°. On average, this angle is 60.8% at 180°, which means that this conformation is more stable by roughly 0.3 kcal/mol than the one at 0°. For the orientation of the azo moiety, in trajectory 01 the angle stayed at 180° and switched to 0° after 993 ns; for trajectory 02, we observed six conversions between the two basins; for trajectory 03, we observed some exchanges at the beginning, a stability from 125 to 810 ns, and then a final exchange. On average, this angle is 67.9% at 180°, which means that this conformation is more stable by roughly 0.4 kcal/mol than the one at 0°. At the end of this simulation without constraints, we observed that the distance between the GluN1 and GluN2B lower lobes had increased, far from the distance measured in the inhibited, full-length receptor (see Text S2). We therefore decided to perform new simulations where the protein heavy atoms were restrained close to their crystallographic positions (see Text S2 and Methods) **(B)** Evolution of the same angles but for the Rot-1 (grey) and Rot-2 (salmon) rotamers of OptoNAM-3 during the simulations under constraint. Left: orientation of the aniline. Right: orientation of the phenyl-azo moiety (C-C-N=N angle). **(C)** Evolution of the two angles for free *trans*-OptoNAM-3 in water. For the orientation of aniline, the two basins at 0° and 180° are populated respectively 48.2% and 51.8% of the time, whereas for the orientation of the phenyl-azo they are populated at

respectively 53.5% and 46.5% of the time. This corresponds to differences of free energies below 0.1 kcal/mol. We observed very low differences of free energies between the conformations of *trans*-OptoNAM-3 both in the protein and in water solution; however, in solution, we observed much more transitions between each conformation, which means that the free energy barrier to go from one conformation to the other is much smaller in solution than when bound to the protein.

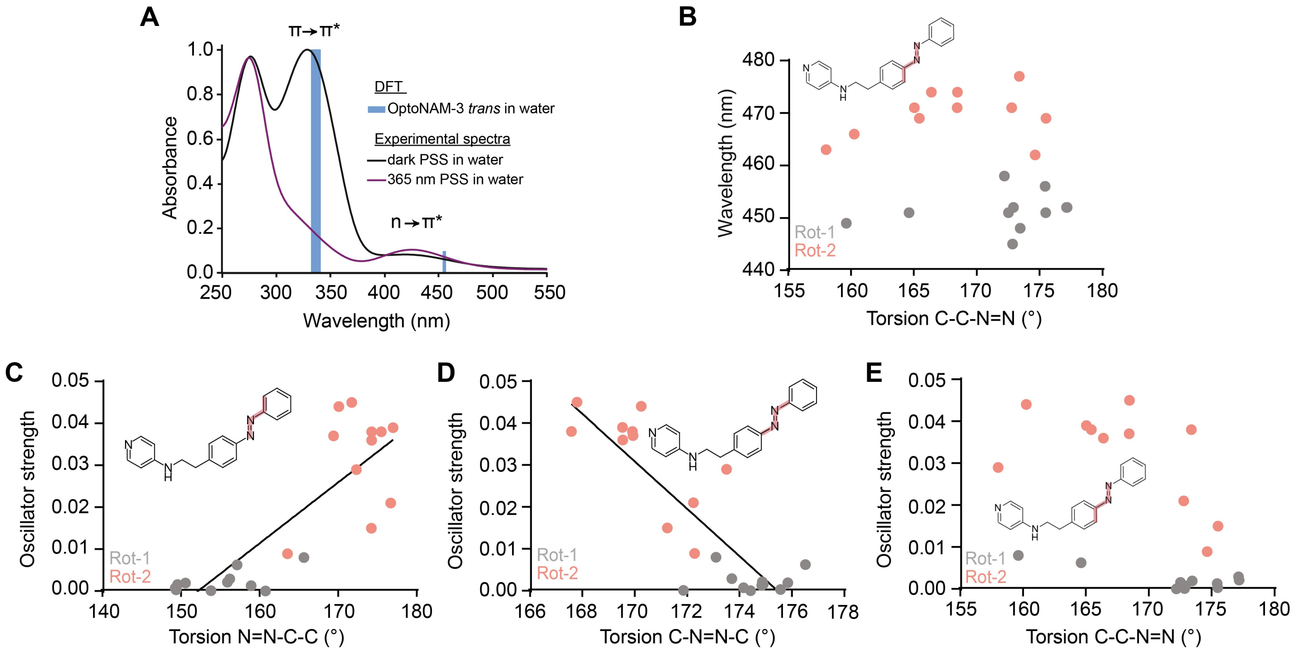

Figure S8. Additional data relative to Figure 7.

(A) Superposition of the experimental π → π* and n → π* bands of the UV-Vis spectra of free OptoNAM-3 dark PSS (*trans* state, dark line) and 365 nm PSS (violet line) in aqueous buffer (Ringer pH 7.3), to the theoretical π → π* and n → π* transitions of OptoNAM-3 in implicit water computed by DFT calculations (blue bars representing the range of computed wavelengths for *trans*-OptoNAM-3 across the different snapshots of the dynamic, see Table S2). **(B)** Relationship between the C-C-N=N torsion angle (as highlighted in pale red in the inset chemical structure) of bound *trans*-OptoNAM-3 in the 11 snapshots selected for DFT calculations and their computed n→π* absorption wavelengths, for Rot-1 (in grey) and Rot-2 (in salmon). **(C-E)** Relationships between the N=N-C-C **(C)**, C-N=N-C **(D)**, and the C-C-N=N **(E)** torsion angles (as highlighted in pale red in the inset chemical structures) of bound *trans*-OptoNAM-3 in the 11 snapshots selected for DFT calculations and their predicted oscillator strengths, for Rot-1 (in grey), and Rot-2 (in salmon). Linear regressions: R^2 = 0.746 **(C)** and 0.706 **(D)**.

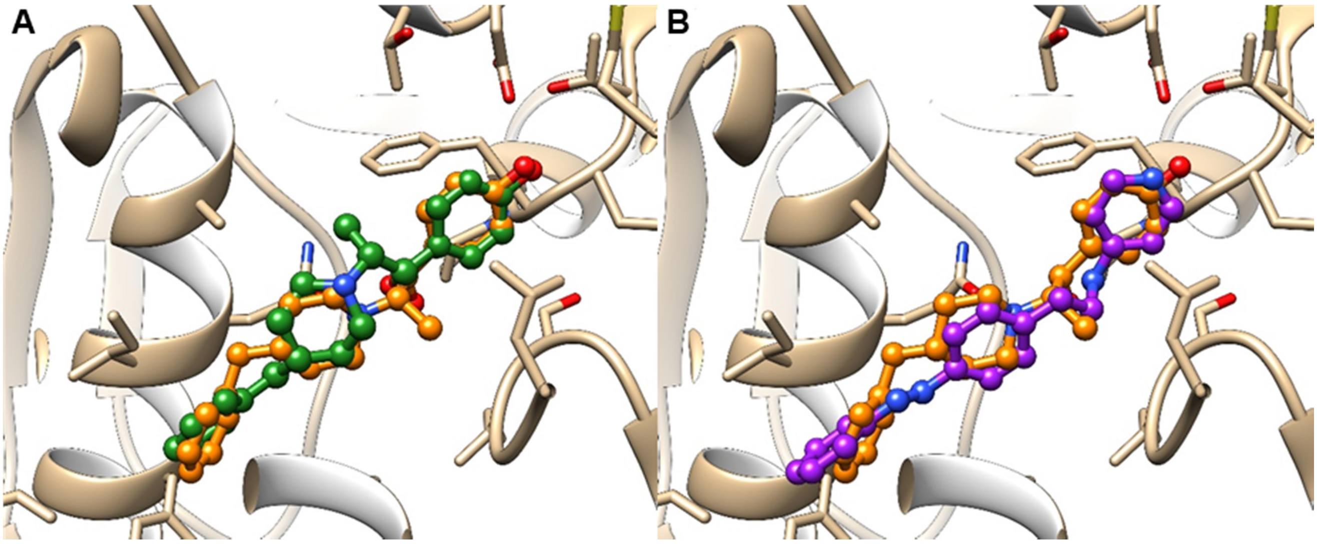

Figure S9. (A) Overlap between crystallographic (in orange) and docked (in green) poses of ifenprodil. **(B)** Overlap between the crystallographic pose of ifenprodil (in orange) and the docked pose of *trans*-OptoNAM-3 (in purple).

Table S1. Summary of the IC_{50}s of OptoNAMs in the dark and UV compared to the activity of their parent compounds.[2–6]

OptoNAM-	Parent compound IC_{50} or Ki (μM)	IC_{50} (μM) In the dark (number of cells)	IC_{50} (μM) of the 365 nm [or 350 nm] PSS (number of cells)	UV/dark IC_{50} ratio
**1**	0.003 (a)	11 ± 1 (n = 4-6)	37 ± 2 (n = 5-17)	3.4
**2**	0.010 (b)	24 ± 2 (n = 3-5)	117 ± 10 (n = 3-8)	4.9
**3**	0.093 (c)	0.38 ± 0.03 (n = 5-21)	1.7 ± 0.2 (n = 5-17) [4.4 ± 0.6 (n = 4-13)]	4.5 [11.5]
**4**	0.00068 (b)	0.024 ± 0.026 (n = 3)	0.031 ± 0.029 (n = 3)	1.3

(a) Ki from ref. 3
(b) IC_{50} from ref. 2,6
(c) Ki from ref. 5
(d) Ki from ref. 4

Table S2. Computed vertical energies and oscillator strengths of 11 snapshots of free *trans*-OptoNAM-3 in implicit water for the 2 first visible transitions using the B2PLYP functional.

Snapshot (MD time)	Computed vertical energy in eV (nm) and oscillator strength		Snapshot (MD time)	Computed vertical energy in eV (nm) and oscillator strength	
	Transition 1 (n → π*)	Transition 2 (π → π*)		Transition 1 (n → π*)	Transition 2 (π → π*)
0 (3 μs)	2.72 (456) f = 0.000029	3.64 (341) f = 0.884	6 (3.6 μs)	2.72 (455) f = 0.0038	3.65 (339) f = 0.917
1 (3.1 μs)	2.72 (456) f = 0.00023	3.69 (336) f = 1.093	7 (3.7 μs)	2.72 (455) f = 0.0021	3.69 (335) f = 1.09
2 (3.2 μs)	2.73 (454) f = 0.0067	3.69 (336) f = 1.05	8 (3.8 μs)	2.73 (455) f = 0.0093	3.72 (339) f = 1.07
3 (3.3 μs)	2.73 (455) f = 0.0042	3.70 (335) f = 1.08	9 (3.9 μs)	2.72 (456) f = 0.00062	3.69 (336) f = 1.09
4 (3.4 μs)	2.74 (453) f = 0.013	3.73 (332) f = 1.06	10 (4 μs)	2.73 (455) f = 0.0021	3.67 (338) f = 1.12
5 (3.5 μs)	2.72 (456) f = 0.00065	3.65 (340) f = 0.92			

Table S3: Computed vertical energies and oscillator strengths of 11 snapshots of bound *trans*-OptoNAM-3 inside the protein for the 2 rotamers and for the 2 first visible transitions using the B2PLYP functional.

Snapshot (MD time)	Computed vertical energy in eV (nm) and oscillator strength		Computed vertical energy in eV (nm) and oscillator strength	
	Rot-1 (n → π*)	Rot-1 (π → π*)	Rot-2 (n → π*)	Rot-2 (π → π*)
0 (900 ns)	2.74 (452) f = 0.00073	3.95 (314) f = 0.94	2.64 (469) f = 0.038	3.95 (314) f = 0.94
1 (910 ns)	2.77 (448) f=0.0019	3.95 (314) f = 0.99	2.63 (471) f = 0.021	3.94 (315) f = 1.00
2 (920 ns)	2.74 (452) f=0.0021	3.88 (320) f = 0.96	2.61 (474) f=0.045	3.99 (311) f = 0.96
3 (930 ns)	2.74 (452) f=0.0029	3.89 (319) f = 0.97	2.61 (474) f=0.036	3.99 (311) f = 0.95
4 (940 ns)	2.79 (445) f=0.00009	3.92 (318) f = 0.98	2.64 (469) f = 0.015	3.89 (319) f = 0.97
5 (950 ns)	2.75 (451) f=0.00031	3.91 (317) f = 0.96	2.60 (477) f = 0.038	3.94 (315) f = 0.96
6 (960 ns)	2.70 (458) f=0.00002	3.92 (318) f = 0.99	2,68 (462) f = 0.0089	3.92 (318) f = 0.95
7 (970 ns)	2.72 (456) f=0.0013	3.91 (317) f = 0.97	2.63 (471) f = 0.037	4.01 (309) f = 0.97
8 (980 ns)	2.76 (449) f=0.0008	3.88 (320) f = 0.97	2.66 (466) f=0.044	4.00 (310) f = 0.93
9 (990 ns)	2.75 (451) f=0.0016	3.99 (311) f = 0.96	2.68 (463) f=0.029	4.03 (306) f = 0.96
10 (1000 ns)	2.75 (451) f=0.00629	3.97 (312) f = 0.96	2.63 (471) f=0.039	3.93 (316) f = 0.95

Table S4: Computed vertical energies and oscillator strengths of snapshot 0 of bound *trans*-OptoNAM-3 for the 2 rotamers and for the 1st transition ($n \rightarrow \pi^*$), inside the protein (**1**, first line); without the protein and compound geometry frozen (**2**, second line), and without the protein after being optimized in vacuum (**3**, third line).

Snapshot (MD time)	Computed vertical energy in eV (nm) and oscillator strength		
	Rot1 ($n \rightarrow \pi^*$)	Rot2 ($n \rightarrow \pi^*$)	
0 (900 ns)	2.74 (452)<br>f = 0.00073	2.64 (469)<br>f = 0.038	**1**
0 (900ns) without protein, in vacuum, compound frozen (electrostatic contribution)	2.72 (456)<br>f = 0.0012	2.63 (471)<br>f = 0.037	**2**
0 (900ns) without protein + optimization in vacuum (geometric contribution)	2.65 (467)<br>f = 0.0011	2.65 (467)<br>f = 0.000046	**3**

Supplementary Movie 1 (separate file). Effect of OptoNAM-3 on tadpole locomotion in the dark and after UV and Green light cycles. Video recording of stage 49 *Xenopus laevis* tadpoles at the center of a 12-well plate in physiological solution in the dark (baseline), then after 45 min incubation in either control (0.1% DMSO; right well) or in 5 µM of OptoNAM-3 (left well), in the dark and after 2 cycles of 365 and 550 nm light illumination. Tadpoles exposed to OptoNAM-3 present a reduction in their locomotion pattern. UV light switching off OptoNAM-3 activity allows tadpoles to have similar behavior as tadpoles exposed to control solution and their locomotion is reduced again by applying green light.

Supplementary Movie 2 (separate file). Effect of OptoNAM-3 on tadpole locomotion in the dark and after Blue and Green light cycles. Video recording of stage 49 *Xenopus laevis* tadpoles at the center of a 12-well plate in physiological solution in the dark (baseline), then after 45 min incubation in either control (0.1% DMSO; left well) or in 5 µM of OptoNAM-3 (right well), in the dark and after 2 cycles of 460 nm and 550 nm light illumination. Tadpoles exposed to OptoNAM-3 present a reduction in their locomotion pattern. Blue light switching off OptoNAM-3 activity allows tadpoles to have similar behavior as tadpoles exposed to control solution and their locomotion is reduced again by applying green light.

Supplementary Dataset 1 (separate file). Snapshots and input files for QM/MM simulations

Supplementary Dataset 2 (separate file). Input files for MD simulations

Supplementary Dataset 3 (separate file). Source data

References

(1) Merino, E.; Ribagorda, M. Control over Molecular Motion Using the Cis-Trans Photoisomerization of the Azo Group. *Beilstein J Org Chem* **2012**, *8*, 1071–1090.

(2) McCauley, J. A. NR2B Subtype-Selective NMDA Receptor Antagonists: 2001 – 2004. *Expert Opinion on Therapeutic Patents* **2005**, *15* (4), 389–407. https://doi.org/10.1517/13543776.15.4.389.

(3) Büttelmann, B.; Alanine, A.; Bourson, A.; Gill, R.; Heitz, M.-P.; Mutel, V.; Pinard, E.; Trube, G.; Wyler, R. 2-(3,4-Dihydro-1H-Isoquinolin-2yl)-Pyridines as a Novel Class of NR1/2B Subtype Selective NMDA Receptor Antagonists. *Bioorganic & Medicinal Chemistry Letters* **2003**, *13* (5), 829–832.

(4) McCauley, J. A.; Theberge, C. R.; Romano, J. J.; Billings, S. B.; Anderson, K. D.; Claremon, D. A.; Freidinger, R. M.; Bednar, R. A.; Mosser, S. D.; Gaul, S. L.; Connolly, T. M.; Condra, C. L.; Xia, M.; Cunningham, M. E.; Bednar, B.; Stump, G. L.; Lynch, J. J.; Macaulay, A.; Wafford, K. A.; Koblan, K. S.; Liverton, N. J. NR2B-SelectiveN-Methyl-D-Aspartate Antagonists: Synthesis and Evaluation Of5-Substituted Benzimidazoles. *J. Med. Chem.* **2004**, *47* (8), 2089–2096.

(5) Liverton, N. J.; Bednar, R. A.; Bednar, B.; Butcher, J. W.; Claiborne, C. F.; Claremon, D. A.; Cunningham, M.; DiLella, A. G.; Gaul, S. L.; Libby, B. E.; Lyle, E. A.; Lynch, J. J.; McCauley, J. A.; Mosser, S. D.; Nguyen, K. T.; Stump, G. L.; Sun, H.; Wang, H.; Yergey, J.; Koblan, K. S. Identification and Characterization of 4-Methylbenzyl 4-[(Pyrimidin-2-Ylamino)Methyl]Piperidine-1-Carboxylate, an Orally Bioavailable, Brain Penetrant NR2B Selective N-Methyl-D-Aspartate Receptor Antagonist. *J Med Chem* **2007**, *50* (4), 807–819.

(6) Alanine, A.; Buettelmann, B.; Neidhart, M.-P. H.; Pinard, E.; Wyler, R. Pyridine Derivatives as Nmda-Receptor Subtype Blockers. WO2003037333A1, May 8, 2003.

(7) Stroebel, D.; Buhl, D. L.; Knafels, J. D.; Chanda, P. K.; Green, M.; Sciabola, S.; Mony, L.; Paoletti, P.; Pandit, J. A Novel Binding Mode Reveals Two Distinct Classes of NMDA Receptor GluN2B-Selective Antagonists. *Mol Pharmacol* **2016**, *89* (5), 541–551.

(8) Tian, M.; Stroebel, D.; Piot, L.; David, M.; Ye, S.; Paoletti, P. GluN2A and GluN2B NMDA Receptors Use Distinct Allosteric Routes. *Nat Commun* **2021**, *12* (1), 4709.

(9) Kaljurand, I.; Rodima, T.; Leito, I.; Koppel, I. A.; Schwesinger, R. Self-Consistent Spectrophotometric Basicity Scale in Acetonitrile Covering the Range between Pyridine and DBU. *J Org Chem* **2000**, *65* (19), 6202–6208.

(10) Perin-Dureau, F.; Rachline, J.; Neyton, J.; Paoletti, P. Mapping the Binding Site of the Neuroprotectant Ifenprodil on NMDA Receptors. *J. Neurosci.* **2002**, *22* (14), 5955–5965.

(11) Mony, L.; Krzaczkowski, L.; Leonetti, M.; Le Goff, A.; Alarcon, K.; Neyton, J.; Bertrand, H.-O.; Acher, F.; Paoletti, P. Structural Basis of NR2B-Selective Antagonist Recognition by N-Methyl-D-Aspartate Receptors. *Mol Pharmacol* **2009**, *75* (1), 60–74.
