# Supplementary material for: Reversible Control of Native GluN2B-Containing NMDA Receptors with Visible Light
Source: ACS Chem Neurosci. 2024 Sep 6;15(18):3321–43. doi: 10.1021/acschemneuro.4c00247 (PMC11413854; doi:10.1021/acschemneuro.4c00247)
Supplement: Supplementary file 3 — cn4c00247_si_003.zip [file cn4c00247_si_003.zip › Dataset-snapshots-QMMM/Inputs-list.pdf]

PNAS

Supporting Information for

Reversible inhibition of GluN2B-containing NMDA receptors with an in situ red-shifted, photodependent antagonist

Chloé Geoffroy, Romain Berraud-Pache, Nicolas Chéron, Isabelle McCort-Tranchepain, Pierre Paoletti, and Laetitia Mony

Corresponding author: Dr. Laetitia Mony,
Email: laetitia.mony@ens.psl.eu

Inputs for QM/MM calculations.

1- Optimization

```
!QMMM D3BJ PBE0 def2-TZVP def2-TZVP/C def2/J RIJCOSX TightSCF opt
%maxcore 5000

%qmmm
QMAtoms {11856:11897} end
activeatoms {11856:11897} end
Dist_AtomsAroundOpt 5
charge_total 0
ORCAFFFilename "test.top.ORCAFF.prms"
end

%pal
nprocs 24
end

*pdbfile 1 1 test.pdb
```

2- TDDFT

```
!QMMM D3BJ RI-B2PLYP def2-TZVP def2-TZVP/C def2/J RIJCOSX TightSCF
%maxcore 5000

%qmmm
QMAtoms {11856:11897} end
activeatoms {11856:11897} end
Dist_AtomsAroundOpt 5
```

```
charge_total 0
ORCAFFFilename "test.top.ORCAFF.prms"
end

%tddft
nroots 8
iroot 1
tda false
end

%pal
nprocs 24
end

*pdbfile 1 1 0.pdb
```

Inputs for calculations in implicit water.

1- Optimization

```
!CPCM(water) D3BJ PBE0 def2-TZVP def2-TZVP/C def2/J RIJCOSX TightSCF
tightopt

%maxcore 5000

%pal
nprocs 24
end

*xyzfile 1 1 test.xyz
```

2- TDDFT

```
!CPCM(water) D3BJ RI-B2PLYP def2-TZVP def2-TZVP/C def2/J RIJCOSX TightSCF
%maxcore 5000

%tddft
nroots 8
iroot 1
tda false
end

%pal
nprocs 24
end
```

*xyzfile 1 1 0.xyz
